# Supplementary material for: Effects of land use and weather on the presence and abundance of mosquito-borne disease vectors in a urban and agricultural landscape in Eastern Ontario, Canada
Source: PLoS One. 2022 Mar 10;17(3):e0262376. doi: 10.1371/journal.pone.0262376 (PMC8912203; doi:10.1371/journal.pone.0262376)
Supplement: S1 File — (PDF) [file pone.0262376.s001.pdf]

## S1 Table. Period of collection

| Julian days | 2017 collect period | 2018 collect period | Identification |
|---|---|---|---|
| 135 to 148 | May 16th – 27th | May 15th – 25th | C1 |
| 149 to 162 | May 30th – June 9th | May 29th – June 7th | C2 |
| 163 to 176 | June 13th – 23th | June 12th – 22nd | C3 |
| 177 to 190 | June 27th – July 8th | June 26th – July 6th | C4 |
| 191 to 204 | July 11th – 21st | July 10th – 19th | C5 |
| 205 to 219 | July 25th – August 4th | July 24th – August 2nd | C6 |
| 220 to 233 | August 8th – 17th | August 8th – 16th | C7 |
| 234 to 247 | August 22nd – 25th | August 21st – 30th | C8 |
| 248 to 271 | | September 5th – 28th | C9 |

## S2 Table. Species list

| Species | In the occurrence model | In the abundance model |
|---|---|---|
| *Aedes cinereus* | x | x |
| *Aedes vexans* | x | x |
| *Anopheles punctipennis* | x | x |
| *Anopheles quadrimaculatus* | x | x |
| *Anopheles walkeri* | x | x |
| *Coquillettidia perturbans* | x | x |
| *Culex pipiens-restuans* | x | x |
| *Culex territans* | x | |
| *Culiseta melanura* | x | |
| *Culiseta minnesotae* | x | |
| *Culiseta morsitans* | x | |
| *Ochlerotatus abserratus* | x | |
| *Ochlerotatus canadensis* | x | x |
| *Ochlerotatus cantator* | x | |
| *Ochlerotatus communis* | x | |
| *Ochlerotatus dorsalis* | x | |
| *Ochlerotatus excrucians* | x | |
| *Ochlerotatus fitchii* | x | |
| *Ochlerotatus intrudens* | x | |
| *Ochlerotatus japonicus* | x | x |
| *Ochlerotatus provocans* | x | x |
| *Ochlerotatus punctor* | x | |
| *Ochlerotatus stimulans* | x | x |
| *Ochlerotatus triseriatus* | x | |
| *Ochlerotatus trivittatus* | x | x |
| *Psorophora ciliata* | x | |
| *Psorophora ferox* | x | |

## S3 Table. Weather and land use variables

| Category | Variable | Biological justification |
|---|---|---|
| **Weather** | Minimum temperature of the day of capture ($Tmin_0$) | Mosquito biology is highly influenced by temperature (ex. Life cycle length, life span, etc.). Studies have demonstrated that increased temperature could lead to substantial increase in mosquito abundance [1]. |
| | Maximum temperature of the day of capture ($Tmax_0$) | |
| | Precipitation temperature of the day of capture ($Prcp_0$) | Larval survival is dependant to aquatic habitat availability which can be influenced by precipitation. Thus we incorporated the precipitation in our model. A lot of precipitation may also alter larvae development. |
| | Average temperature over 5 d average before the day of capture ($Tmin_5$ and $Tmax_5$) and total precipitation over 5 d before capture ($Prcp_5$) | Depending on the species and the environmental conditions, mosquito life cycle duration varies between 1-4 weeks [2, 3], but may last longer in cold weather [4]. |
| | Average temperature over 30 d average before the day of capture($Tmin_{30}$ and $Tmax_{30}$) and total precipitation over 30 d before capture ($Prcp_{30}$) | |
| | Average temperature over 90 d average before the day of capture ($Tmin_{90}$ and $Tmax_{90}$) and total precipitation over 90 d before capture | |
| **Land use** | Proportion of bare | It is expected that bare land would influence negatively the presence and the abundance of mosquito. |
| | Proportion of water | Mosquitos needs water to complete their life cycle. Some mosquito species present affinity to pristine fresh water. Some species larvae develop in river. But all mosquitos have a closer relationship to wetland. |
| | Proportion of wetland | |
| | Proportion of shrubland | The presence of wooded in a given area, including shrubland and forest, also represent important habitat [5, 6] |
| | Proportion of forest | |
| | Proportion of agriculture | Some studies have shown that agriculture [7-9] and urban land use [7, 10, 11] play a role in mosquito communities. |
| | Proportion of urban | |

## S4 Table. Feature attribute values of land use classes according to the annual crop inventories developed by Agriculture and Agri-Food Canada and proposed reclassification

| Number | Existing classes | Proposed reclassification |
|---|---|---|
| 20 | Water | Water |
| 30 | Exposed Land / Barren | Barren |
| 34 | Urban / Developed | Urban |
| 35 | Greenhouses | Agriculture |
| 50 | Shrubland | Shrubland |
| 80 | Wetland | Wetland |
| 110 | Grassland | Agriculture |
| 122 | Pasture / Forages | Agriculture |
| 133 | Barley | Agriculture |
| 136 | Oats | Agriculture |
| 145 | Winter Wheat | Agriculture |
| 146 | Spring Wheat | Agriculture |
| 147 | Corn | Agriculture |
| 158 | Soybeans | Agriculture |
| 167 | Beans | Agriculture |
| 179 | Other vegetables | Agriculture |
| 210 | Coniferous | Forest |
| 220 | Broadleaf | Forest |
| 230 | Mixedwood | Forest |

## S5 Fig. Biweekly dynamics of minimum temperature, maximum temperature and precipitation from March to August in the study region in the period 2017-2018

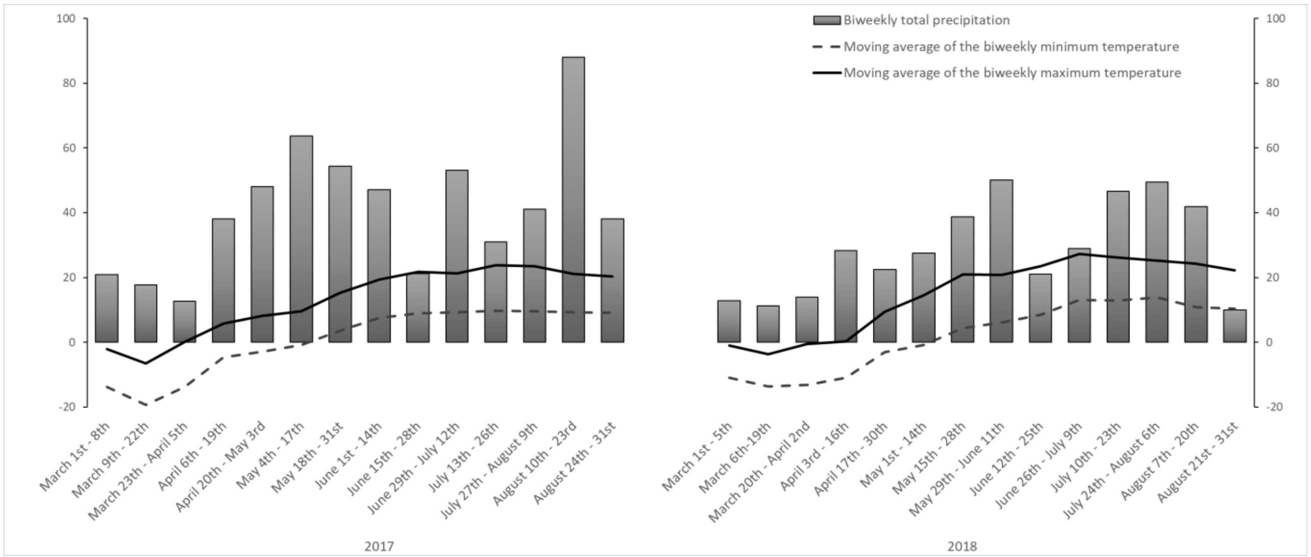

## S6 Table. Species traits

| Species | Number of generations per year | Overwintering stage | Egg laying substrate | Egg resistance to desiccation | Typical larval habitat | | | | |
|---|---|---|---|---|---|---|---|---|---|
| | | | | | Wetland habitat | Non-wooded habitat (except urban) | Wooded habitat | Urban habitat | Plant-dependant species |
| *Ae. cinereus* | Multivoltine | Egg | Aquatic | Resistant | Yes | Yes | No | No | No |
| *Ae. vexans* | Multivoltine | Egg | Aquatic | Resistant | Yes | Yes | Yes | No | No |
| *An. punctipennis* | Multivoltine | Adult | Aquatic | Non resistant | Yes | No | Yes | Yes | No |
| *An. walker* | Multivoltine | Egg | Aquatic | Non resistant | Yes | Yes | No | No | No |
| *Cq. perturbans* | Univoltine | Larvae | Aquatic | Non resistant | Yes | No | Yes | No | No |
| *Cs. melanura* | Multivoltine | Larvae | Aquatic | Non resistant | Yes | No | Yes | No | No |
| *Cs. minnesotae* | Univoltine | Adult | Aquatic | Resistant | Yes | No | No | No | Yes |
| *Cs. morsitans* | Univoltine | Egg | Aquatic | Resistant | Yes | Yes | Yes | No | No |
| *Cx. pipiens-restuans* | Multivoltine | Adult | Aquatic | Non resistant | Yes | No | Yes | Yes | No |
| *Cx. territans* | Multivoltine | Adult | Aquatic | Non resistant | Yes | No | Yes | Yes | No |
| *Oc. abserratus* | Univoltine | Egg | Aquatic | Resistant | Yes | No | No | No | Yes |
| *Oc. canadensis* | Univoltine | Egg | Aquatic | Resistant | Yes | No | Yes | No | No |
| *Oc. cantator* | Multivoltine | Egg | Aquatic | Resistant | Yes | Yes | No | No | No |
| *Oc. communis* | Univoltine | Egg | Solid substrate | Resistant | Yes | No | Yes | No | No |
| *Oc. dorsalis* | Multivoltine | Egg | Aquatic | Resistant | Yes | Yes | No | No | No |
| *Oc. excrucians* | Univoltine | Egg | Solid substrate | Resistant | Yes | Yes | No | No | No |
| *Oc. fitchii* | Univoltine | Egg | Aquatic | Resistant | Yes | Yes | Yes | No | No |
| *Oc. intrudens* | Univoltine | Egg | Solid substrate | Resistant | Yes | No | Yes | No | No |
| *Oc. japonicas* | Multivoltine | Egg | Aquatic | Resistant | Yes | No | Yes | Yes | No |
| *Oc. provocans* | Univoltine | Larvae | Solid substrate | Resistant | Yes | Yes | Yes | No | No |
| *Oc. punctor* | Multivoltine | Adult | Solid substrate | Resistant | Yes | No | Yes | No | No |
| *Oc. quadrimaculatus* | Multivoltine | Adult | Aquatic | Non resistant | Yes | Yes | No | Yes | No |
| *Oc. stimulans* | Univoltine | Egg | Solid substrate | Resistant | Yes | No | Yes | No | No |
| *Oc. triseriatus* | Multivoltine | Egg | Aquatic | Resistant | Yes | No | Yes | No | No |
| *Oc. trivitattus* | Univoltine | Egg | Solid substrate | Resistant | Yes | Yes | Yes | No | No |
| *Ps. ciliata* | Multivoltine | Egg | Aquatic | Resistant | Yes | No | Yes | No | No |
| *Ps. ferox* | Multivoltine | Egg | Aquatic | Resistant | Yes | No | Yes | No | No |

**S7 Fig. Logarithm of the biweekly abundance of the female adult mosquitos captured in Ottawa and the South Nation watershed (SN), Canada (2017 and 2018).**

The abundance of five species (*Oc. abserratus*, *Oc. fitchii, Cx. territans, Cs. minnesotae, Ps. ferox*) was not illustrated due to low count.

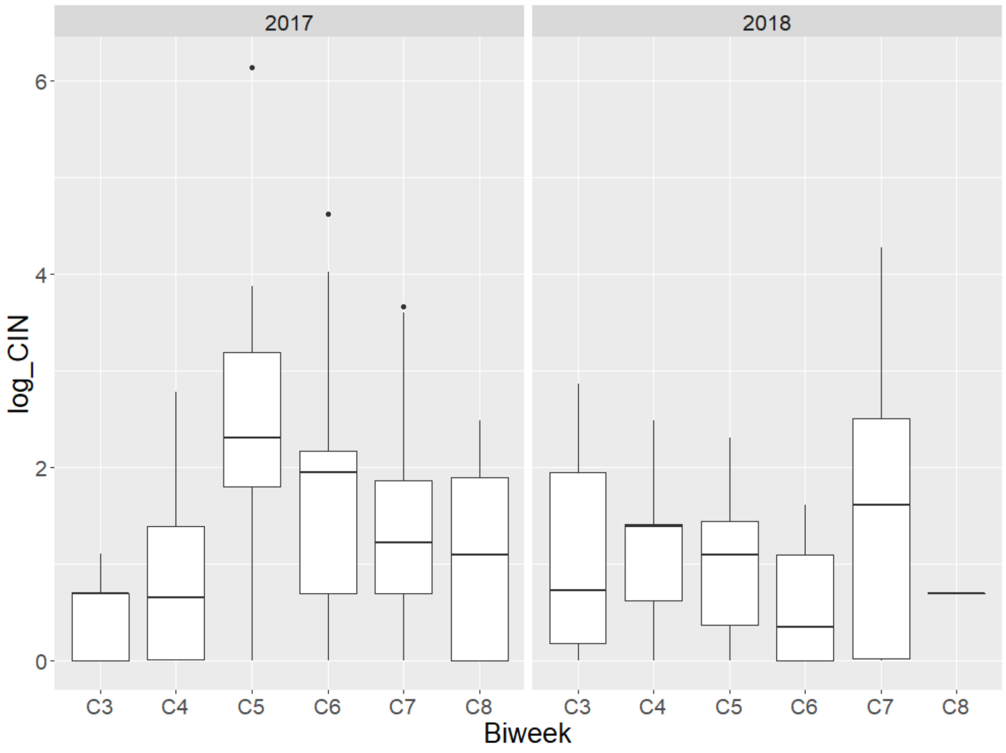


*Ae. cinereus* (CIN)

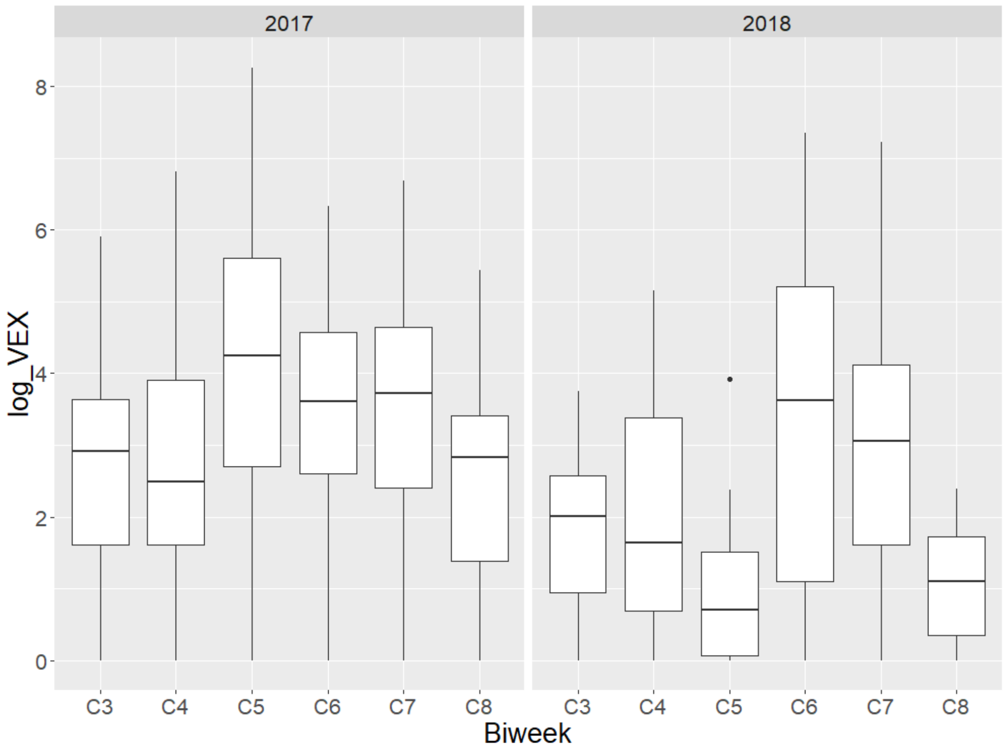


*Ae. vexans* (VEX)

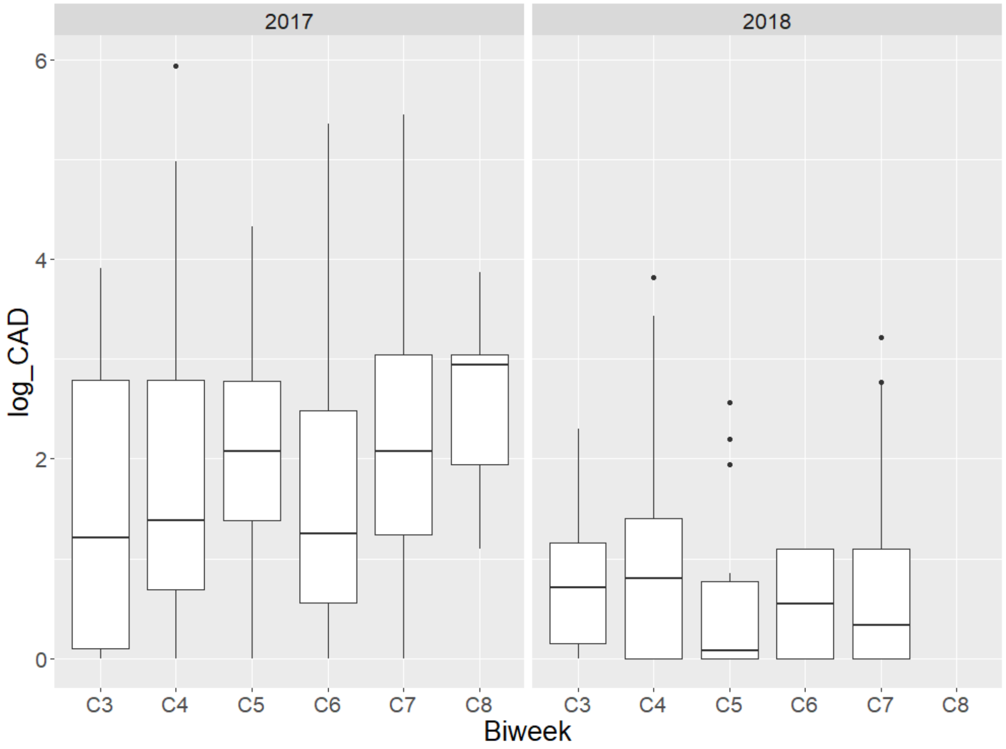


*Oc. canadensis* (CAD)

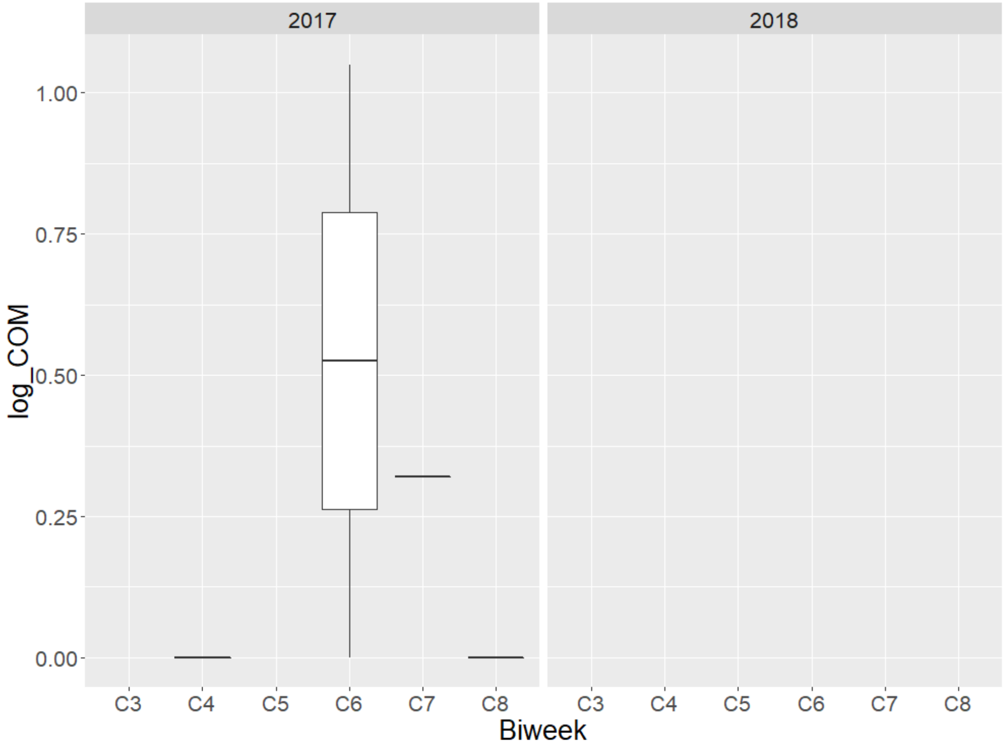


*Oc. communis* (COM)

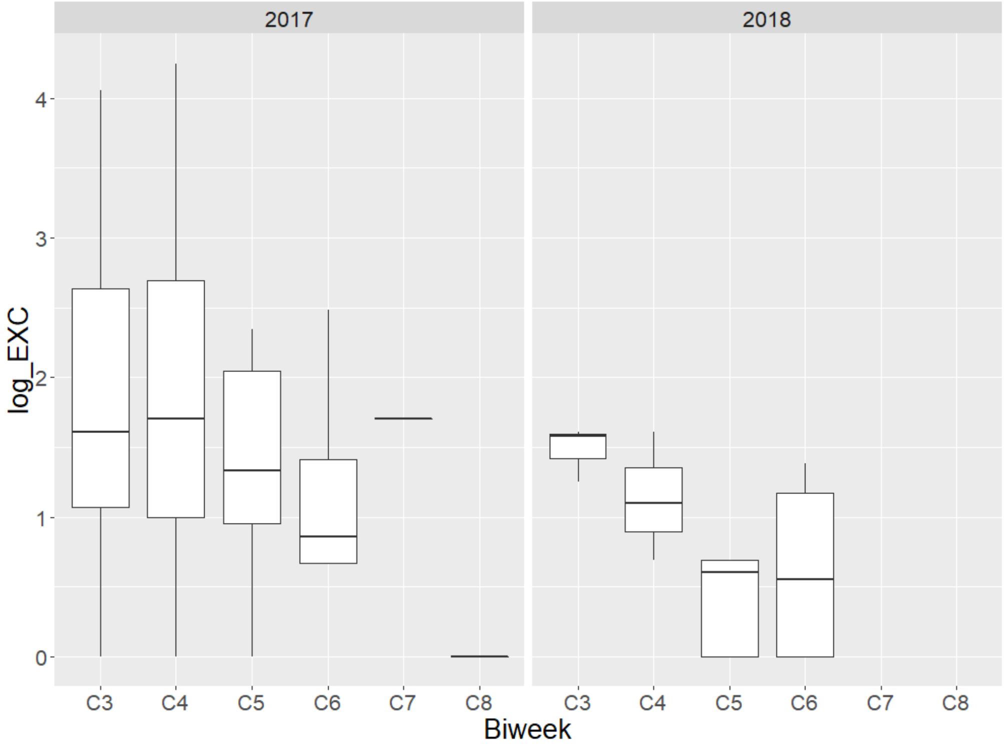


*Oc. excrucians* (EXC)

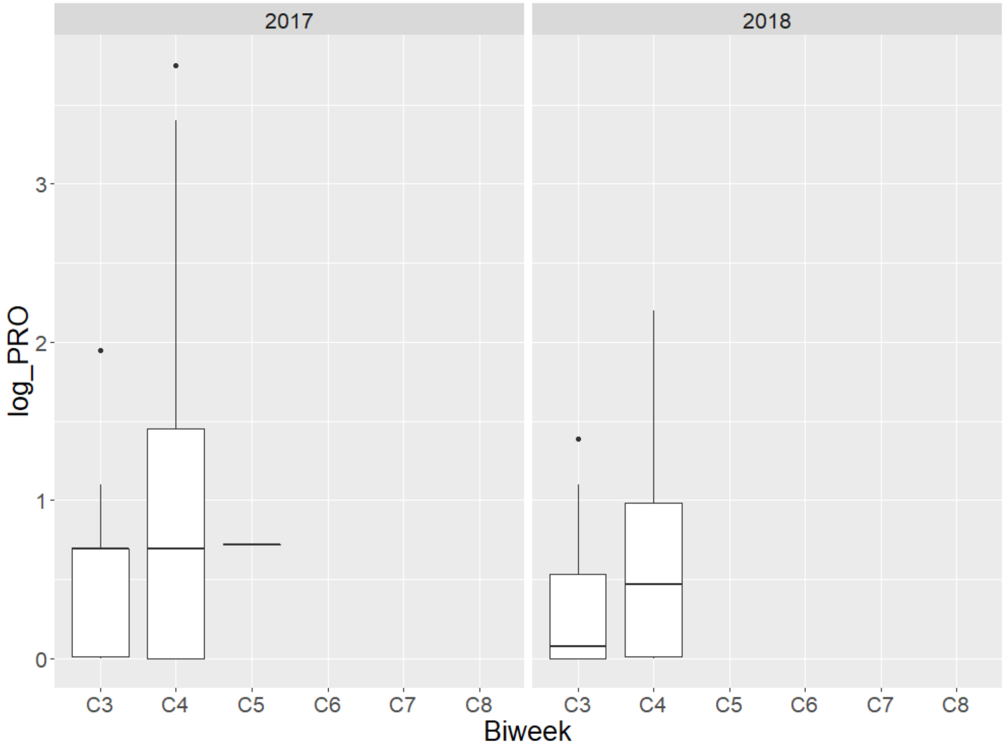


*Oc. provocans* (PRO)

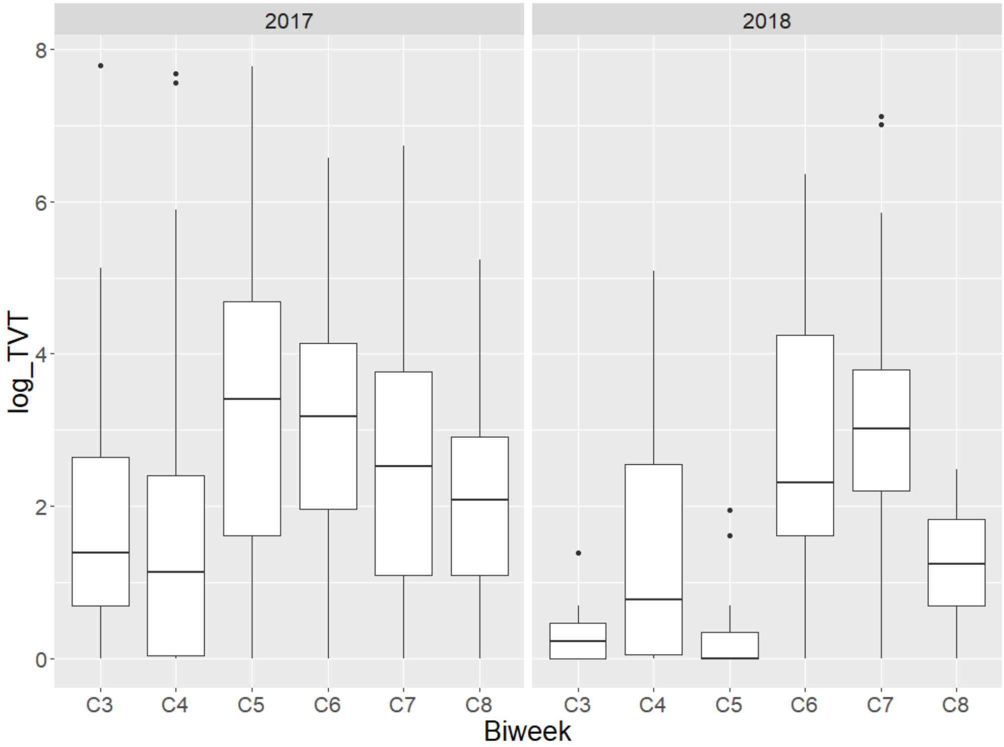


*Oc. trivittatus* (TVT)

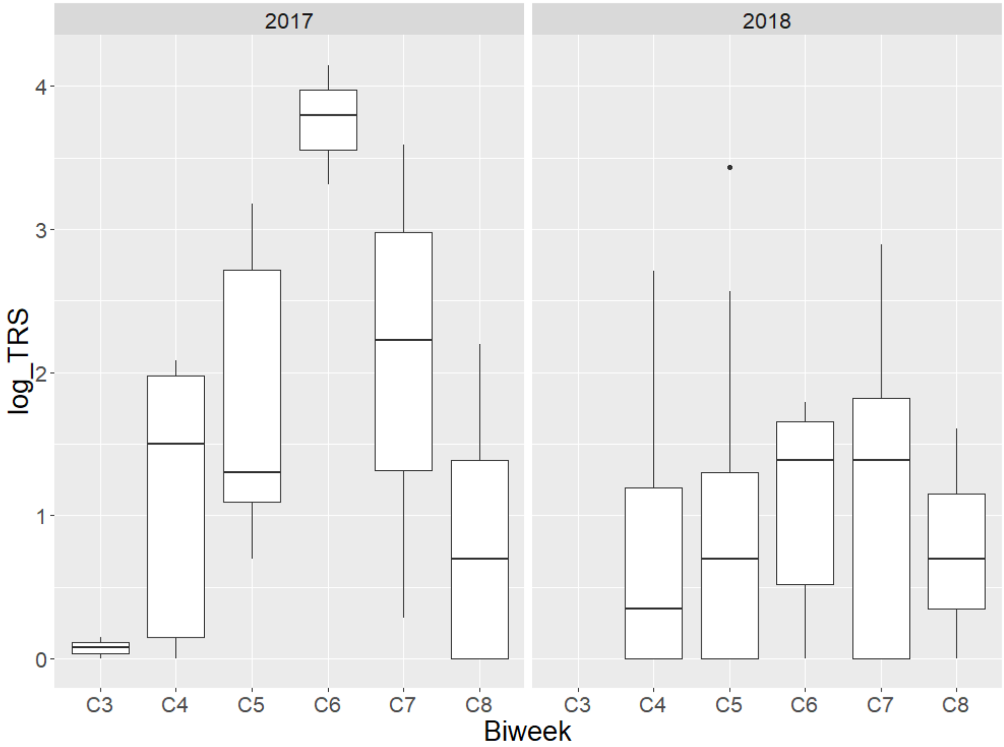


*Oc. triseriatus* (TRS)

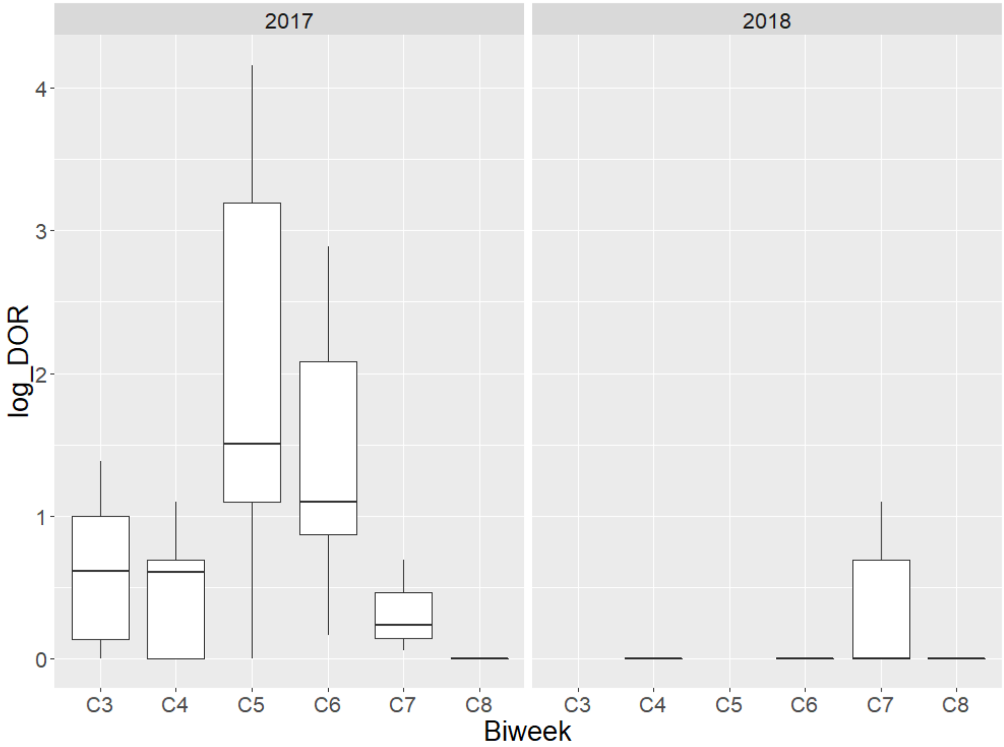


*Oc. dorsalis* (DOR)

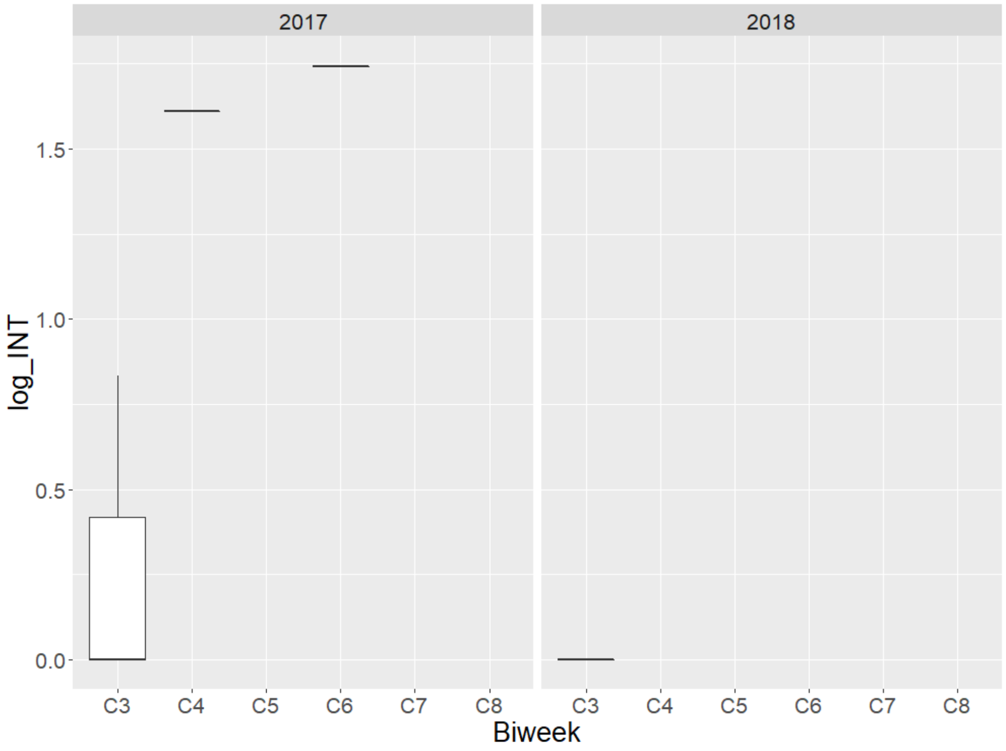


*Oc. intrudens* (INT)

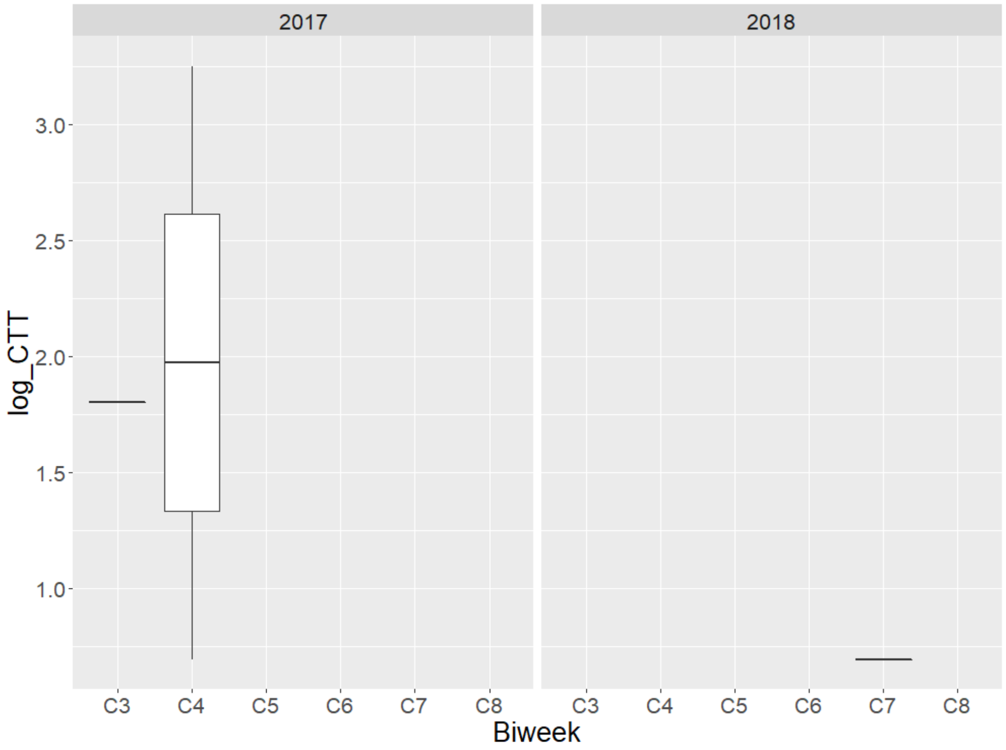


*Oc. cantator* (CTT)

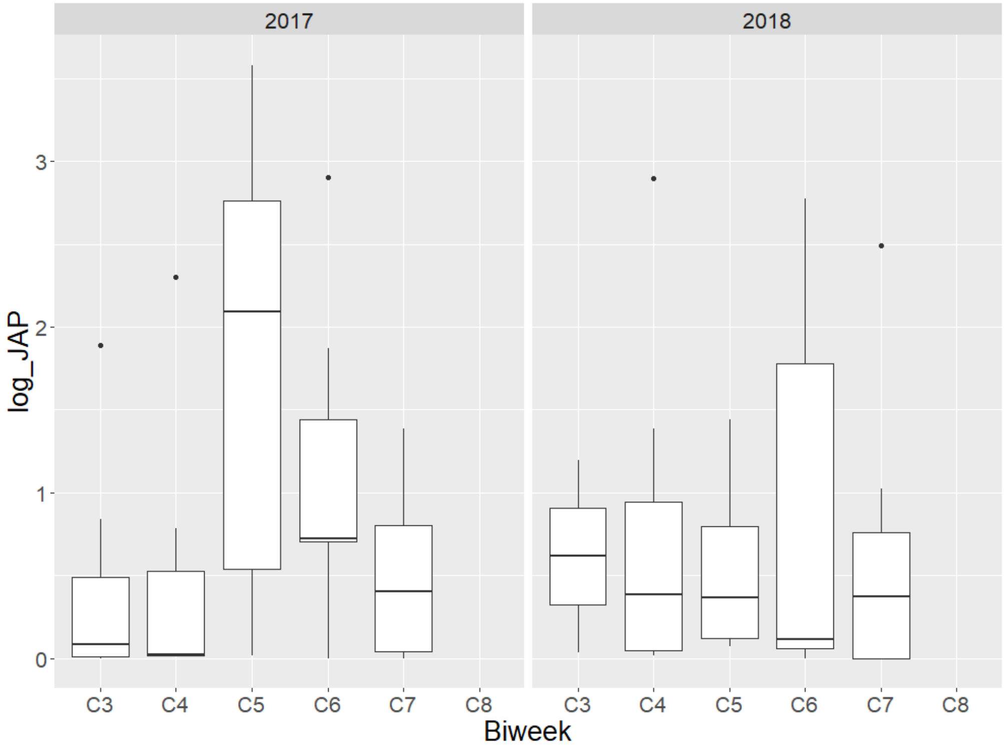

*Oc. japonicus* (JAP)

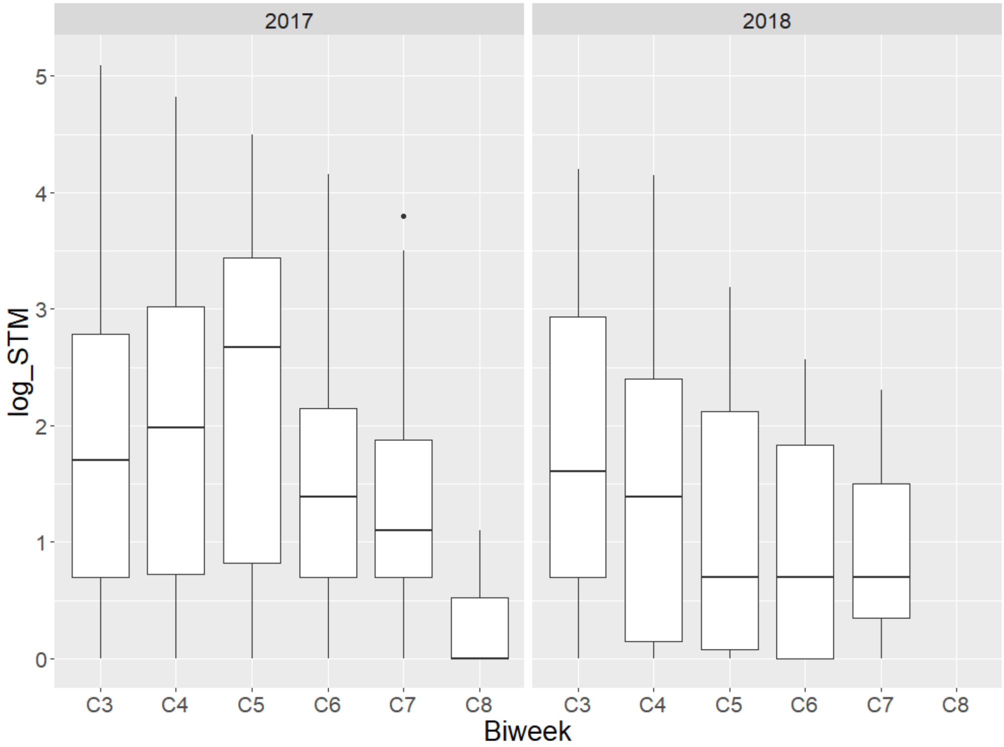

*Oc. stimulans* (STM)

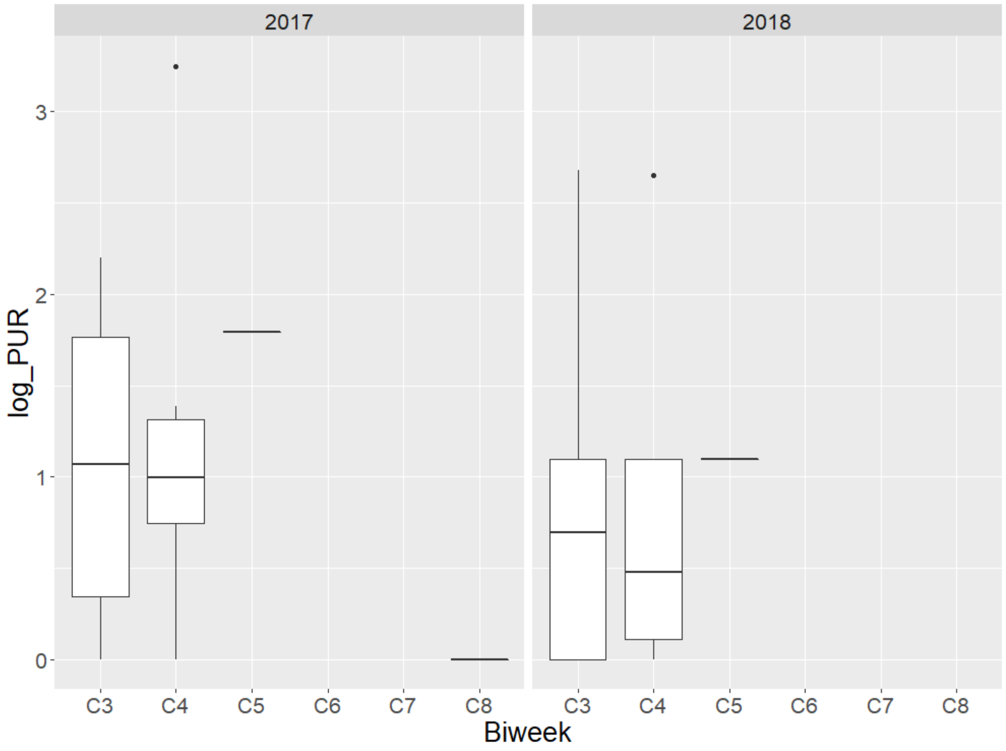


*Oc. punctor* (PUR)

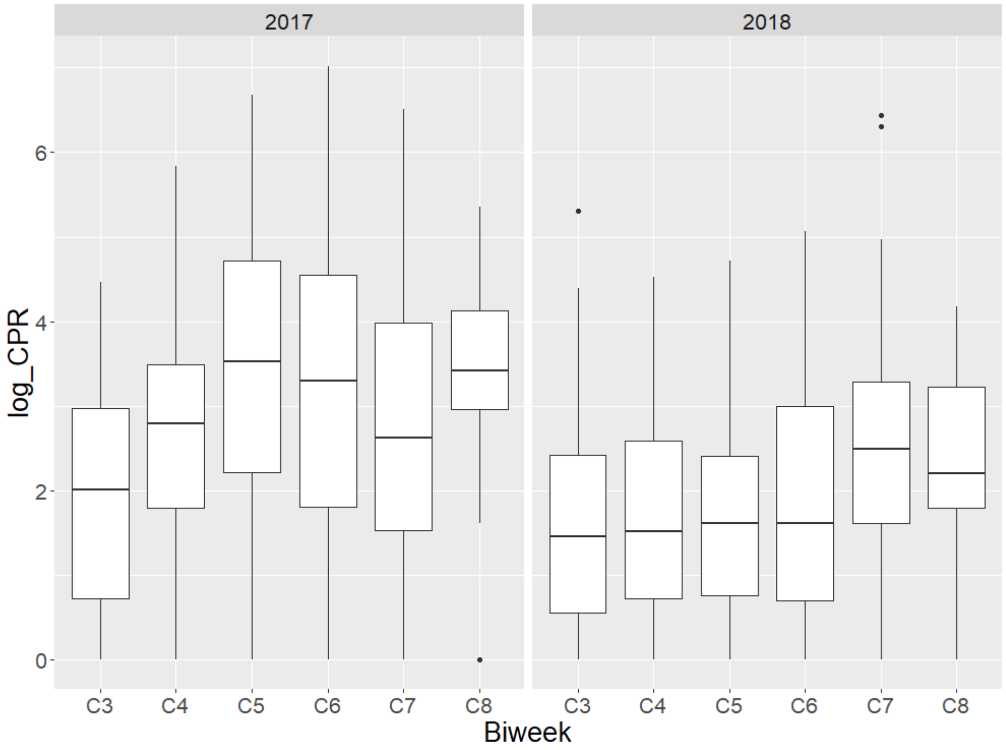


*Cx. pipiens-restuans*(CPR)

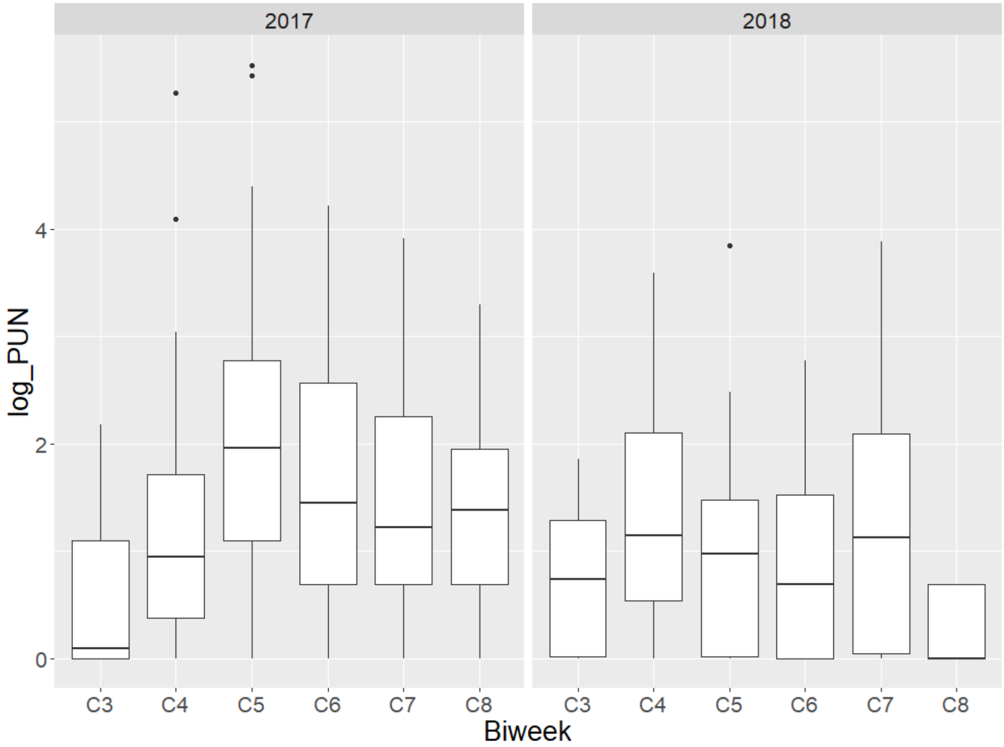

*An. punctipennis* (PUN)

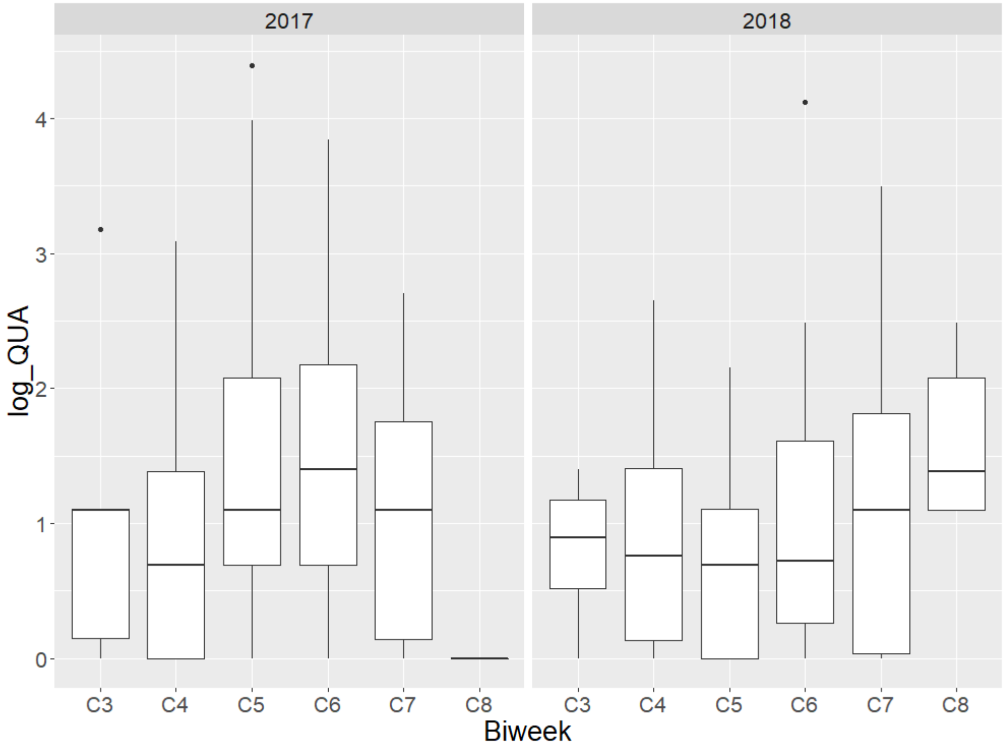

*An. quadrimaculatus* (QUA)

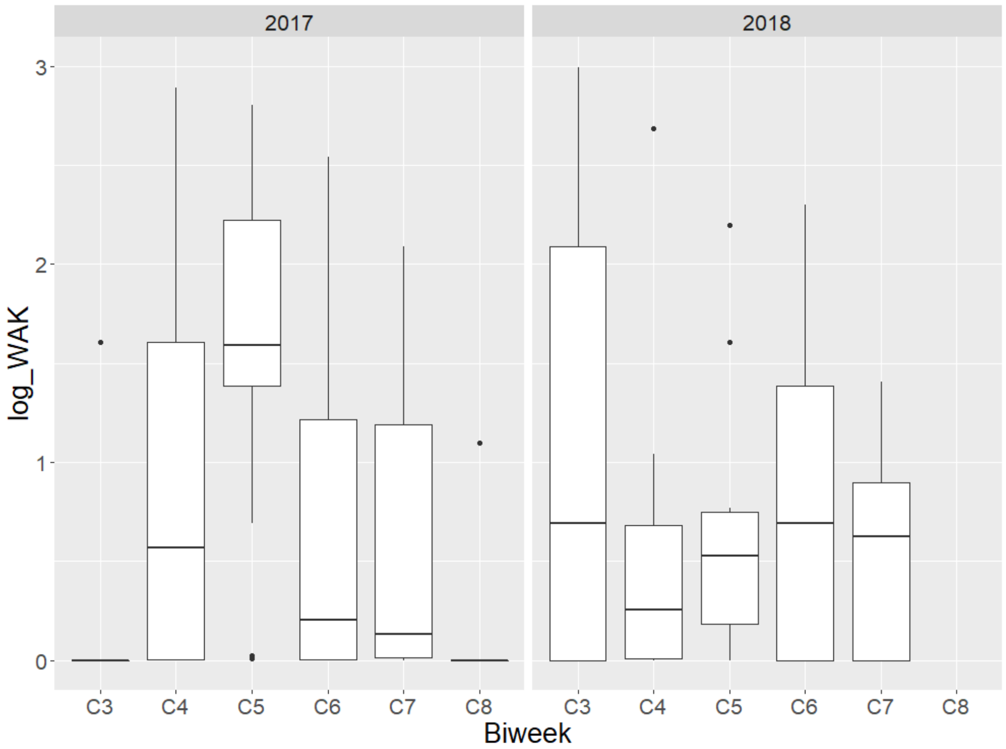


*An. walkeri* (WAK)

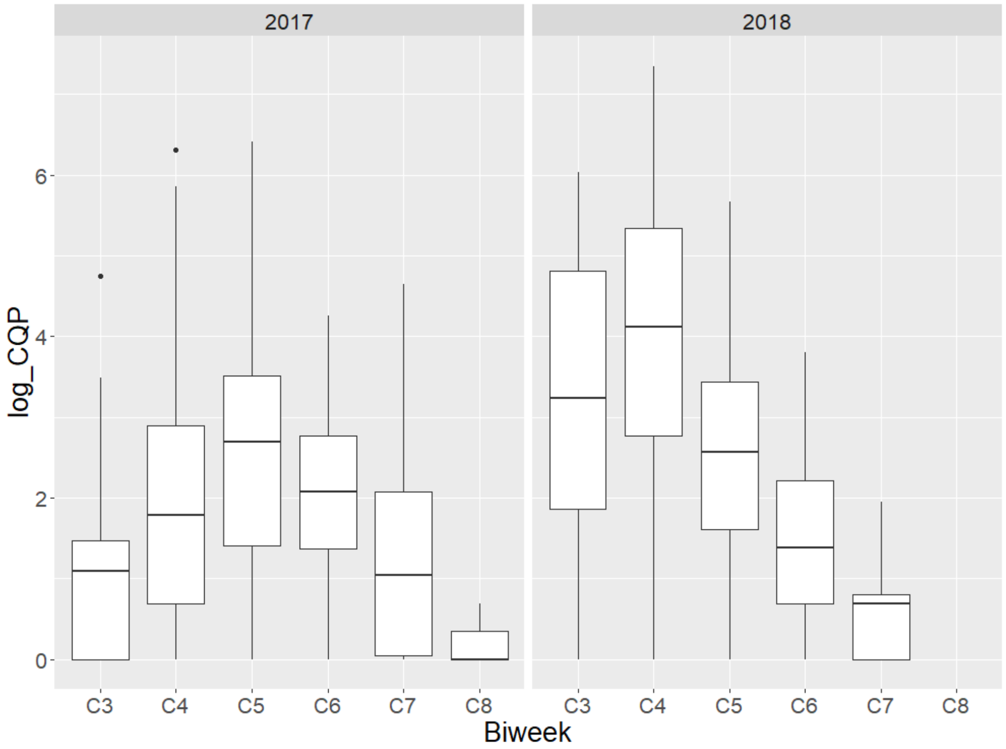


*Cq. perturbans* (CQP)

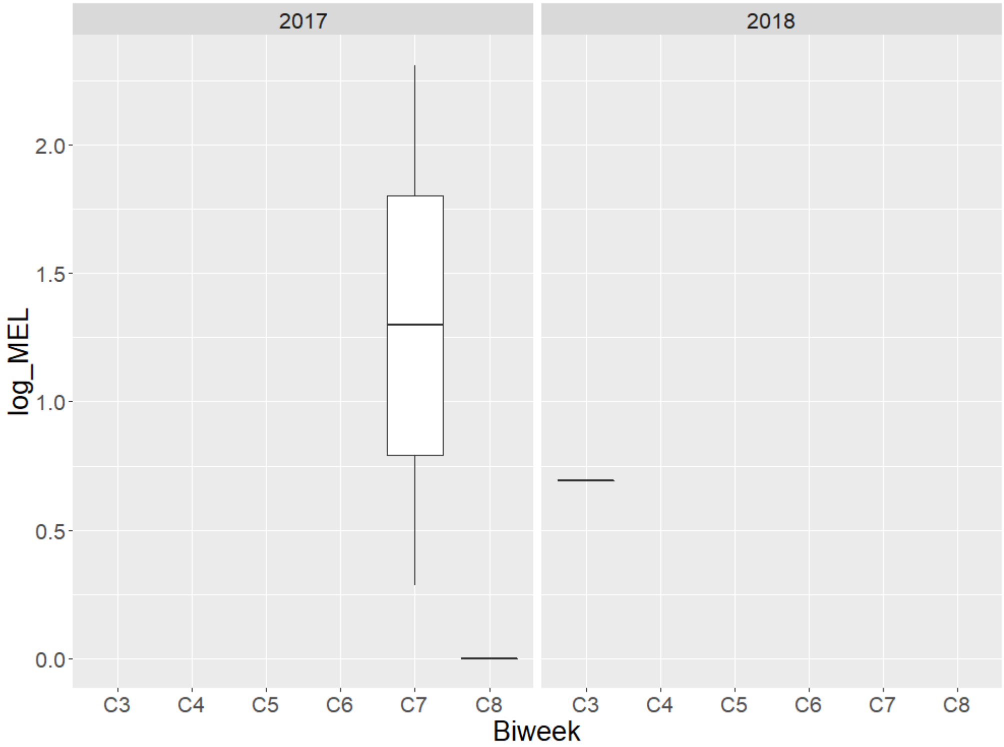


*Cs. melanura* (MEL)

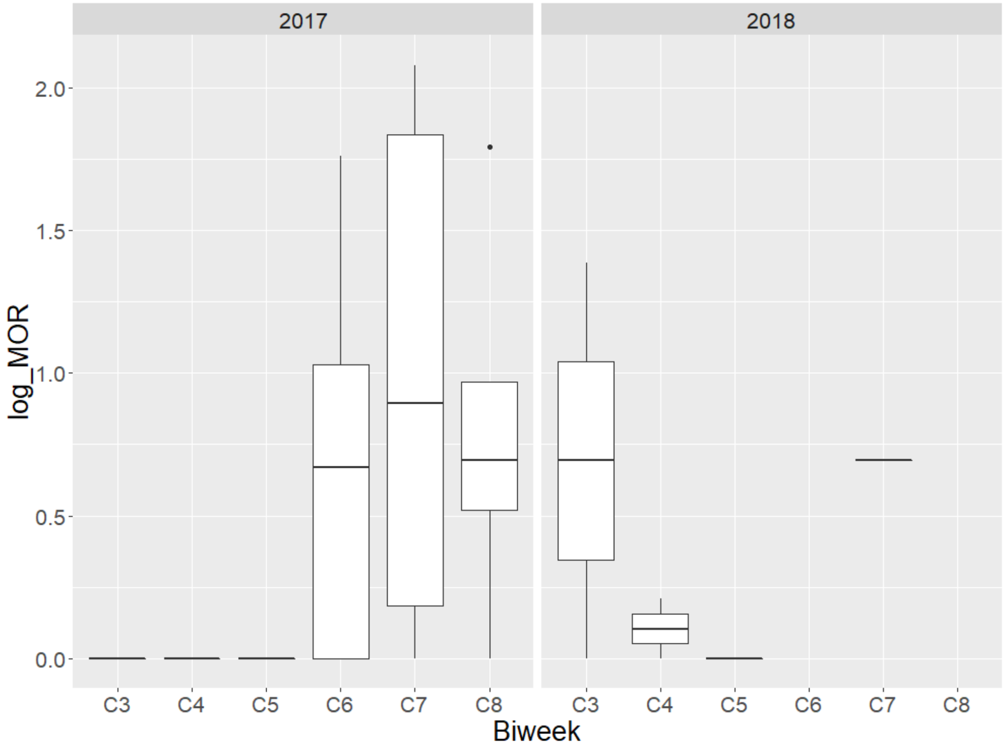


*Cs. morsitans* (MOR)

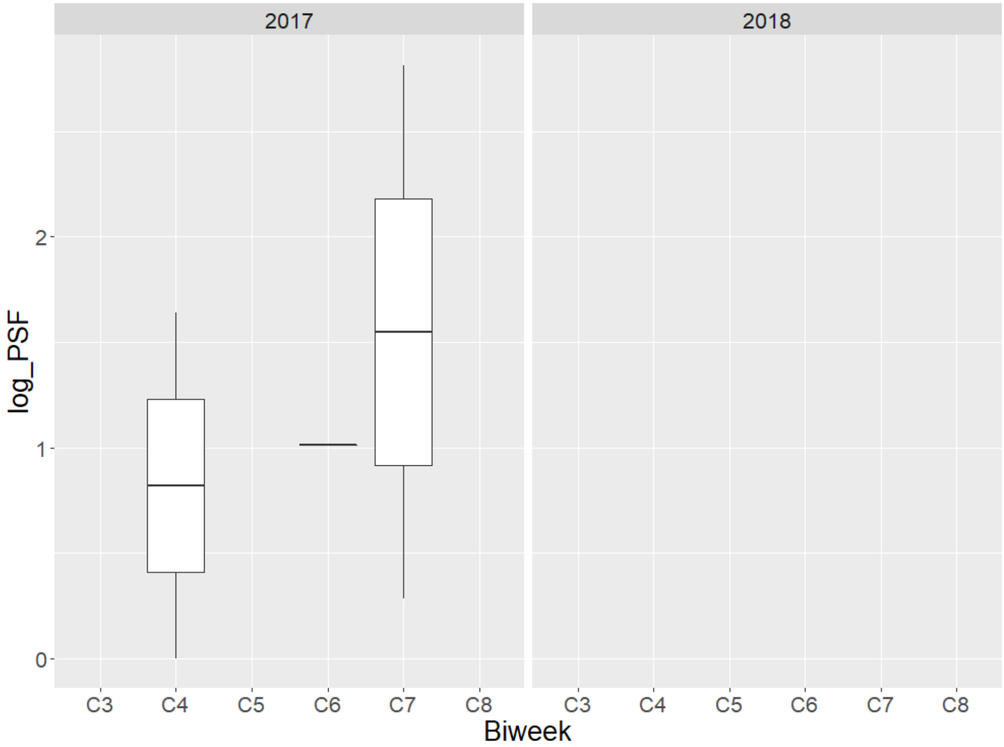


*Ps. ferox*(MEL)

**S8 Fig. Violin plots showing the marginal distribution of the weather parameters for each species resulting from the weather-and-land-use occurrence model. The short segments represent the MCMC iterations for each parameter.**

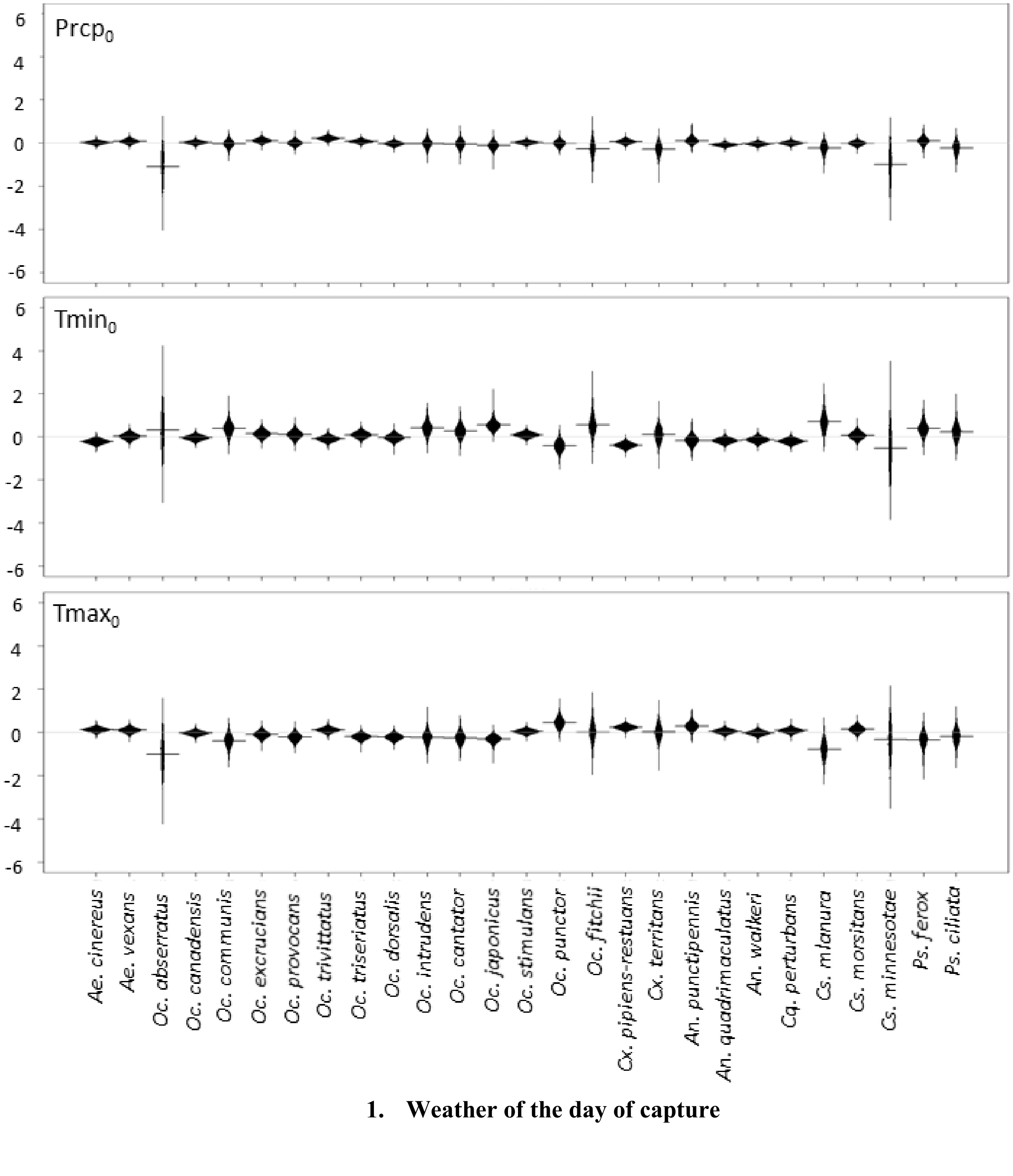


1. **Weather of the day of capture**

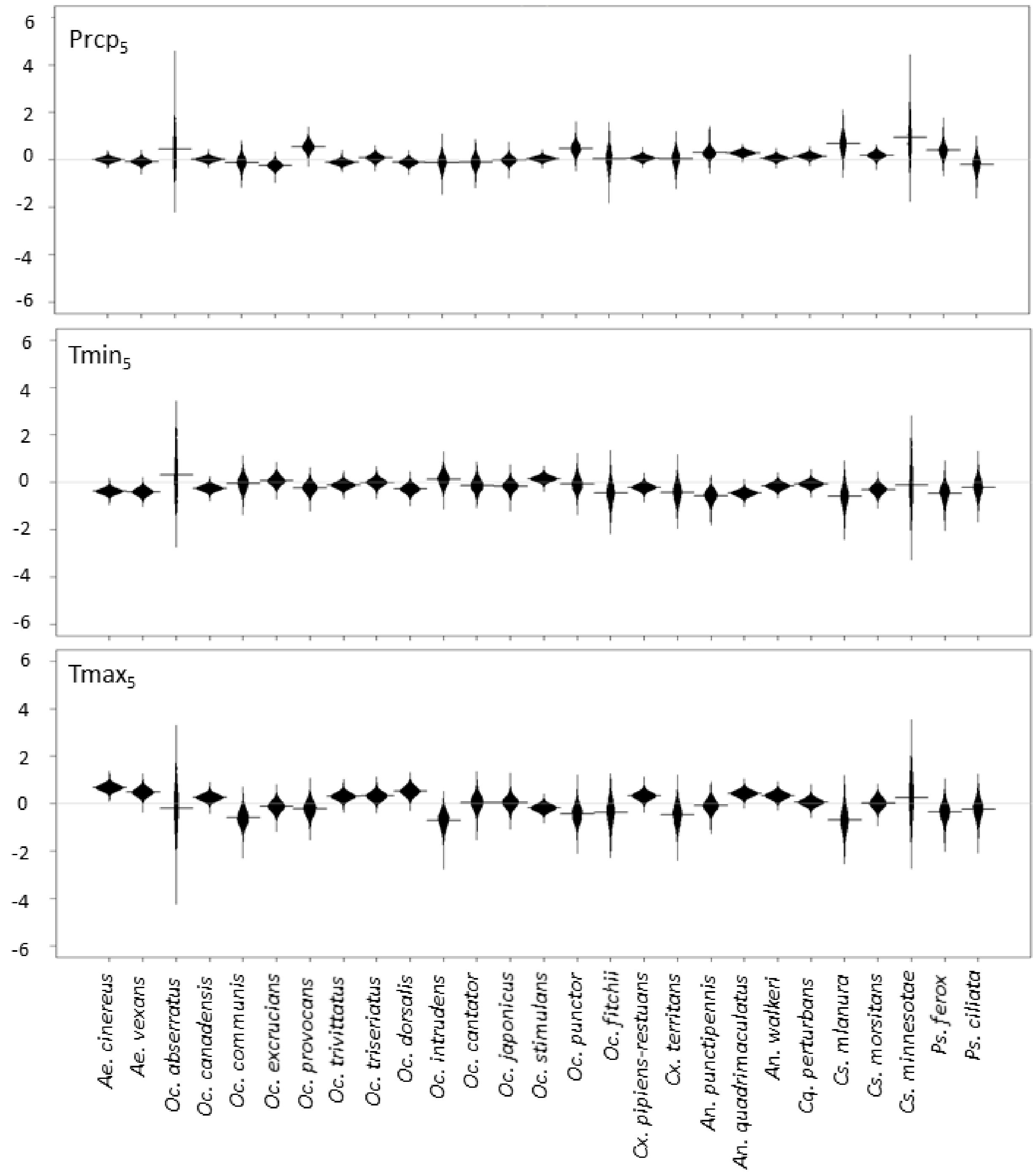


**2. Weather averaged over 5d before capture**

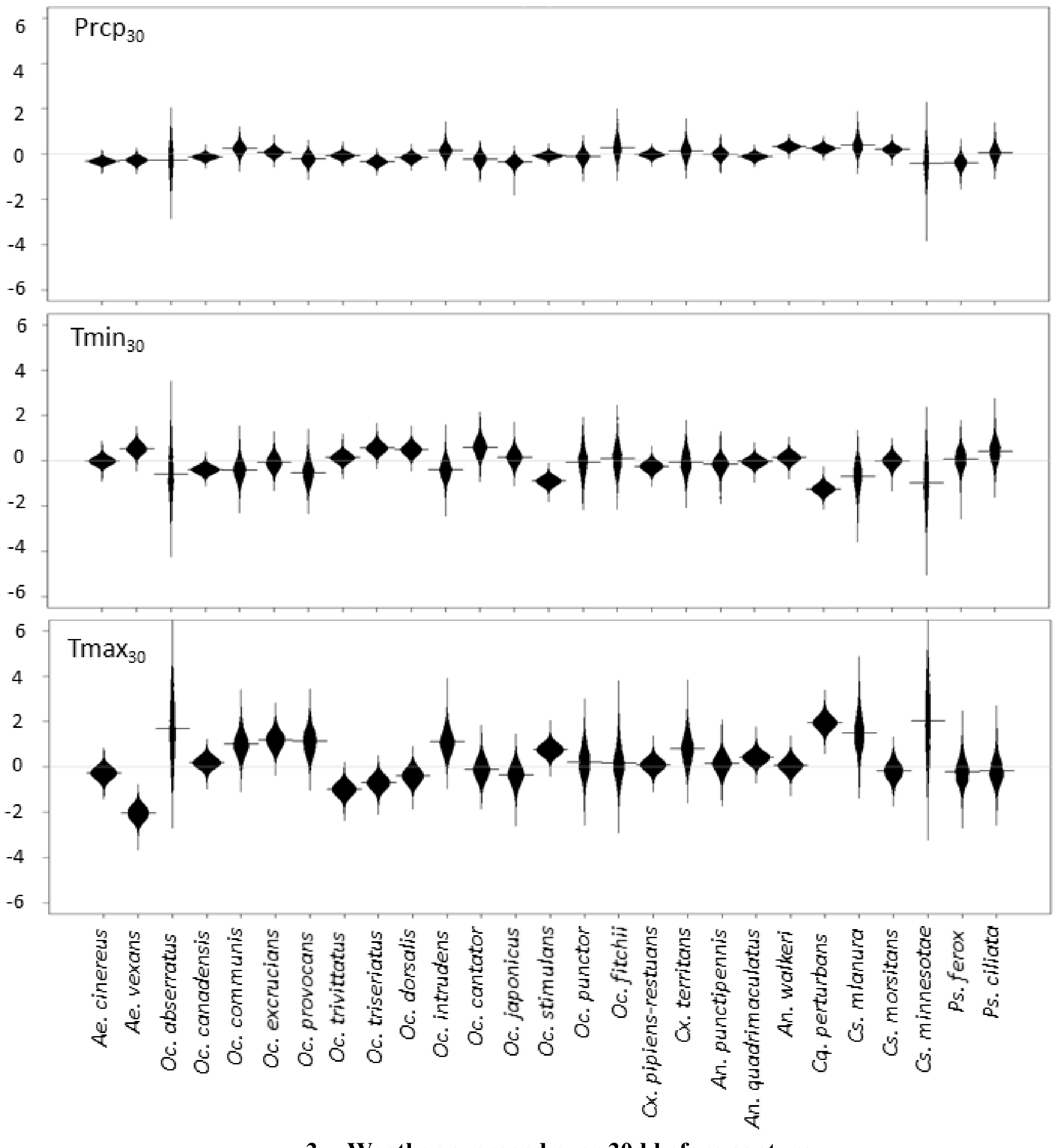


**3. Weather averaged over 30d before capture**

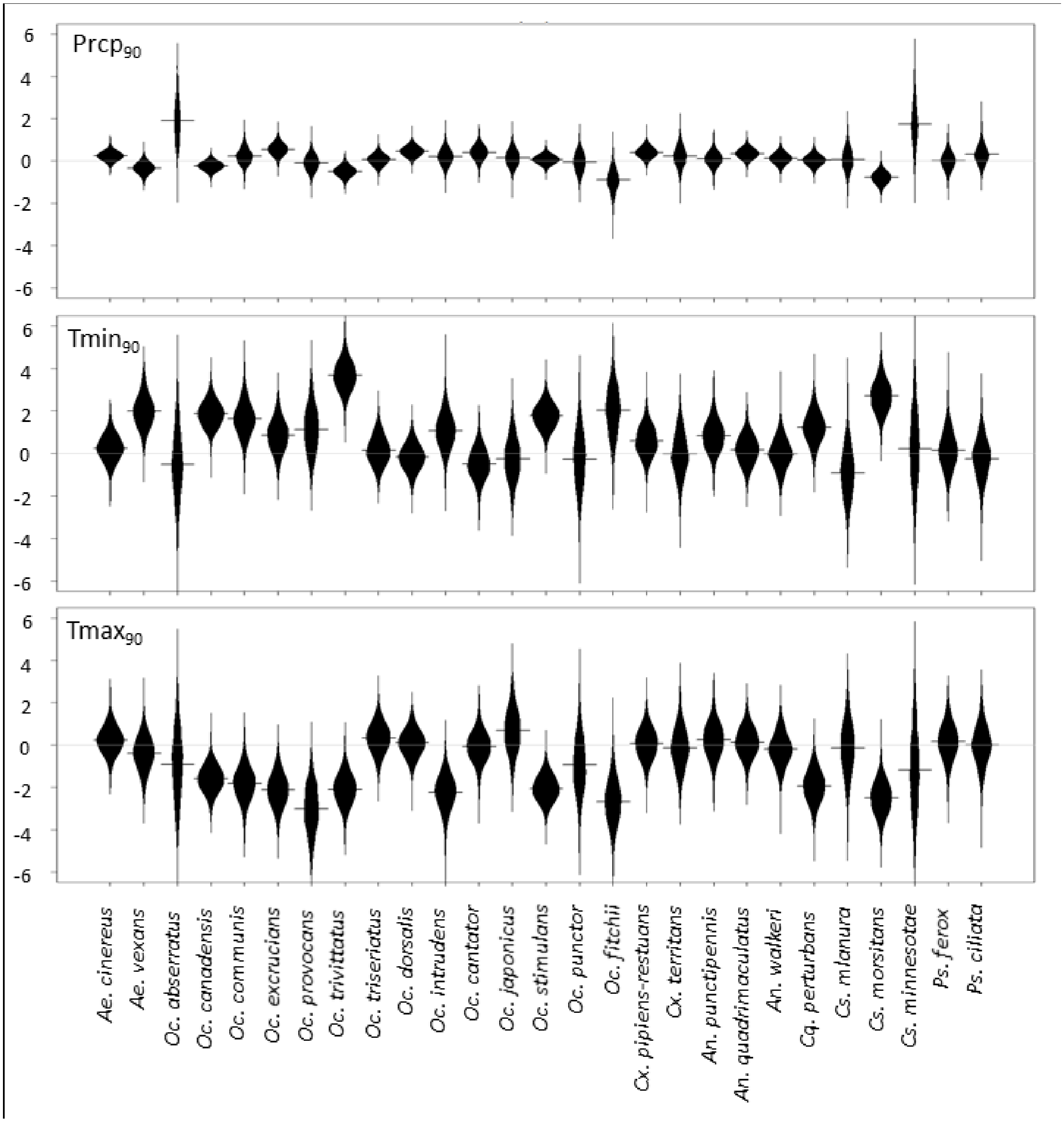


**4. Weather averaged over 90d before capture**

**S9 Fig. Violin plots showing the marginal distribution of the weather parameters for each species resulting from the weather-and-land-use abundance model. The short segments represent the MCMC iterations for each parameter.**

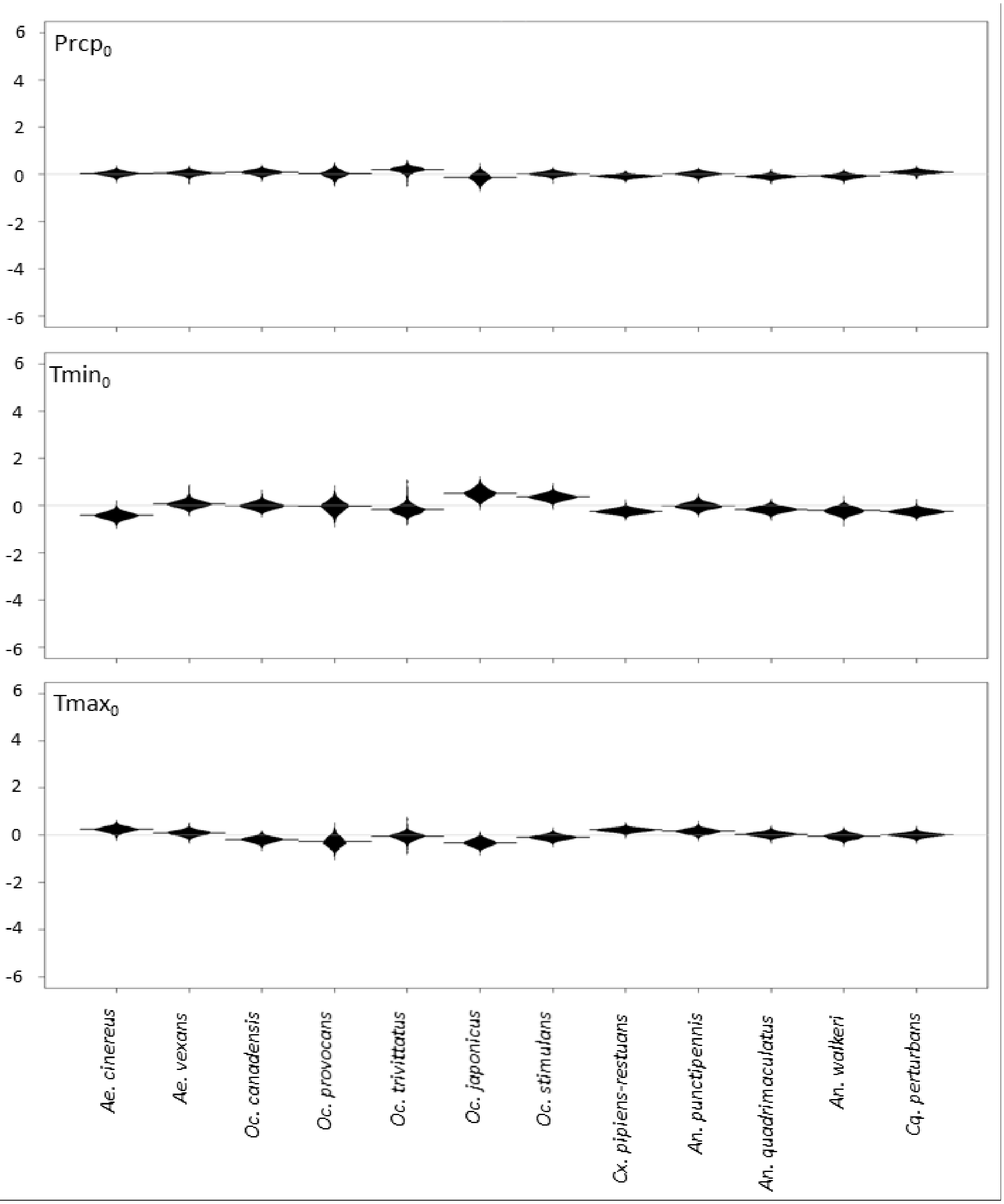


1. **Weather of the day of capture**

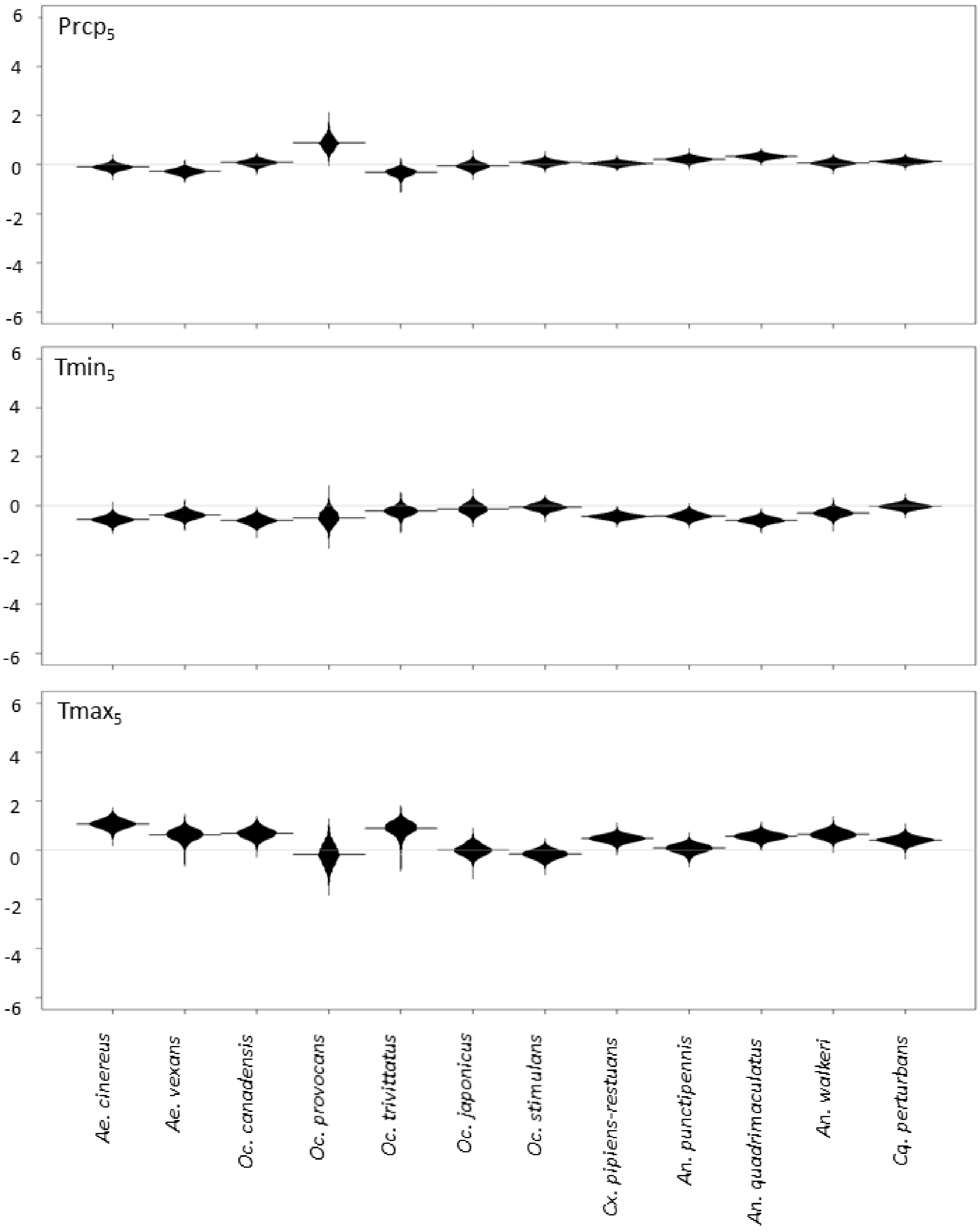


**2. Weather averaged 5d before capture**

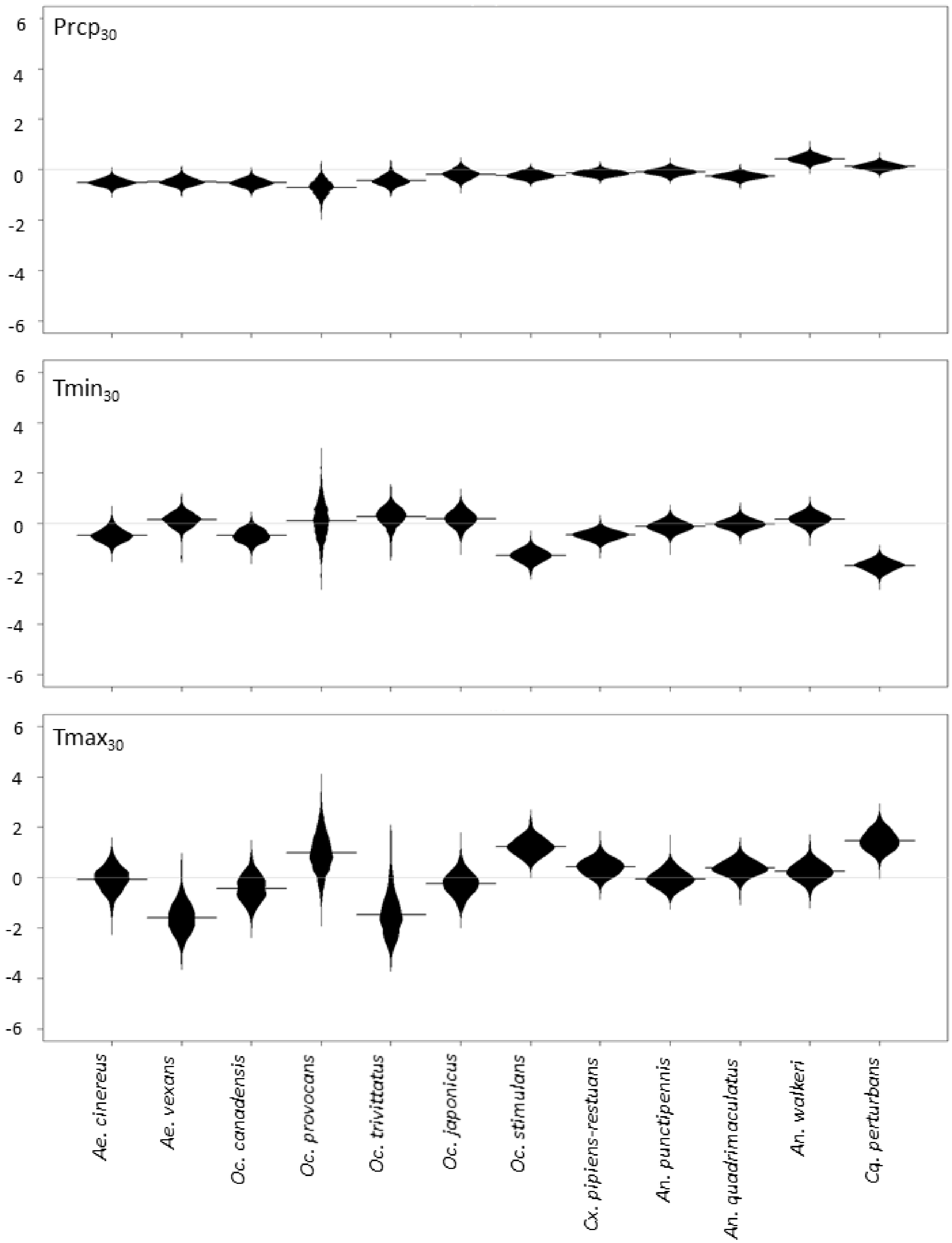


**3. Weather averaged 30d before capture**

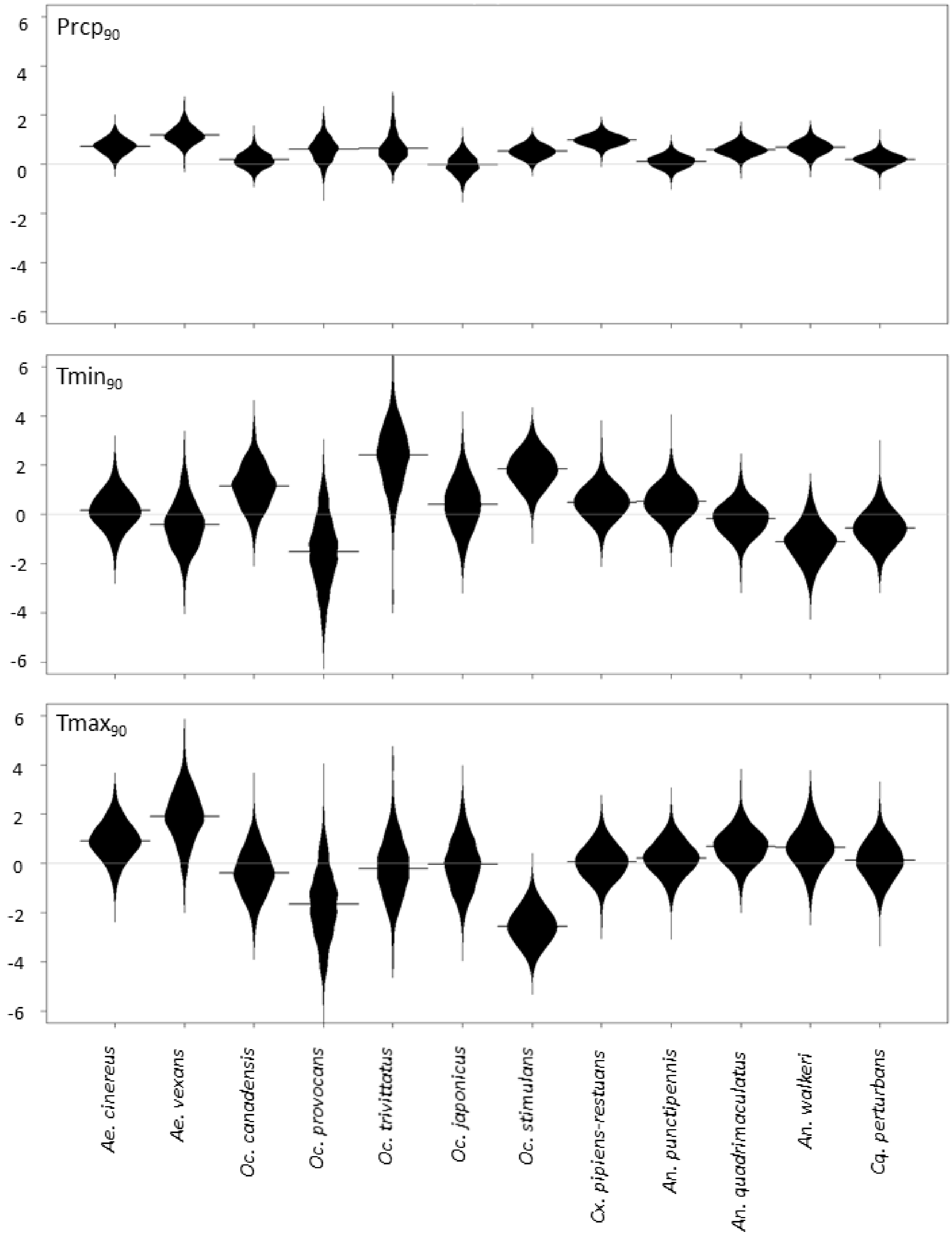


**4. Weather averaged 90d before capture**

**S10 Fig. Violin plots showing the marginal distribution of the land use parameters for each species resulting from the weather-and-land-use occurrence model. The short segments represent the MCMC iterations for each parameter.**

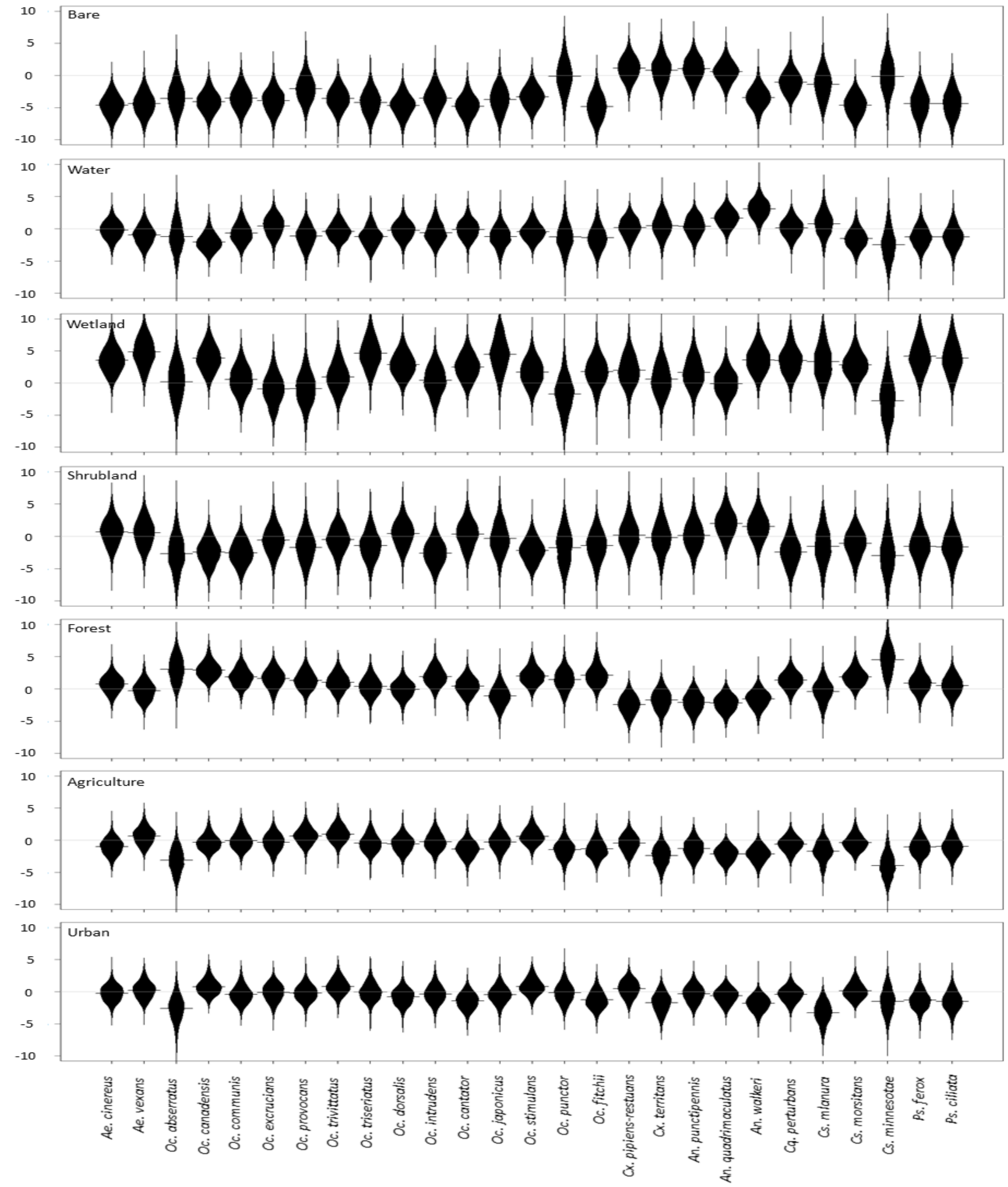

**S11 Fig. Violin plots showing the marginal distribution of the land use parameters for each species resulting from the weather-and-land-use abundance model. The short segments represent the MCMC iterations for each parameter.**

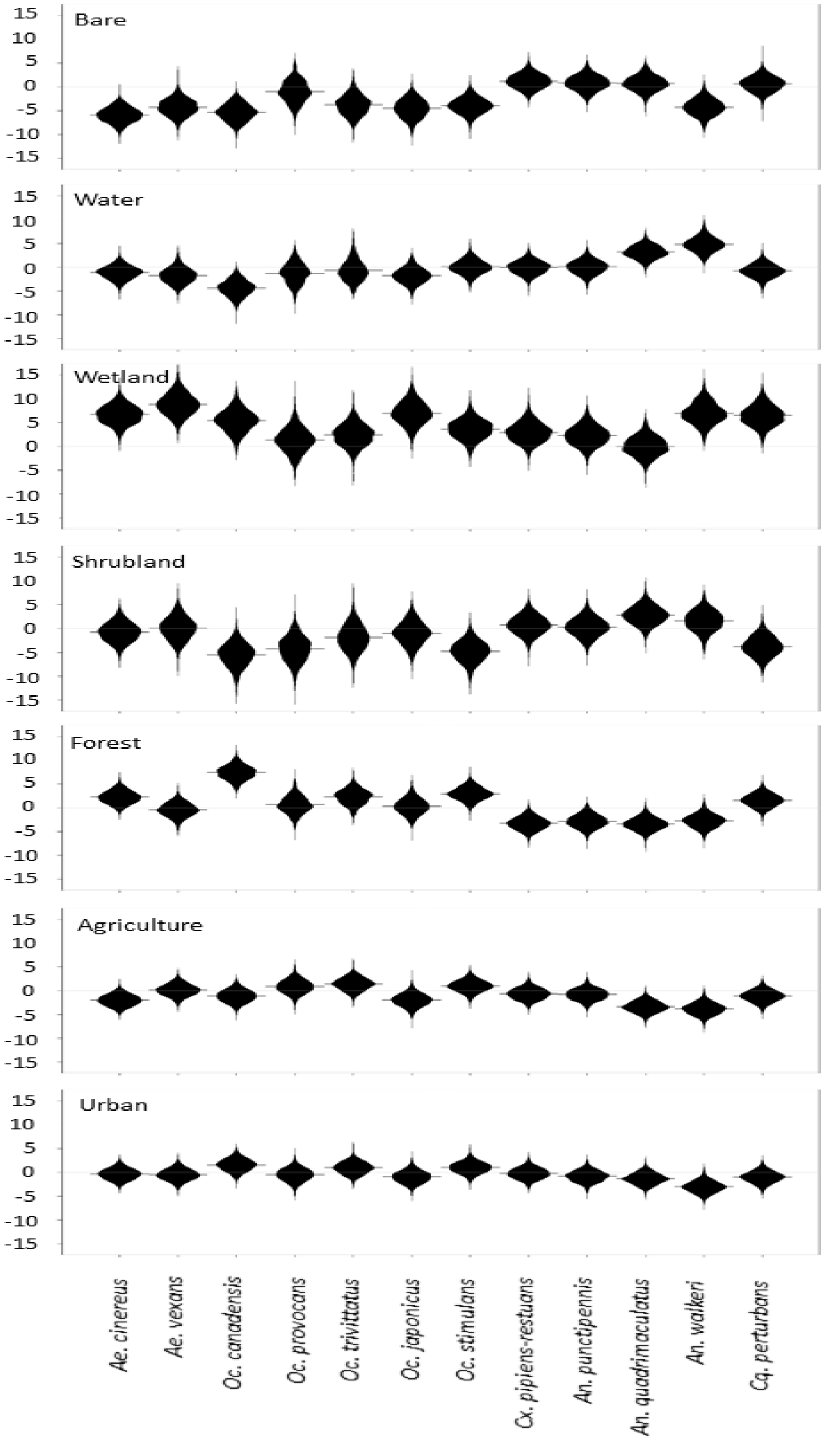

**S12 Fig. Violin plots showing the marginal distribution of the weather parameters for each species resulting from the weather-only occurrence model. The short segments represent the MCMC iterations for each parameter.**

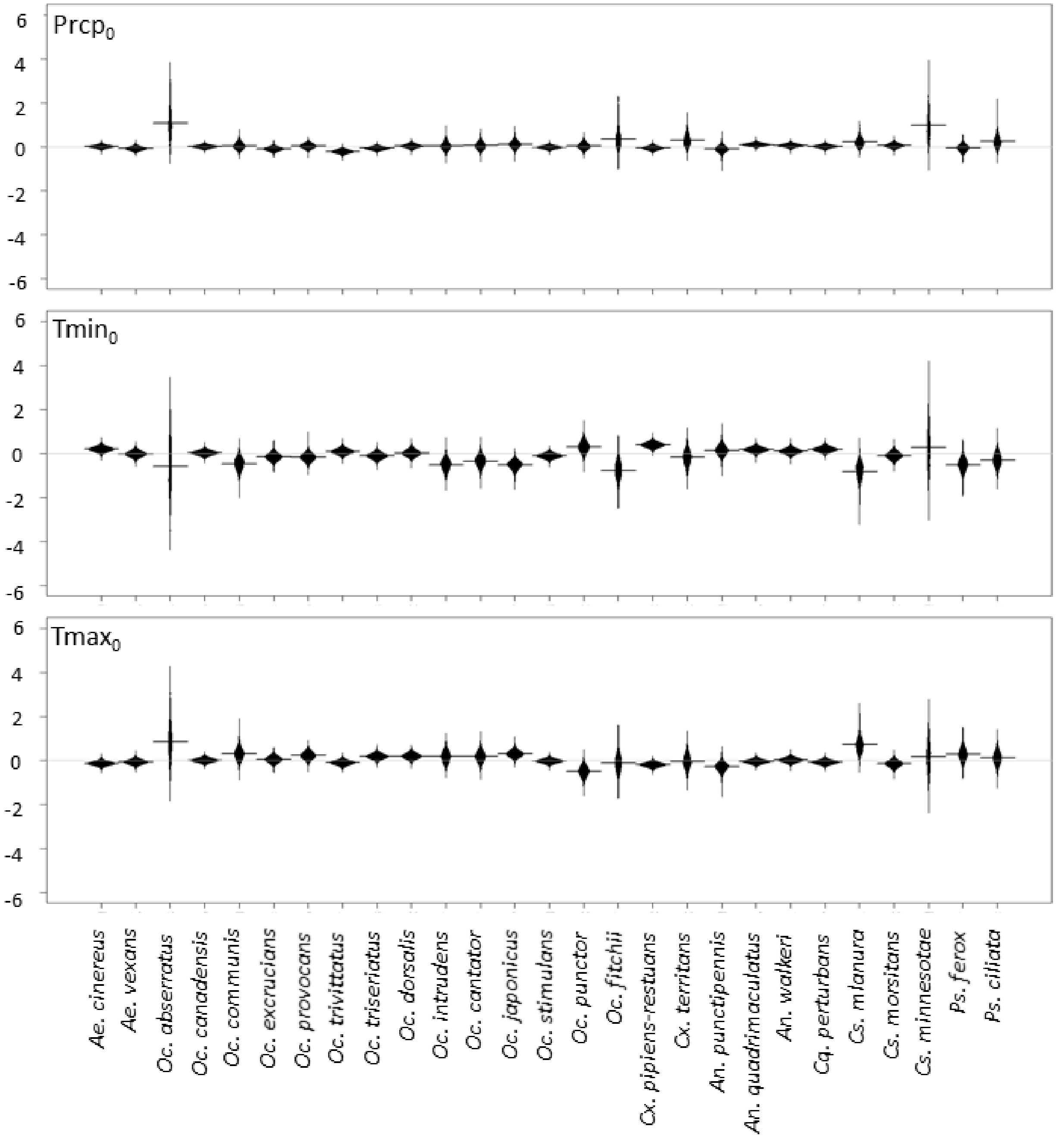


1. **Weather of the day of capture**

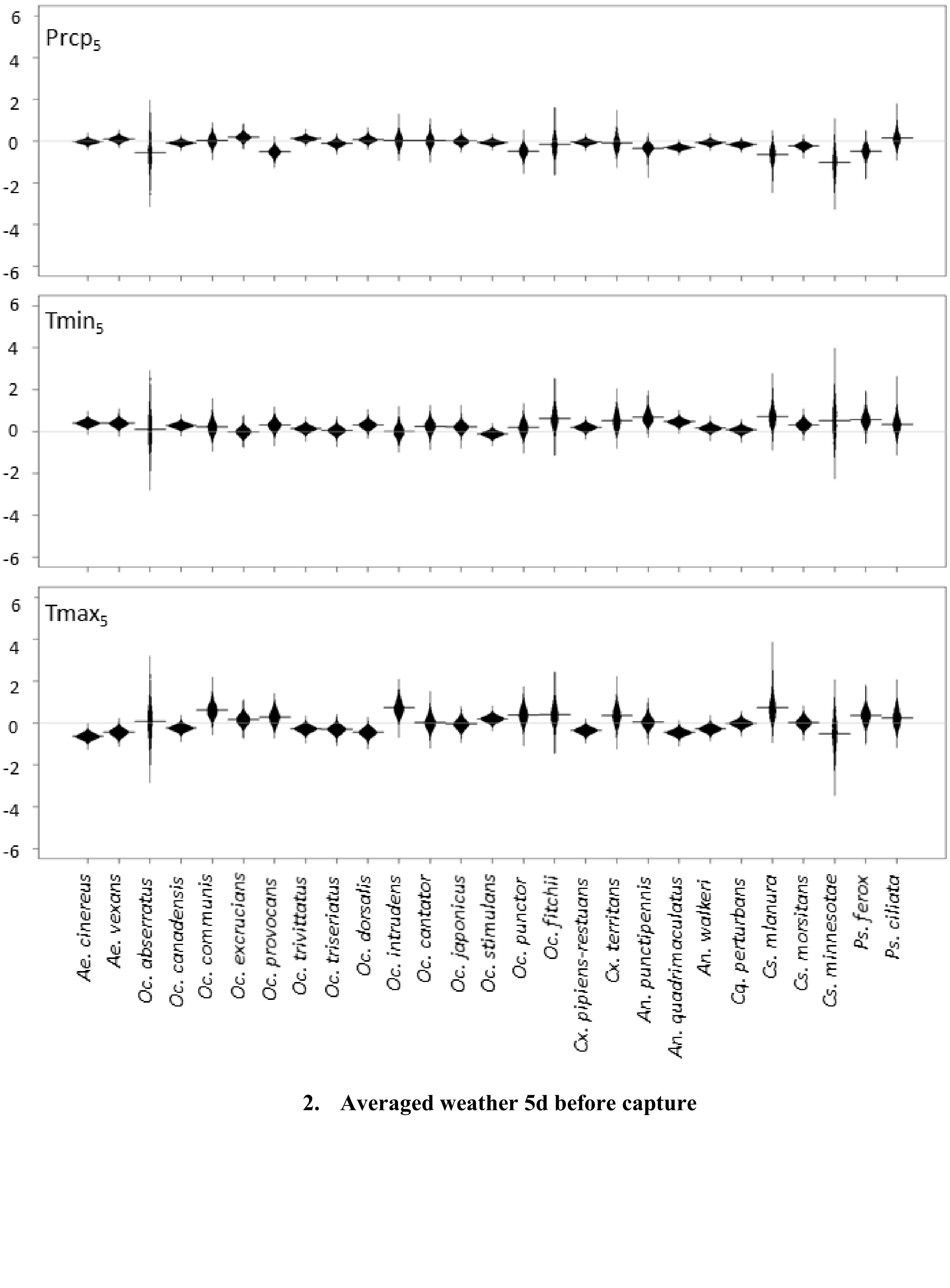


**2. Averaged weather 5d before capture**

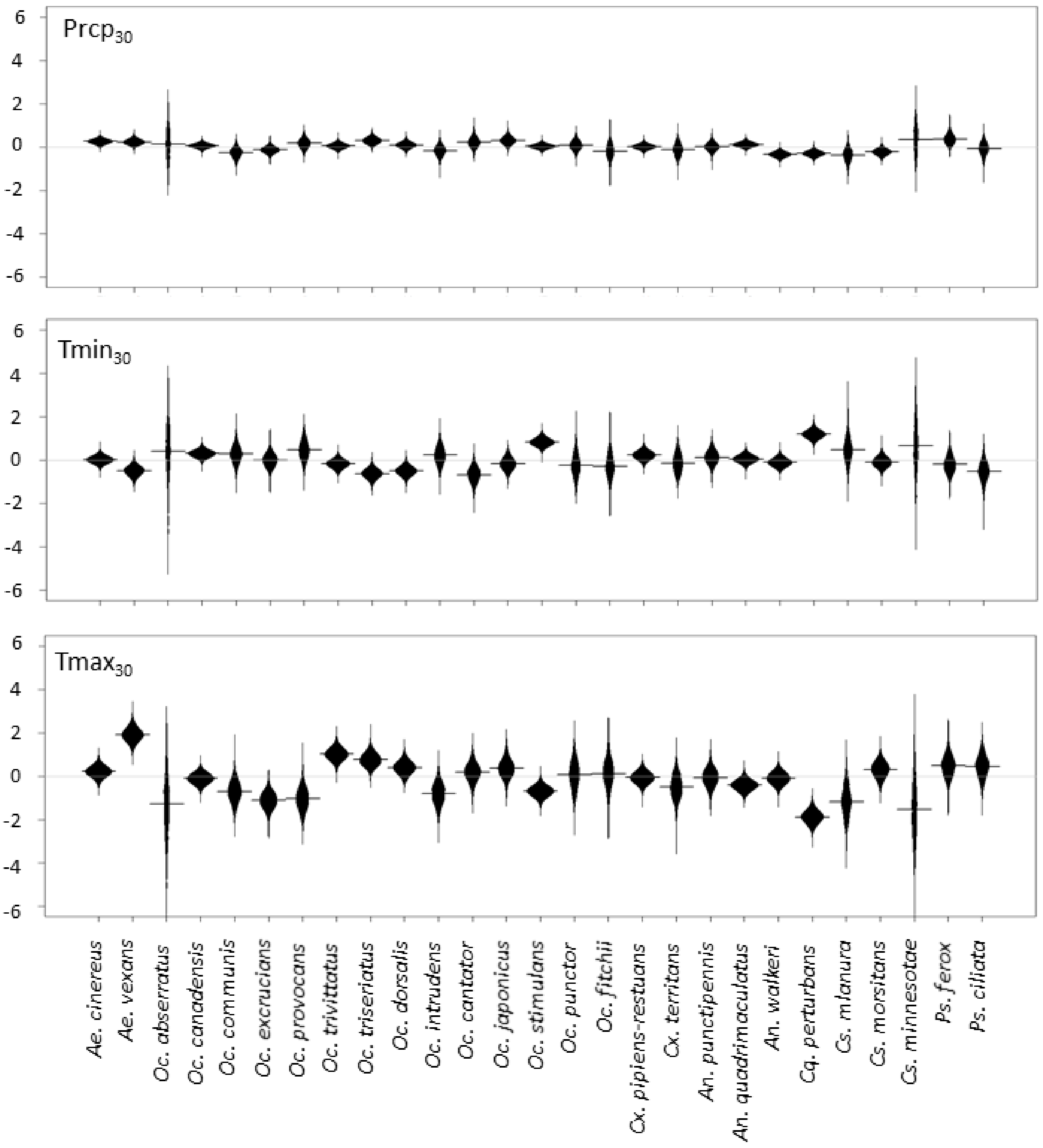


**3. Averaged weather 30d before capture**

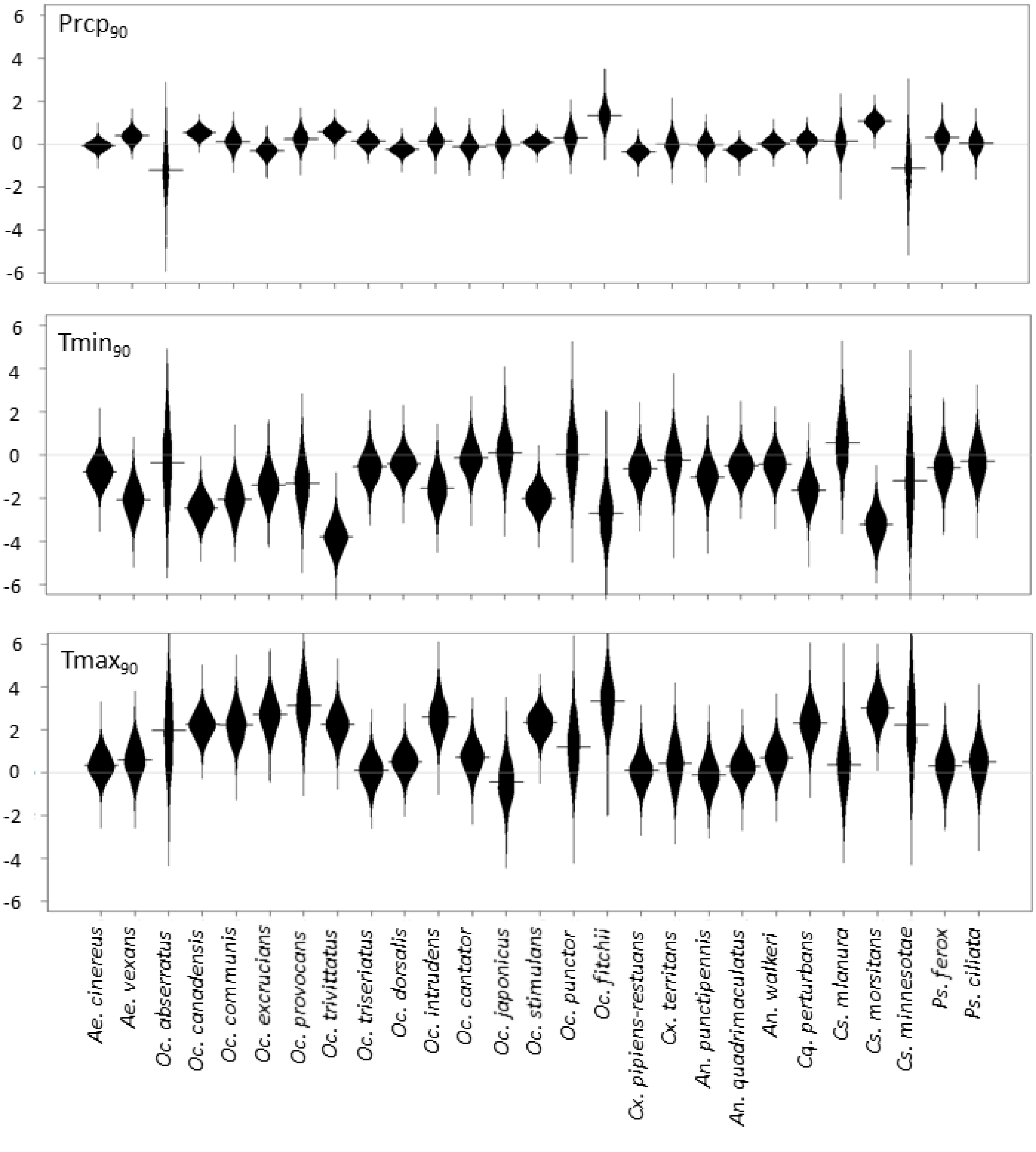


**4. Averaged weather 90d before capture**

**S13 Fig. Violin plots showing the marginal distribution of the weather parameters for each species resulting from the weather-only abundance model. The short segments represent the MCMC iterations for each parameter.**

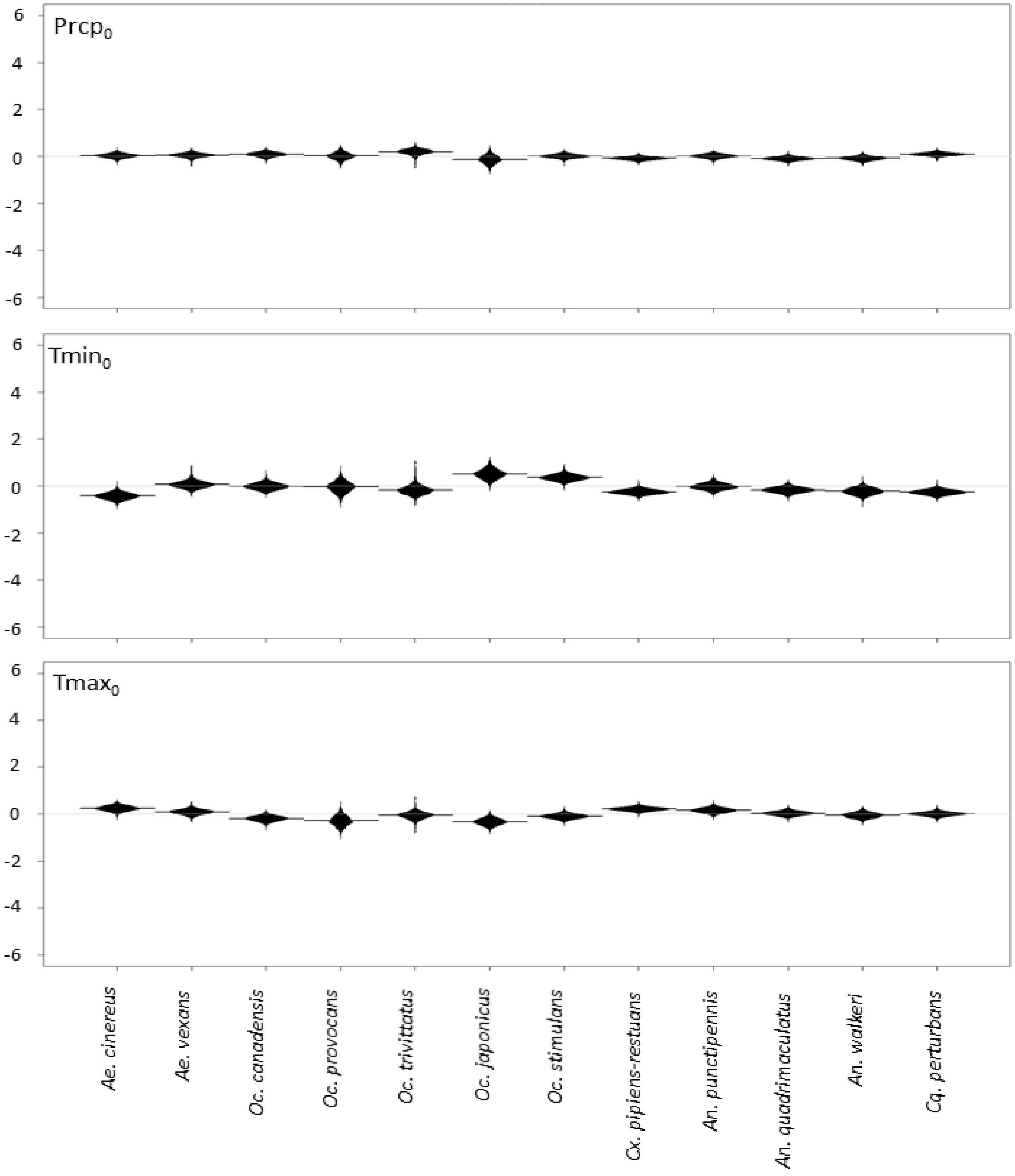


1. **Weather of the day of capture**

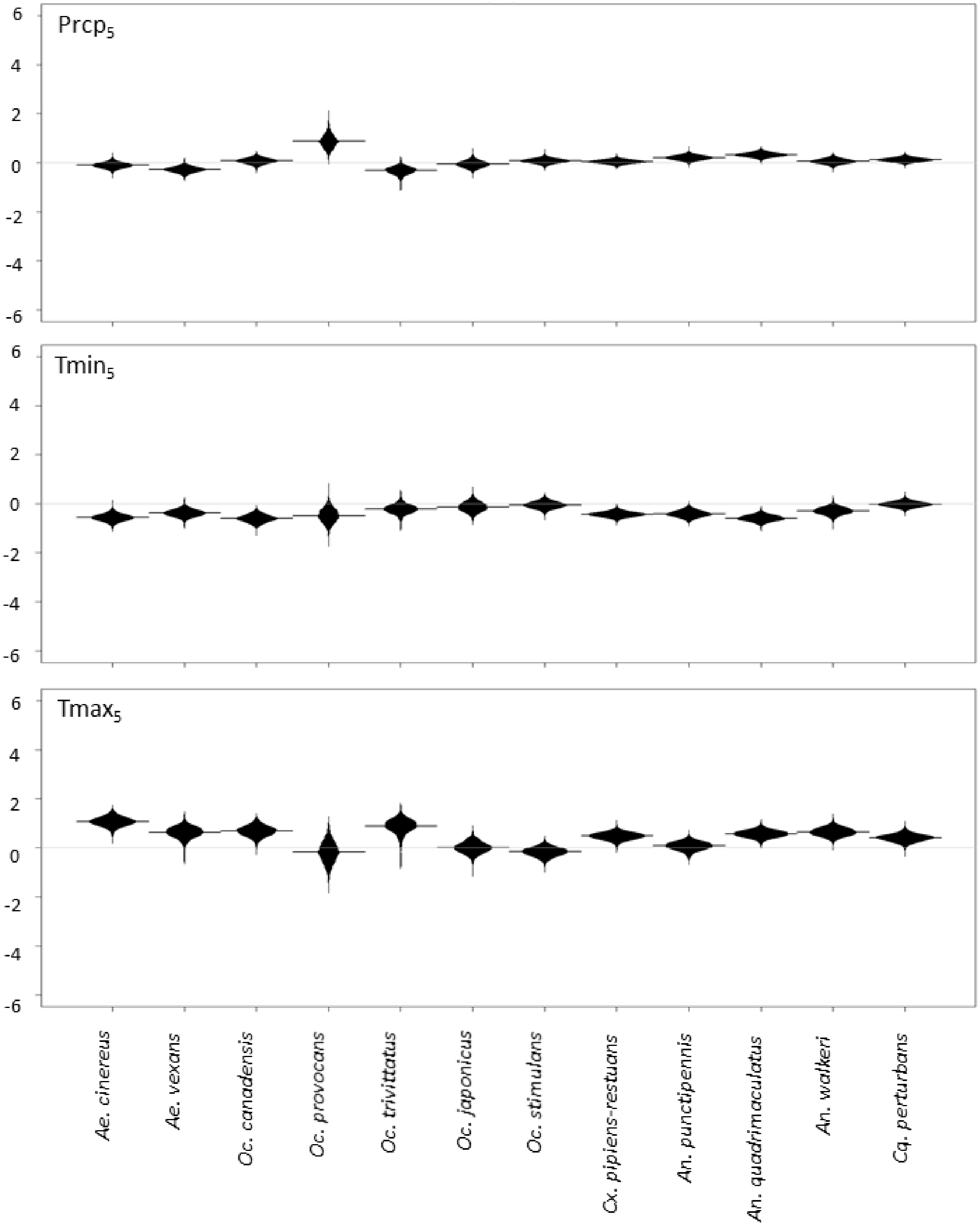


**2. Averaged weather 5 days before capture**

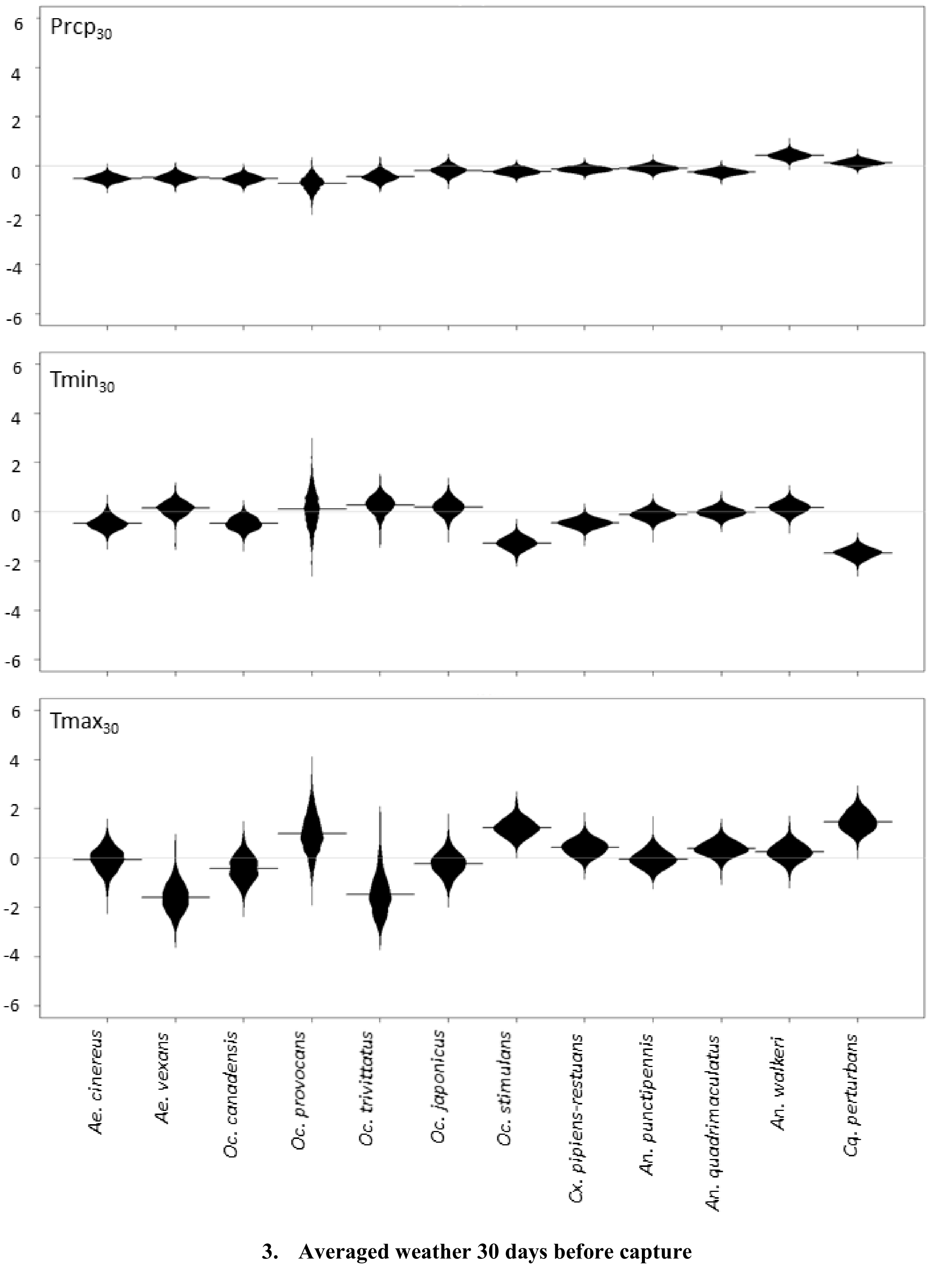


**3. Averaged weather 30 days before capture**

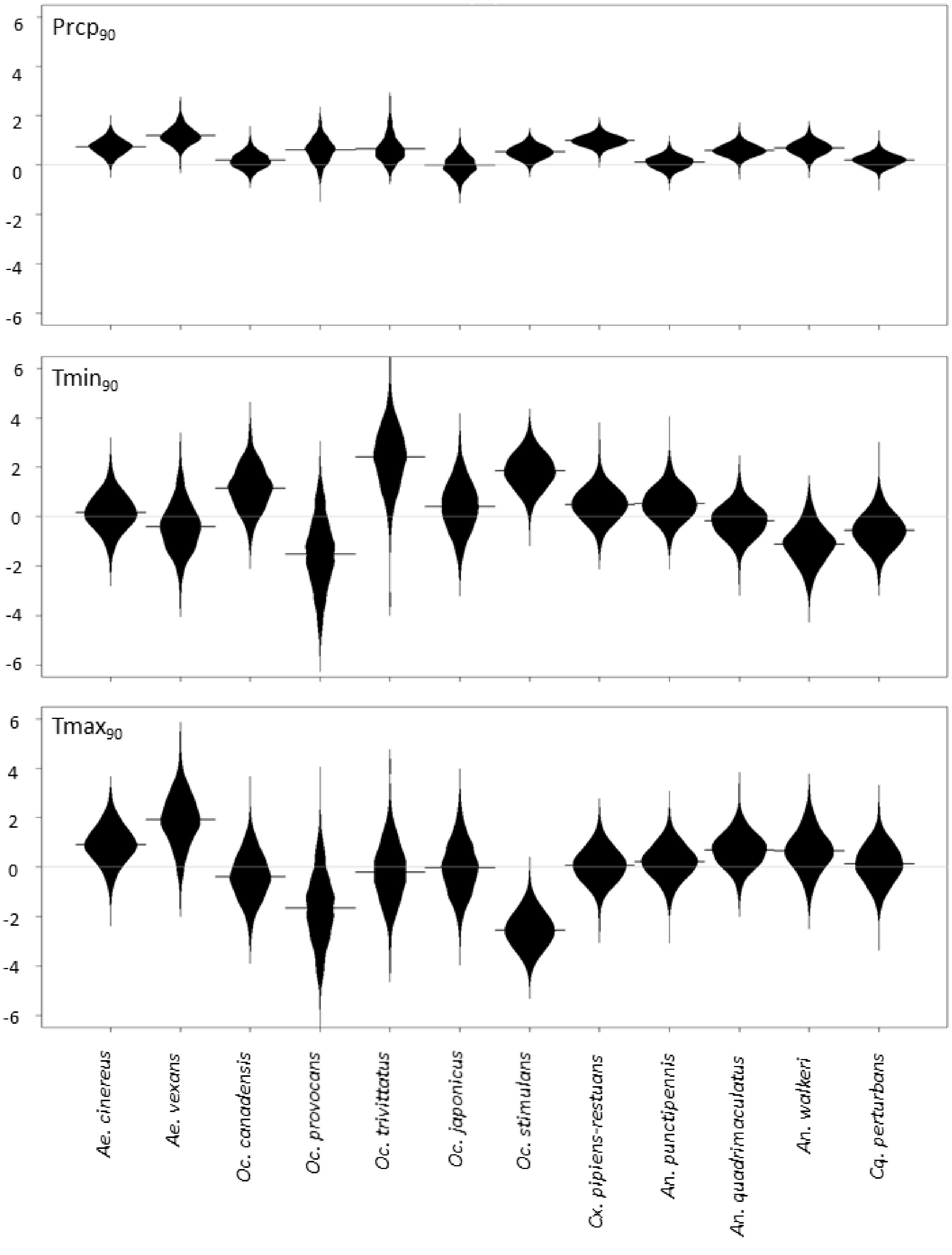


**4. Averaged weather 90 days before capture**

## S14 Table. Root-mean-squared error (RMSE) and are under curve (AUC) for each species

| Species | RMSE (mean = 0.168) | AUC (mean = 0.938) |
|---|---|---|
| *Ae. cinereus* | 0,412 | 0,718 |
| *Ae. vexans* | 0,256 | 0,97 |
| *Anopheles punctipennis* | 0,062 | 1 |
| *Anopheles quadrimaculatus* | 0,329 | 0,898 |
| *Anopheles walkeri* | 0,272 | 0,837 |
| *Coquillettidia perturbans* | 0,404 | 0,836 |
| *Culex pipiens-restuans* | 0,307 | 0,936 |
| *Culex territans* | 0,026 | 0,989 |
| *Culiseta melanura* | 0,052 | 0,998 |
| *Culiseta minnesotae* | 0,032 | 0,999 |
| *Culiseta morsitans* | 0,142 | 0,832 |
| *Ochlerotatus abserratus* | 0,023 | 1 |
| *Ochlerotatus canadensis* | 0,333 | 0,838 |
| *Ochlerotatus cantator* | 0,047 | 0,964 |
| *Ochlerotatus communis* | 0,056 | 0,989 |
| *Ochlerotatus dorsalis* | 0,173 | 0,889 |
| *Ochlerotatus excrucians* | 0,161 | 0,953 |
| *Ochlerotatus fitchii* | 0,024 | 1 |
| *Ochlerotatus intrudens* | 0,059 | 0,988 |
| *Ochlerotatus japonicus* | 0,166 | 0,99 |
| *Ochlerotatus provocans* | 0,163 | 0,966 |
| *Ochlerotatus punctor* | 0,113 | 0,986 |
| *Ochlerotatus stimulans* | 0,357 | 0,884 |
| *Ochlerotatus triseriatus* | 0,196 | 0,935 |
| *Ochlerotatus trivittatus* | 0,3 | 0,951 |
| *Psorophora ciliata* | 0,026 | 0,981 |
| *Psorophora ferox* | 0,051 | 0,994 |

## S15 Fig. ROC curves of the weather-and-land-use model with a probit distribution (occurrence model)

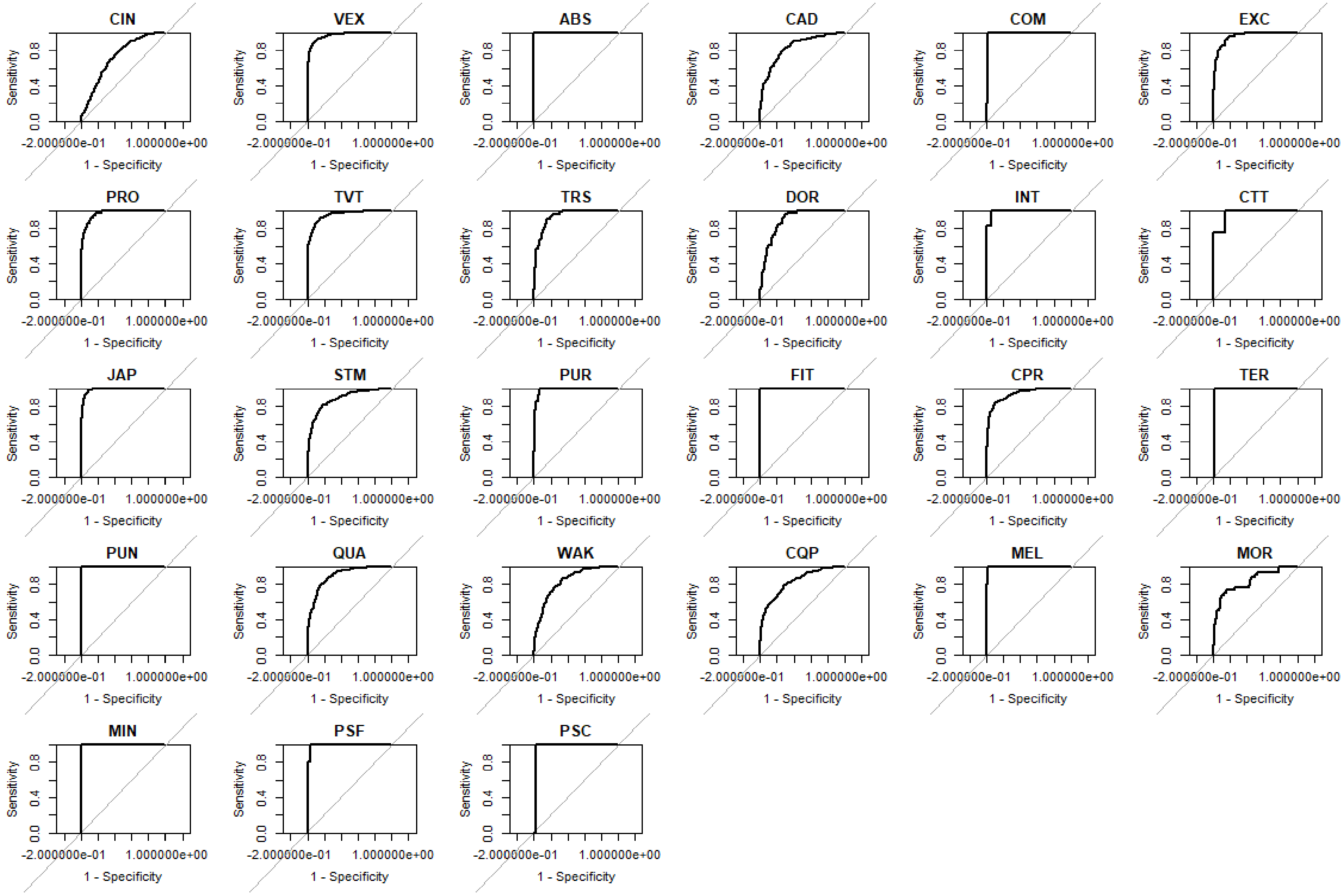

**S16 Table. Root-mean-squared error (RMSE), maximum observed abundance and parameters related to the Pearson's correlation between predicted and observed abundance for each species**

| Species | RMSE (mean = 41.9237) | Maximum observed abundance (mean = 833.25) | Pearson's correlation between predicted and observed abundance | | | | | |
|---|---|---|---|---|---|---|---|---|
| | | | r | 95% CI | | t | df | p-value |
| *Ae. cinereus* | 13.06 | 467 | 0.42 | 0.37 | 0.46 | 17.73 | 1487 | < 2.2E-16 |
| *Ae. vexans* | 183.63 | 3876 | 0.58 | 0.54 | 0.61 | 27.25 | 1487 | < 2.2E-16 |
| *An. punctipennis* | 9.52 | 251 | 0.81 | 0.79 | 0.83 | 53.13 | 1487 | < 2.2E-16 |
| *An. quadrimaculatus* | 3.86 | 81 | 0.70 | 0.67 | 0.73 | 37.95 | 1487 | < 2.2E-16 |
| *An. walkeri* | 1.50 | 20 | 0.60 | 0.57 | 0.63 | 29.16 | 1487 | < 2.2E-16 |
| *Cq. perturbans* | 95.28 | 1550 | 0.45 | 0.40 | 0.49 | 19.21 | 1487 | < 2.2E-16 |
| *Cx. pipiens-restuans* | 69.98 | 1120 | 0.55 | 0.51 | 0.58 | 25.19 | 1487 | < 2.2E-16 |
| *Oc. canadensis* | 15.69 | 380 | 0.49 | 0.45 | 0.53 | 21.72 | 1487 | < 2.2E-16 |
| *Oc. japonicus* | 1.62 | 36 | 0.64 | 0.61 | 0.67 | 32.46 | 1487 | < 2.2E-16 |
| *Oc. provocans* | 1.68 | 42 | 0.44 | 0.39 | 0.48 | 18.65 | 1487 | < 2.2E-16 |
| *Oc. stimulans* | 11.09 | 163 | 0.59 | 0.56 | 0.63 | 28.51 | 1487 | < 2.2E-16 |
| *Oc. trivittatus* | 96.18 | 20421 | 0.87 | 0.86 | 0.88 | 67.50 | 1487 | < 2.2E-16 |

## S17 Fig. Density plots of occurrence – weather-only model

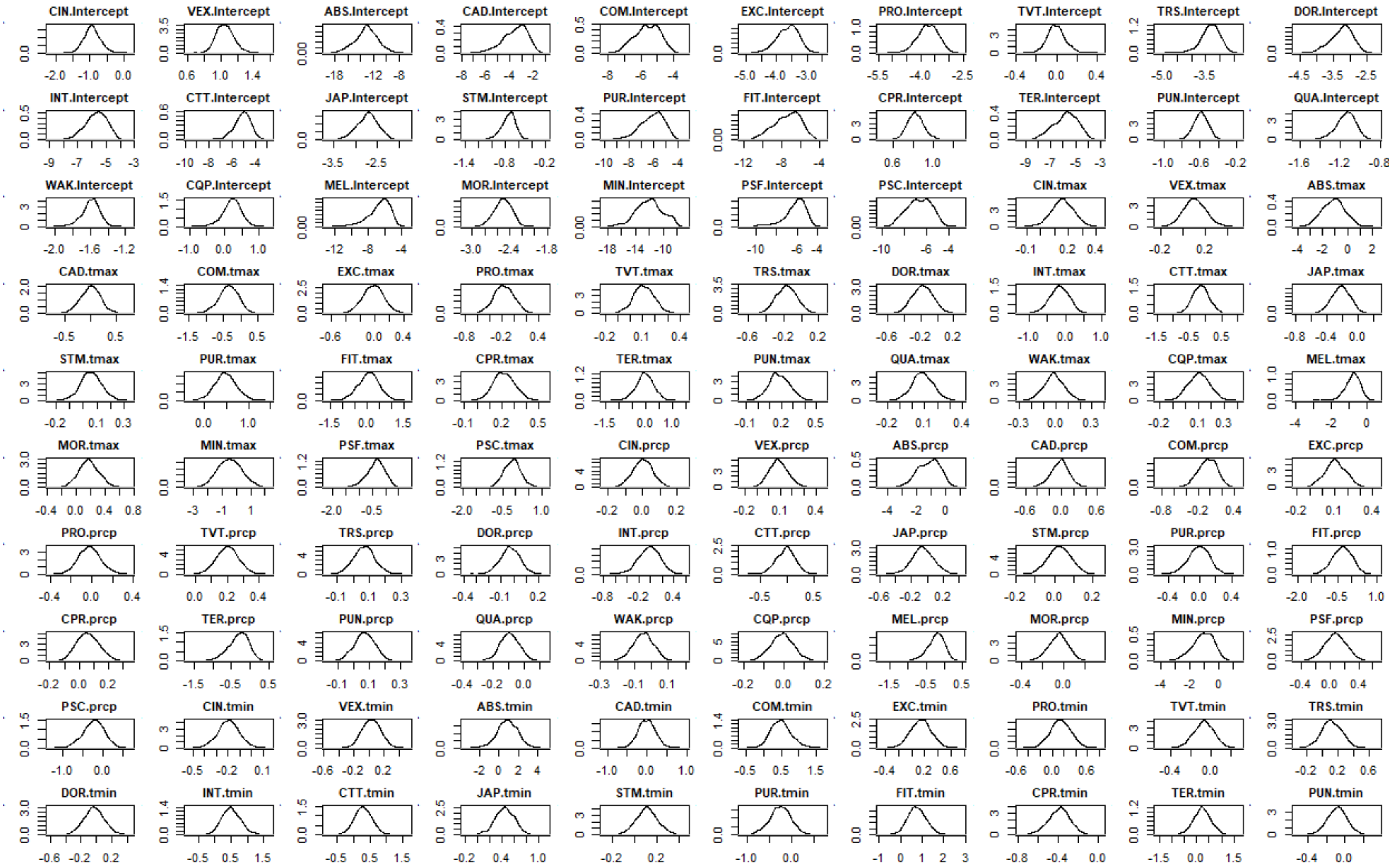

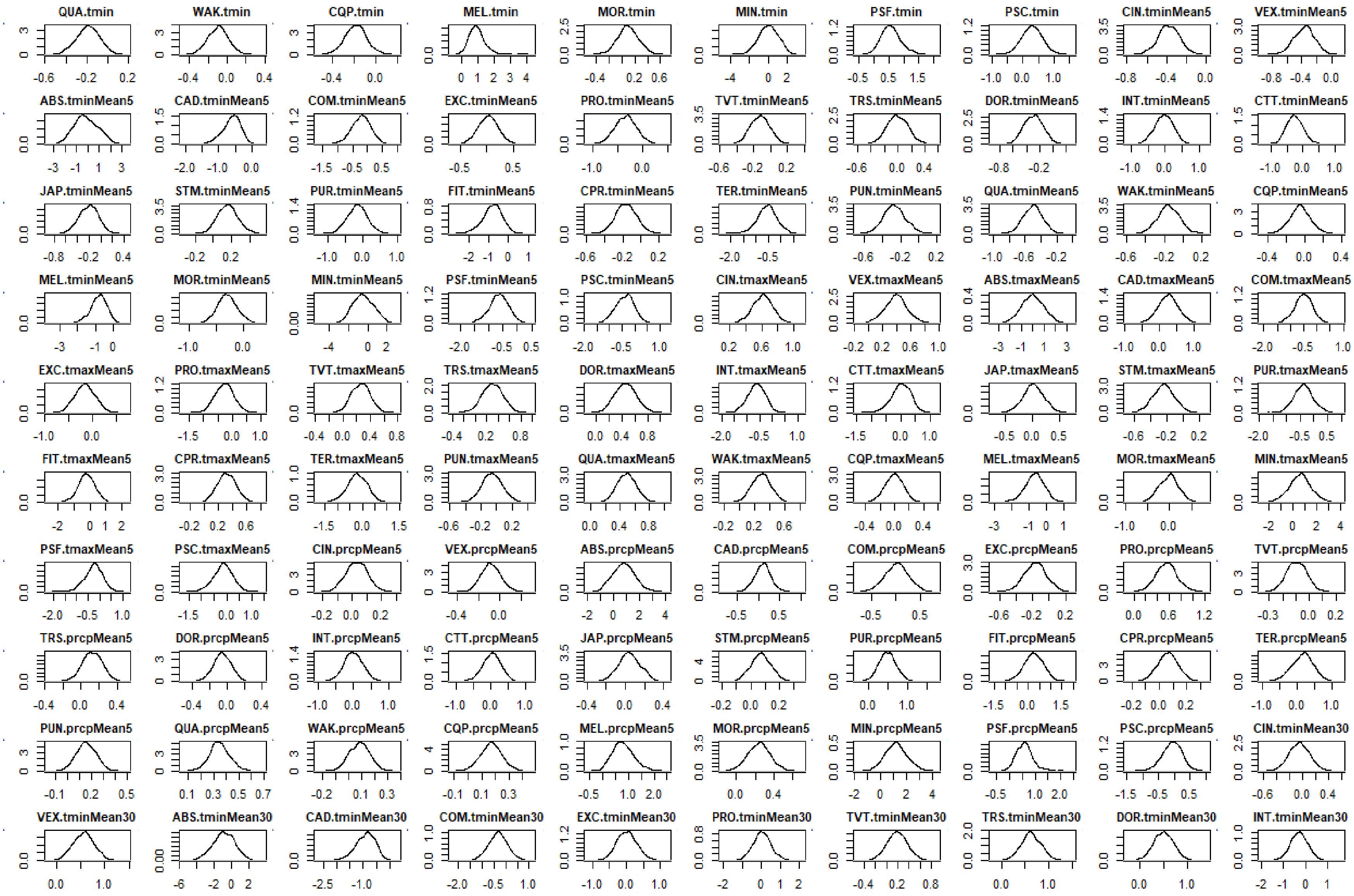
QUA.tmin
WAK.tmin
CQP.tmin
MEL.tmin
MOR.tmin
MIN.tmin
PSF.tmin
PSC.tmin
CIN.tminMean5
VEX.tminMean5
ABS.tminMean5
CAD.tminMean5
COM.tminMean5
EXC.tminMean5
PRO.tminMean5
TVT.tminMean5
TRS.tminMean5
DOR.tminMean5
INT.tminMean5
CTT.tminMean5
JAP.tminMean5
STM.tminMean5
PUR.tminMean5
FIT.tminMean5
CPR.tminMean5
TER.tminMean5
PUN.tminMean5
QUA.tminMean5
WAK.tminMean5
CQP.tminMean5
MEL.tminMean5
MOR.tminMean5
MIN.tminMean5
PSF.tminMean5
PSC.tminMean5
CIN.tmaxMean5
VEX.tmaxMean5
ABS.tmaxMean5
CAD.tmaxMean5
COM.tmaxMean5
EXC.tmaxMean5
PRO.tmaxMean5
TVT.tmaxMean5
TRS.tmaxMean5
DOR.tmaxMean5
INT.tmaxMean5
CTT.tmaxMean5
JAP.tmaxMean5
STM.tmaxMean5
PUR.tmaxMean5
FIT.tmaxMean5
CPR.tmaxMean5
TER.tmaxMean5
PUN.tmaxMean5
QUA.tmaxMean5
WAK.tmaxMean5
CQP.tmaxMean5
MEL.tmaxMean5
MOR.tmaxMean5
MIN.tmaxMean5
PSF.tmaxMean5
PSC.tmaxMean5
CIN.prcpMean5
VEX.prcpMean5
ABS.prcpMean5
CAD.prcpMean5
COM.prcpMean5
EXC.prcpMean5
PRO.prcpMean5
TVT.prcpMean5
TRS.prcpMean5
DOR.prcpMean5
INT.prcpMean5
CTT.prcpMean5
JAP.prcpMean5
STM.prcpMean5
PUR.prcpMean5
FIT.prcpMean5
CPR.prcpMean5
TER.prcpMean5
PUN.prcpMean5
QUA.prcpMean5
WAK.prcpMean5
CQP.prcpMean5
MEL.prcpMean5
MOR.prcpMean5
MIN.prcpMean5
PSF.prcpMean5
PSC.prcpMean5
CIN.tminMean30
VEX.tminMean30
ABS.tminMean30
CAD.tminMean30
COM.tminMean30
EXC.tminMean30
PRO.tminMean30
TVT.tminMean30
TRS.tminMean30
DOR.tminMean30
INT.tminMean30

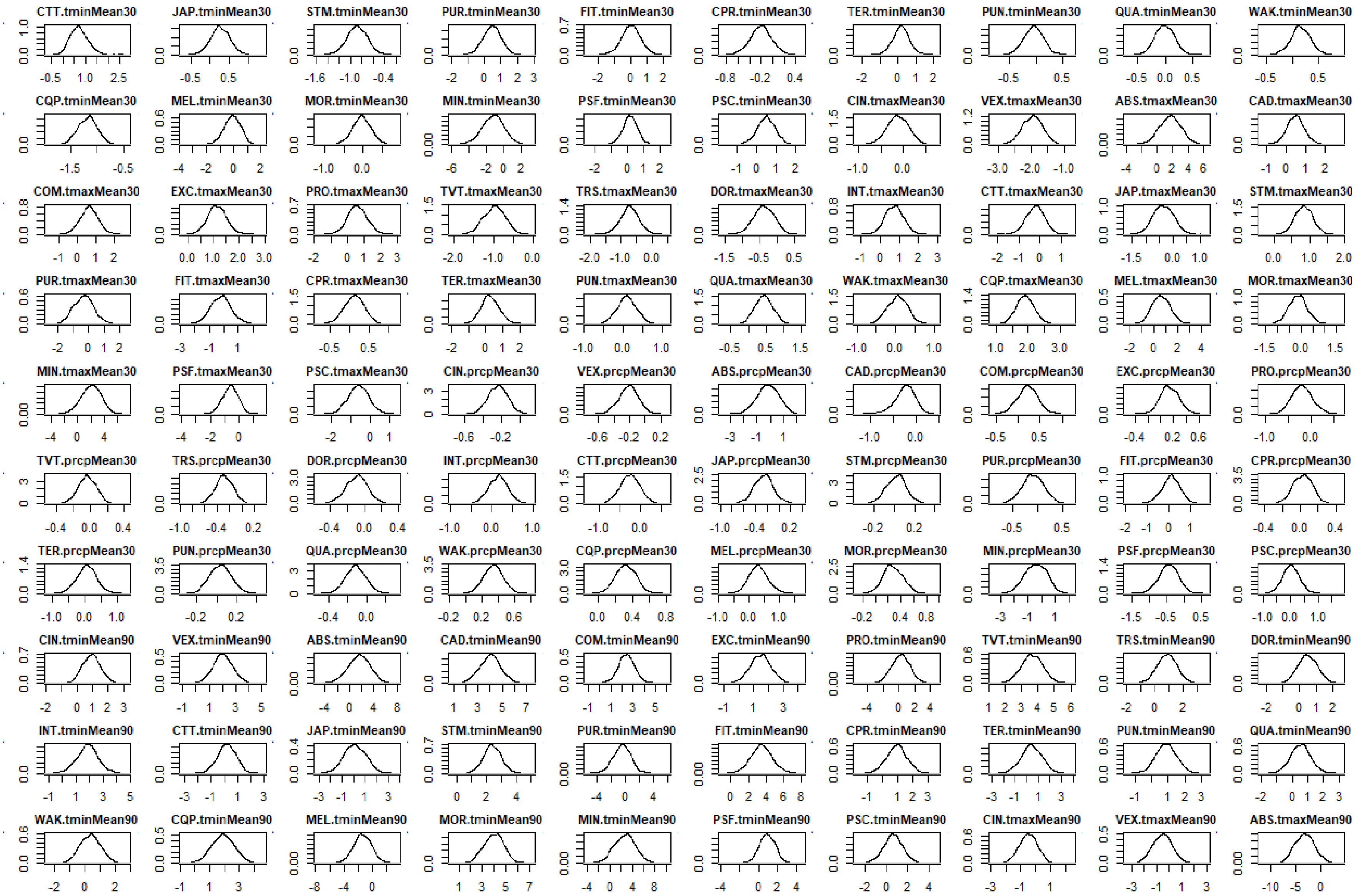
CTT.tminMean30
JAP.tminMean30
STM.tminMean30
PUR.tminMean30
FIT.tminMean30
CPR.tminMean30
TER.tminMean30
PUN.tminMean30
QUA.tminMean30
WAK.tminMean30
CQP.tminMean30
MEL.tminMean30
MOR.tminMean30
MIN.tminMean30
PSF.tminMean30
PSC.tminMean30
CIN.tmaxMean30
VEX.tmaxMean30
ABS.tmaxMean30
CAD.tmaxMean30
COM.tmaxMean30
EXC.tmaxMean30
PRO.tmaxMean30
TVT.tmaxMean30
TRS.tmaxMean30
DOR.tmaxMean30
INT.tmaxMean30
CTT.tmaxMean30
JAP.tmaxMean30
STM.tmaxMean30
PUR.tmaxMean30
FIT.tmaxMean30
CPR.tmaxMean30
TER.tmaxMean30
PUN.tmaxMean30
QUA.tmaxMean30
WAK.tmaxMean30
CQP.tmaxMean30
MEL.tmaxMean30
MOR.tmaxMean30
MIN.tmaxMean30
PSF.tmaxMean30
PSC.tmaxMean30
CIN.prcpMean30
VEX.prcpMean30
ABS.prcpMean30
CAD.prcpMean30
COM.prcpMean30
EXC.prcpMean30
PRO.prcpMean30
TVT.prcpMean30
TRS.prcpMean30
DOR.prcpMean30
INT.prcpMean30
CTT.prcpMean30
JAP.prcpMean30
STM.prcpMean30
PUR.prcpMean30
FIT.prcpMean30
CPR.prcpMean30
TER.prcpMean30
PUN.prcpMean30
QUA.prcpMean30
WAK.prcpMean30
CQP.prcpMean30
MEL.prcpMean30
MOR.prcpMean30
MIN.prcpMean30
PSF.prcpMean30
PSC.prcpMean30
CIN.tminMean90
VEX.tminMean90
ABS.tminMean90
CAD.tminMean90
COM.tminMean90
EXC.tminMean90
PRO.tminMean90
TVT.tminMean90
TRS.tminMean90
DOR.tminMean90
INT.tminMean90
CTT.tminMean90
JAP.tminMean90
STM.tminMean90
PUR.tminMean90
FIT.tminMean90
CPR.tminMean90
TER.tminMean90
PUN.tminMean90
QUA.tminMean90
WAK.tminMean90
CQP.tminMean90
MEL.tminMean90
MOR.tminMean90
MIN.tminMean90
PSF.tminMean90
PSC.tminMean90
CIN.tmaxMean90
VEX.tmaxMean90
ABS.tmaxMean90

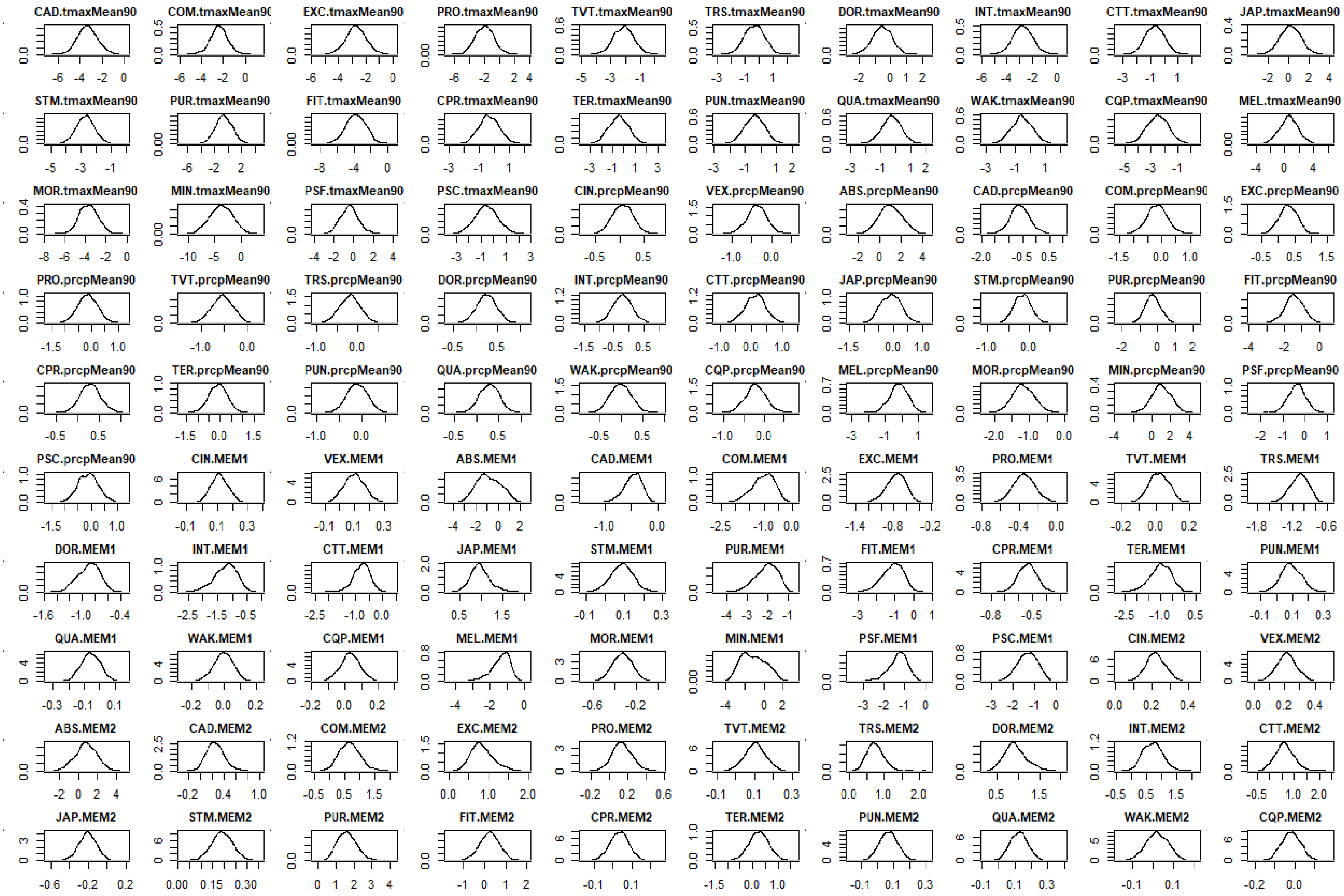
CAD.tmaxMean90
COM.tmaxMean9(
EXC.tmaxMean90
PRO.tmaxMean90
TVT.tmaxMean90
TRS.tmaxMean90
DOR.tmaxMean90
INT.tmaxMean90
CTT.tmaxMean90
JAP.tmaxMean90
STM.tmaxMean90
PUR.tmaxMean90
FIT.tmaxMean90
CPR.tmaxMean90
TER.tmaxMean90
PUN.tmaxMean90
QUA.tmaxMean90
WAK.tmaxMean90
CQP.tmaxMean90
MEL.tmaxMean90
MOR.tmaxMean90
MIN.tmaxMean90
PSF.tmaxMean90
PSC.tmaxMean90
CIN.prcpMean90
VEX.prcpMean90
ABS.prcpMean90
CAD.prcpMean90
COM.prcpMean90
EXC.prcpMean90
PRO.prcpMean90
TVT.prcpMean90
TRS.prcpMean90
DOR.prcpMean90
INT.prcpMean90
CTT.prcpMean90
JAP.prcpMean90
STM.prcpMean90
PUR.prcpMean90
FIT.prcpMean90
CPR.prcpMean90
TER.prcpMean90
PUN.prcpMean90
QUA.prcpMean90
WAK.prcpMean90
CQP.prcpMean90
MEL.prcpMean90
MOR.prcpMean90
MIN.prcpMean90
PSF.prcpMean90
PSC.prcpMean90
CIN.MEM1
VEX.MEM1
ABS.MEM1
CAD.MEM1
COM.MEM1
EXC.MEM1
PRO.MEM1
TVT.MEM1
TRS.MEM1
DOR.MEM1
INT.MEM1
CTT.MEM1
JAP.MEM1
STM.MEM1
PUR.MEM1
FIT.MEM1
CPR.MEM1
TER.MEM1
PUN.MEM1
QUA.MEM1
WAK.MEM1
CQP.MEM1
MEL.MEM1
MOR.MEM1
MIN.MEM1
PSF.MEM1
PSC.MEM1
CIN.MEM2
VEX.MEM2
ABS.MEM2
CAD.MEM2
COM.MEM2
EXC.MEM2
PRO.MEM2
TVT.MEM2
TRS.MEM2
DOR.MEM2
INT.MEM2
CTT.MEM2
JAP.MEM2
STM.MEM2
PUR.MEM2
FIT.MEM2
CPR.MEM2
TER.MEM2
PUN.MEM2
QUA.MEM2
WAK.MEM2
CQP.MEM2

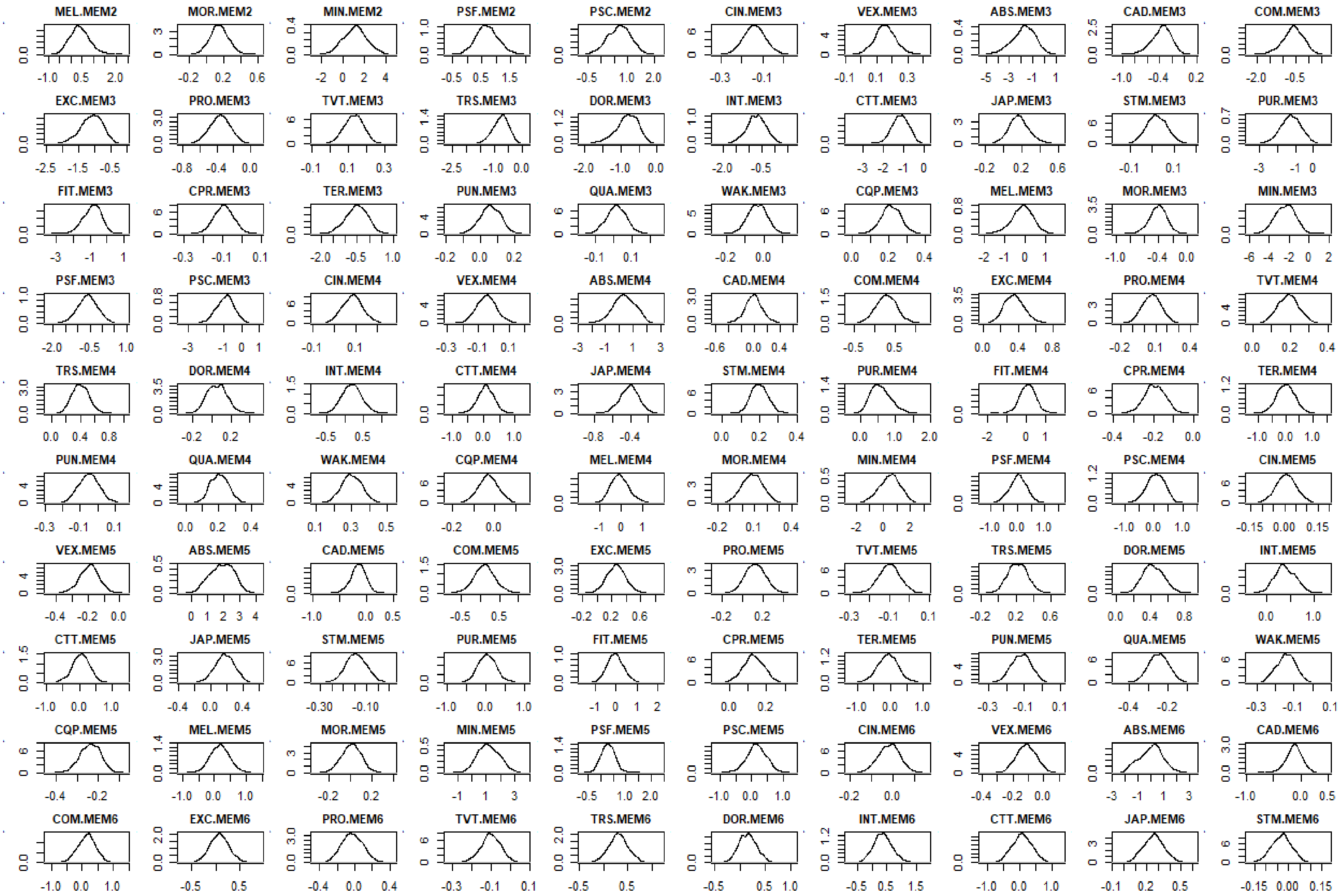

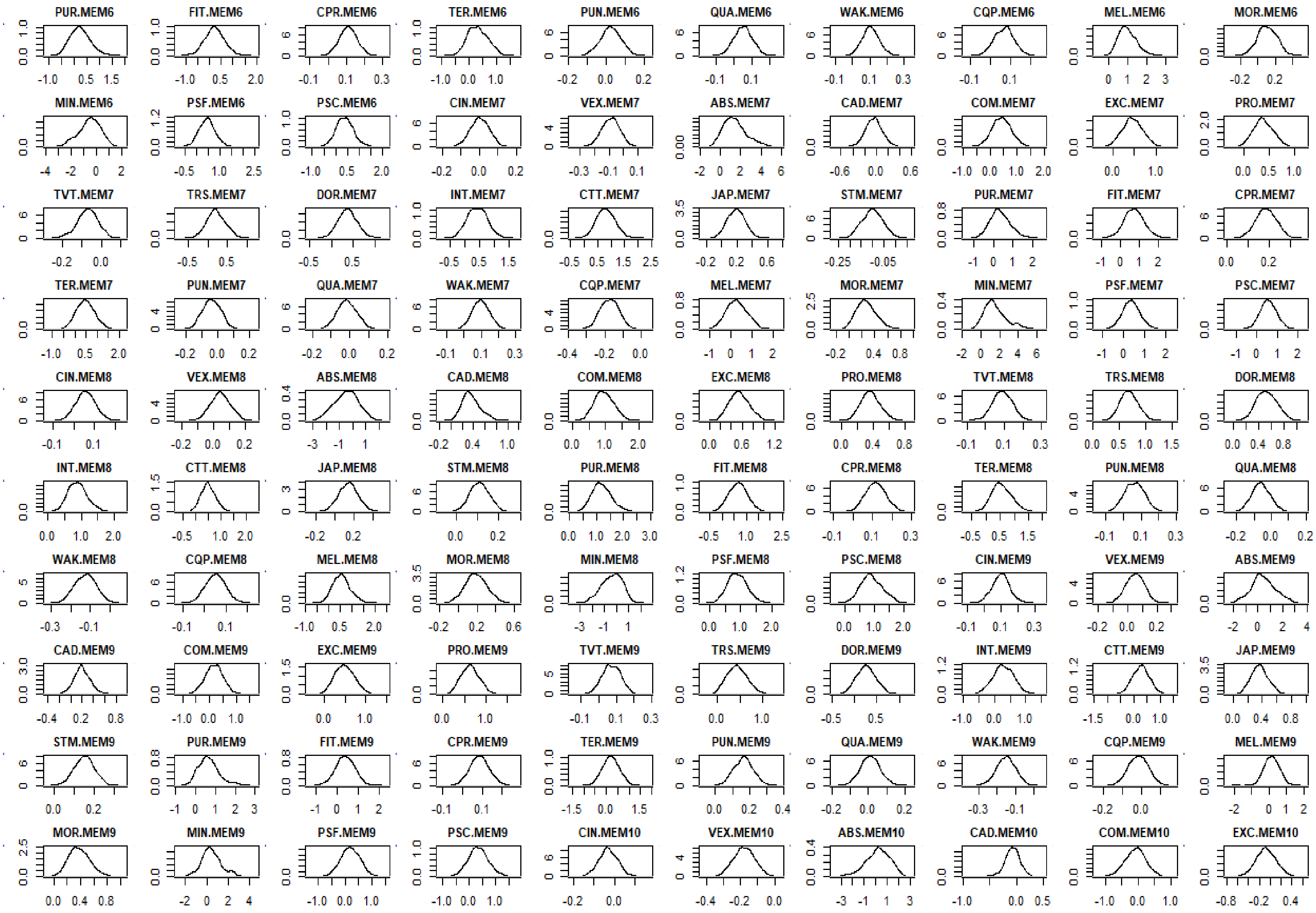
PUR.MEM6
FIT.MEM6
CPR.MEM6
TER.MEM6
PUN.MEM6
QUA.MEM6
WAK.MEM6
CQP.MEM6
MEL.MEM6
MOR.MEM6
MIN.MEM6
PSF.MEM6
PSC.MEM6
CIN.MEM7
VEX.MEM7
ABS.MEM7
CAD.MEM7
COM.MEM7
EXC.MEM7
PRO.MEM7
TVT.MEM7
TRS.MEM7
DOR.MEM7
INT.MEM7
CTT.MEM7
JAP.MEM7
STM.MEM7
PUR.MEM7
FIT.MEM7
CPR.MEM7
TER.MEM7
PUN.MEM7
QUA.MEM7
WAK.MEM7
CQP.MEM7
MEL.MEM7
MOR.MEM7
MIN.MEM7
PSF.MEM7
PSC.MEM7
CIN.MEM8
VEX.MEM8
ABS.MEM8
CAD.MEM8
COM.MEM8
EXC.MEM8
PRO.MEM8
TVT.MEM8
TRS.MEM8
DOR.MEM8
INT.MEM8
CTT.MEM8
JAP.MEM8
STM.MEM8
PUR.MEM8
FIT.MEM8
CPR.MEM8
TER.MEM8
PUN.MEM8
QUA.MEM8
WAK.MEM8
CQP.MEM8
MEL.MEM8
MOR.MEM8
MIN.MEM8
PSF.MEM8
PSC.MEM8
CIN.MEM9
VEX.MEM9
ABS.MEM9
CAD.MEM9
COM.MEM9
EXC.MEM9
PRO.MEM9
TVT.MEM9
TRS.MEM9
DOR.MEM9
INT.MEM9
CTT.MEM9
JAP.MEM9
STM.MEM9
PUR.MEM9
FIT.MEM9
CPR.MEM9
TER.MEM9
PUN.MEM9
QUA.MEM9
WAK.MEM9
CQP.MEM9
MEL.MEM9
MOR.MEM9
MIN.MEM9
PSF.MEM9
PSC.MEM9
CIN.MEM10
VEX.MEM10
ABS.MEM10
CAD.MEM10
COM.MEM10
EXC.MEM10

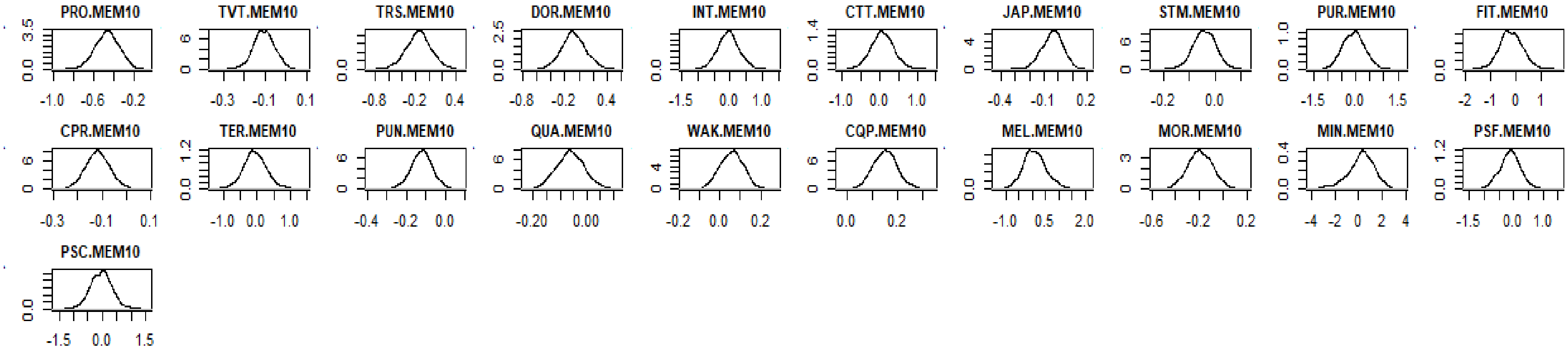
PRO.MEM10
TVT.MEM10
TRS.MEM10
DOR.MEM10
INT.MEM10
CTT.MEM10
JAP.MEM10
STM.MEM10
PUR.MEM10
FIT.MEM10
CPR.MEM10
TER.MEM10
PUN.MEM10
QUA.MEM10
WAK.MEM10
CQP.MEM10
MEL.MEM10
MOR.MEM10
MIN.MEM10
PSF.MEM10
PSC.MEM10

# S18 Fig. Density plots of occurrence – weather-and-land-use model

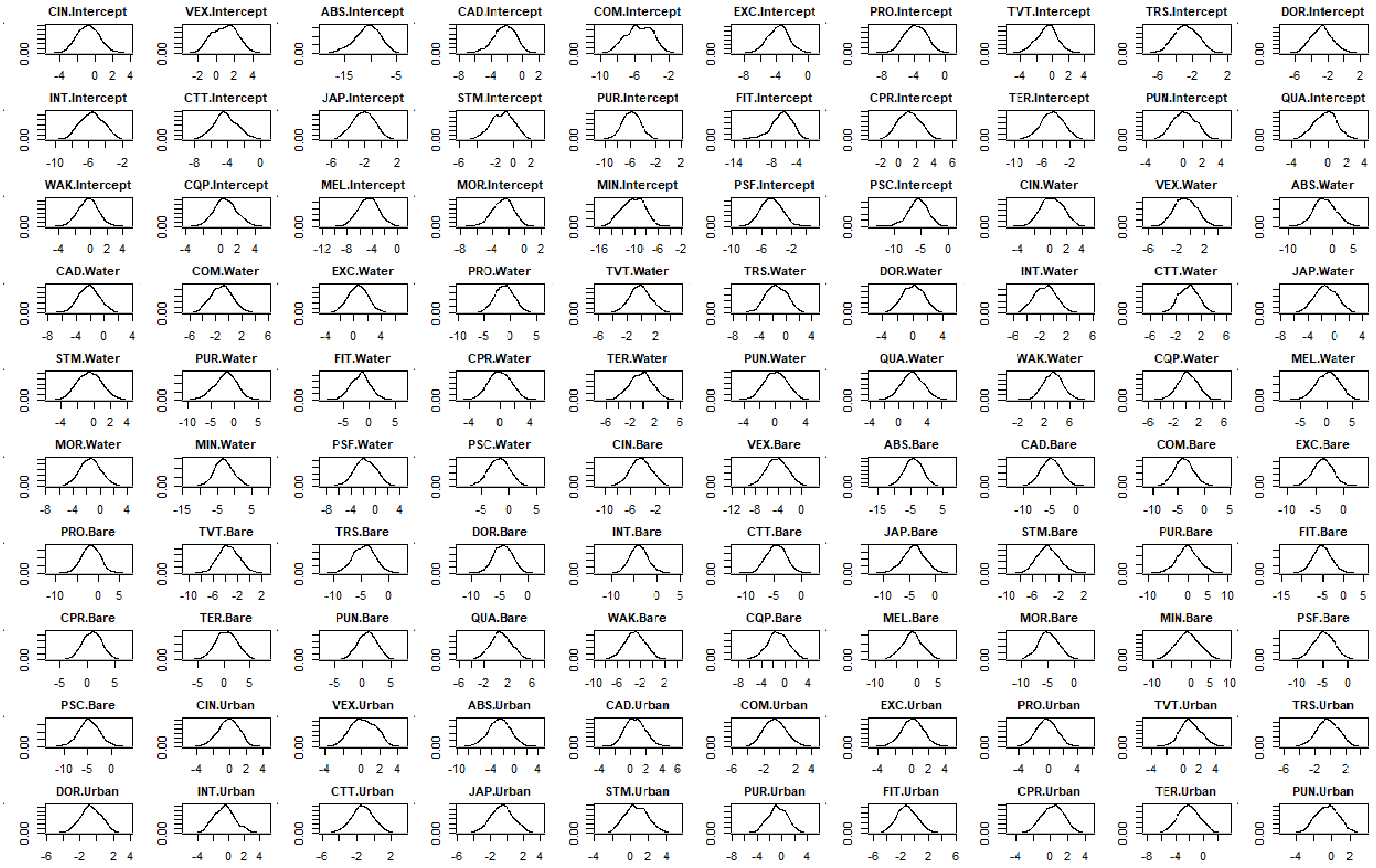

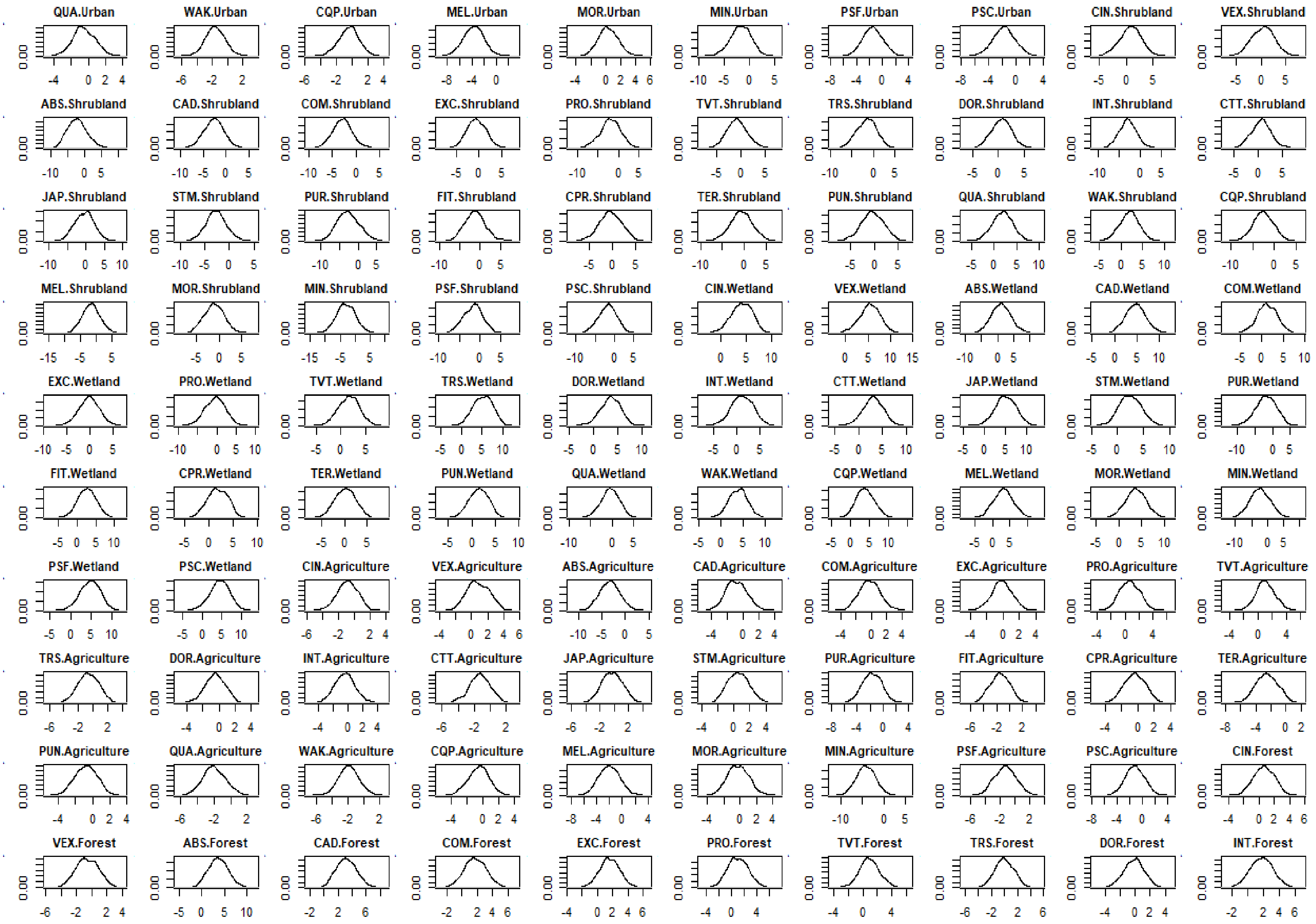
QUA.Urban
WAK.Urban
CQP.Urban
MEL.Urban
MOR.Urban
MIN.Urban
PSF.Urban
PSC.Urban
CIN.Shrubland
VEX.Shrubland
ABS.Shrubland
CAD.Shrubland
COM.Shrubland
EXC.Shrubland
PRO.Shrubland
TVT.Shrubland
TRS.Shrubland
DOR.Shrubland
INT.Shrubland
CTT.Shrubland
JAP.Shrubland
STM.Shrubland
PUR.Shrubland
FIT.Shrubland
CPR.Shrubland
TER.Shrubland
PUN.Shrubland
QUA.Shrubland
WAK.Shrubland
CQP.Shrubland
MEL.Shrubland
MOR.Shrubland
MIN.Shrubland
PSF.Shrubland
PSC.Shrubland
CIN.Wetland
VEX.Wetland
ABS.Wetland
CAD.Wetland
COM.Wetland
EXC.Wetland
PRO.Wetland
TVT.Wetland
TRS.Wetland
DOR.Wetland
INT.Wetland
CTT.Wetland
JAP.Wetland
STM.Wetland
PUR.Wetland
FIT.Wetland
CPR.Wetland
TER.Wetland
PUN.Wetland
QUA.Wetland
WAK.Wetland
CQP.Wetland
MEL.Wetland
MOR.Wetland
MIN.Wetland
PSF.Wetland
PSC.Wetland
CIN.Agriculture
VEX.Agriculture
ABS.Agriculture
CAD.Agriculture
COM.Agriculture
EXC.Agriculture
PRO.Agriculture
TVT.Agriculture
TRS.Agriculture
DOR.Agriculture
INT.Agriculture
CTT.Agriculture
JAP.Agriculture
STM.Agriculture
PUR.Agriculture
FIT.Agriculture
CPR.Agriculture
TER.Agriculture
PUN.Agriculture
QUA.Agriculture
WAK.Agriculture
CQP.Agriculture
MEL.Agriculture
MOR.Agriculture
MIN.Agriculture
PSF.Agriculture
PSC.Agriculture
CIN.Forest
VEX.Forest
ABS.Forest
CAD.Forest
COM.Forest
EXC.Forest
PRO.Forest
TVT.Forest
TRS.Forest
DOR.Forest
INT.Forest

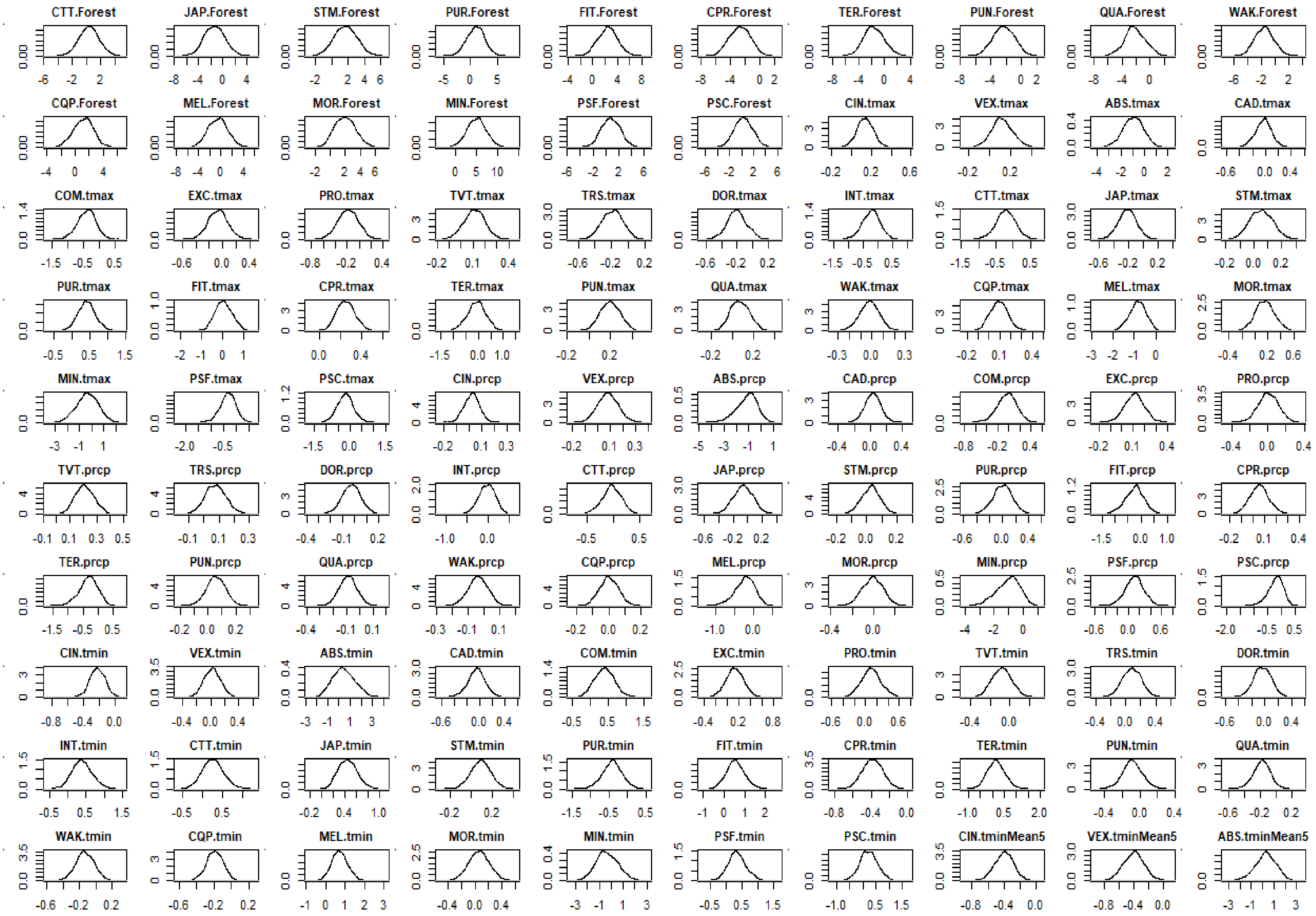
CTT.Forest
JAP.Forest
STM.Forest
PUR.Forest
FIT.Forest
CPR.Forest
TER.Forest
PUN.Forest
QUA.Forest
WAK.Forest
CQP.Forest
MEL.Forest
MOR.Forest
MIN.Forest
PSF.Forest
PSC.Forest
CIN.tmax
VEX.tmax
ABS.tmax
CAD.tmax
COM.tmax
EXC.tmax
PRO.tmax
TVT.tmax
TRS.tmax
DOR.tmax
INT.tmax
CTT.tmax
JAP.tmax
STM.tmax
PUR.tmax
FIT.tmax
CPR.tmax
TER.tmax
PUN.tmax
QUA.tmax
WAK.tmax
CQP.tmax
MEL.tmax
MOR.tmax
MIN.tmax
PSF.tmax
PSC.tmax
CIN.prcp
VEX.prcp
ABS.prcp
CAD.prcp
COM.prcp
EXC.prcp
PRO.prcp
TVT.prcp
TRS.prcp
DOR.prcp
INT.prcp
CTT.prcp
JAP.prcp
STM.prcp
PUR.prcp
FIT.prcp
CPR.prcp
TER.prcp
PUN.prcp
QUA.prcp
WAK.prcp
CQP.prcp
MEL.prcp
MOR.prcp
MIN.prcp
PSF.prcp
PSC.prcp
CIN.tmin
VEX.tmin
ABS.tmin
CAD.tmin
COM.tmin
EXC.tmin
PRO.tmin
TVT.tmin
TRS.tmin
DOR.tmin
INT.tmin
CTT.tmin
JAP.tmin
STM.tmin
PUR.tmin
FIT.tmin
CPR.tmin
TER.tmin
PUN.tmin
QUA.tmin
WAK.tmin
CQP.tmin
MEL.tmin
MOR.tmin
MIN.tmin
PSF.tmin
PSC.tmin
CIN.tminMean5
VEX.tminMean5
ABS.tminMean5

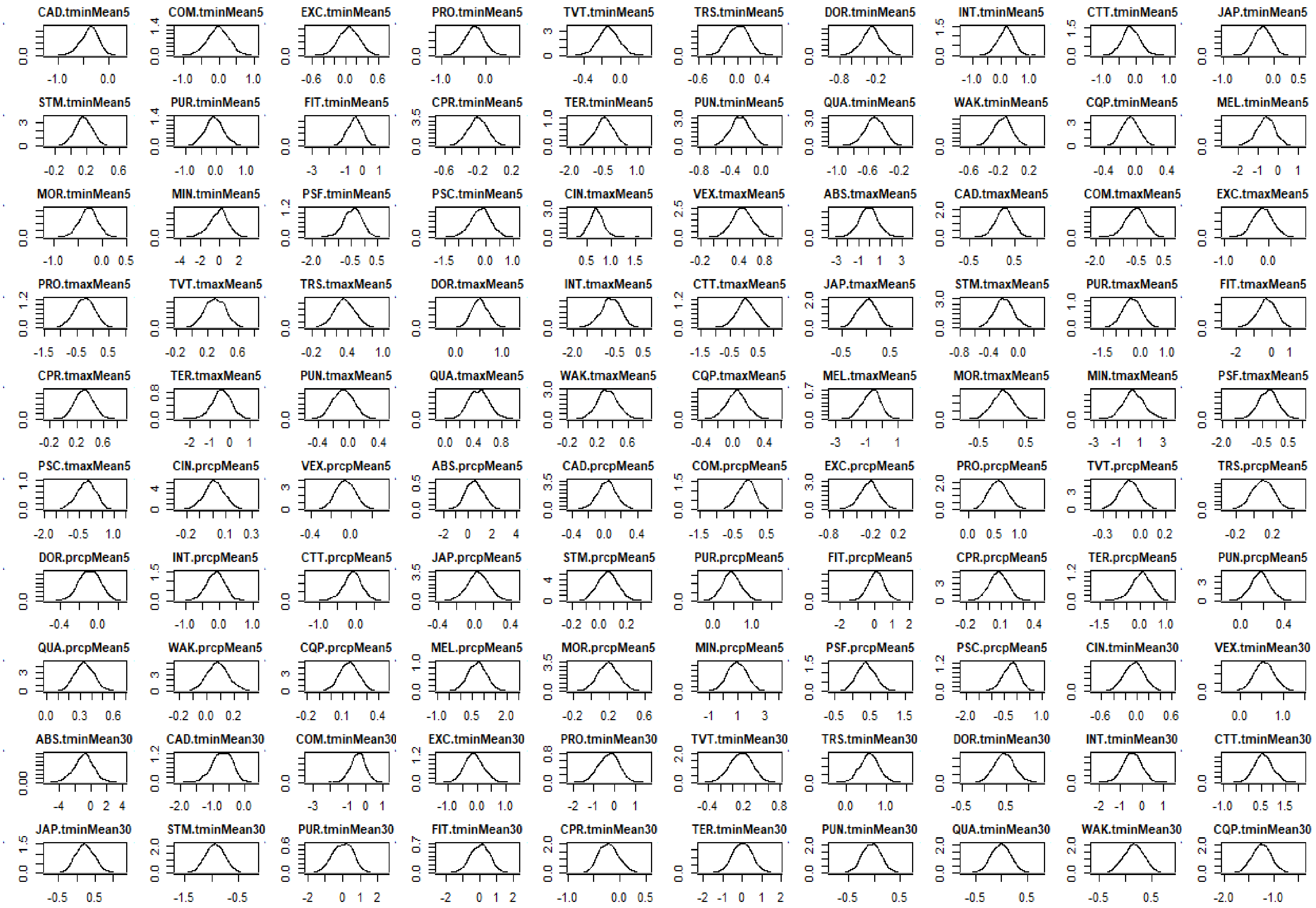
CAD.tminMean5
COM.tminMean5
EXC.tminMean5
PRO.tminMean5
TVT.tminMean5
TRS.tminMean5
DOR.tminMean5
INT.tminMean5
CTT.tminMean5
JAP.tminMean5
STM.tminMean5
PUR.tminMean5
FIT.tminMean5
CPR.tminMean5
TER.tminMean5
PUN.tminMean5
QUA.tminMean5
WAK.tminMean5
CQP.tminMean5
MEL.tminMean5
MOR.tminMean5
MIN.tminMean5
PSF.tminMean5
PSC.tminMean5
CIN.tmaxMean5
VEX.tmaxMean5
ABS.tmaxMean5
CAD.tmaxMean5
COM.tmaxMean5
EXC.tmaxMean5
PRO.tmaxMean5
TVT.tmaxMean5
TRS.tmaxMean5
DOR.tmaxMean5
INT.tmaxMean5
CTT.tmaxMean5
JAP.tmaxMean5
STM.tmaxMean5
PUR.tmaxMean5
FIT.tmaxMean5
CPR.tmaxMean5
TER.tmaxMean5
PUN.tmaxMean5
QUA.tmaxMean5
WAK.tmaxMean5
CQP.tmaxMean5
MEL.tmaxMean5
MOR.tmaxMean5
MIN.tmaxMean5
PSF.tmaxMean5
PSC.tmaxMean5
CIN.prcpMean5
VEX.prcpMean5
ABS.prcpMean5
CAD.prcpMean5
COM.prcpMean5
EXC.prcpMean5
PRO.prcpMean5
TVT.prcpMean5
TRS.prcpMean5
DOR.prcpMean5
INT.prcpMean5
CTT.prcpMean5
JAP.prcpMean5
STM.prcpMean5
PUR.prcpMean5
FIT.prcpMean5
CPR.prcpMean5
TER.prcpMean5
PUN.prcpMean5
QUA.prcpMean5
WAK.prcpMean5
CQP.prcpMean5
MEL.prcpMean5
MOR.prcpMean5
MIN.prcpMean5
PSF.prcpMean5
PSC.prcpMean5
CIN.tminMean30
VEX.tminMean30
ABS.tminMean30
CAD.tminMean30
COM.tminMean30
EXC.tminMean30
PRO.tminMean30
TVT.tminMean30
TRS.tminMean30
DOR.tminMean30
INT.tminMean30
CTT.tminMean30
JAP.tminMean30
STM.tminMean30
PUR.tminMean30
FIT.tminMean30
CPR.tminMean30
TER.tminMean30
PUN.tminMean30
QUA.tminMean30
WAK.tminMean30
CQP.tminMean30

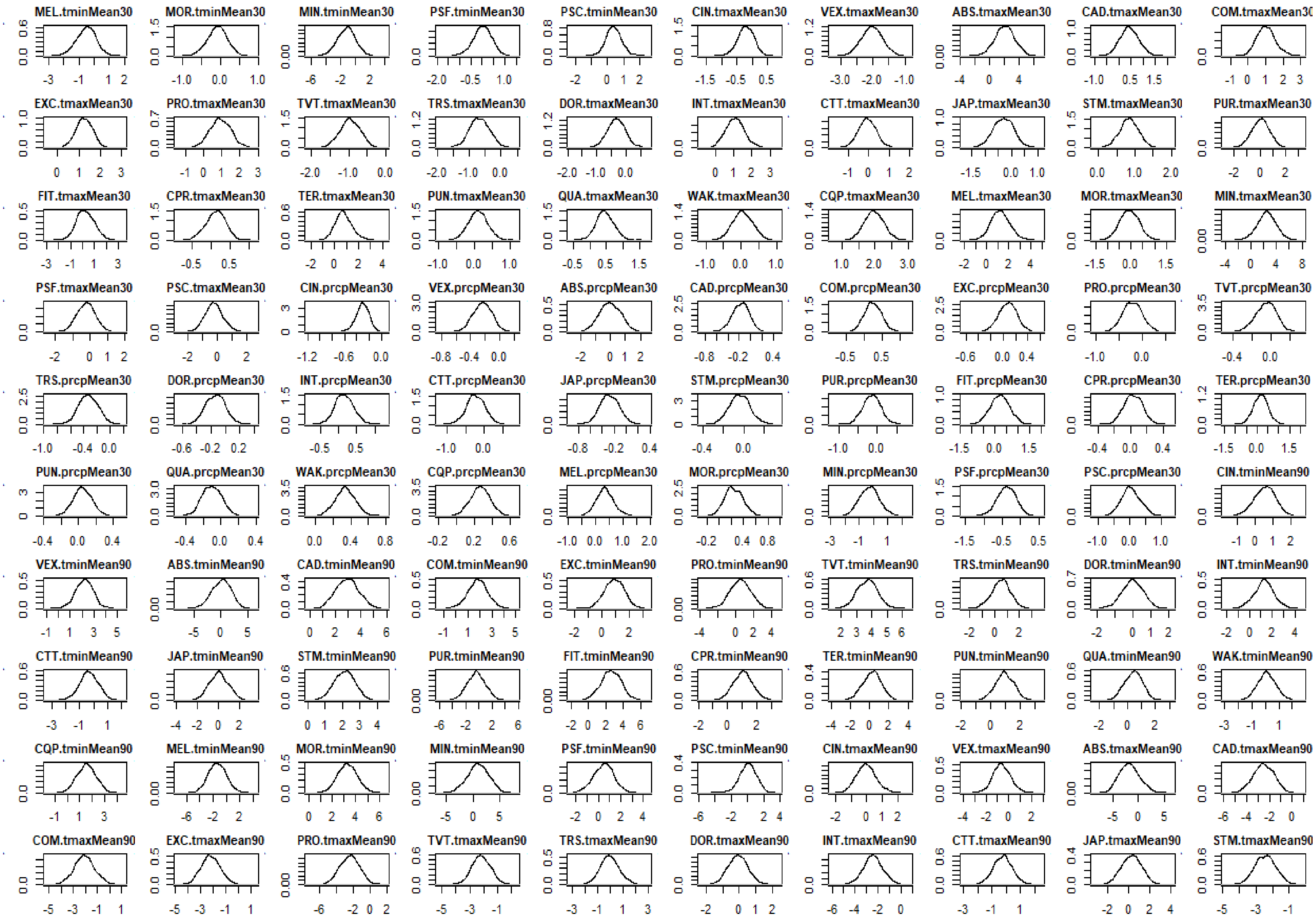
MEL.tminMean30
MOR.tminMean30
MIN.tminMean30
PSF.tminMean30
PSC.tminMean30
CIN.tmaxMean30
VEX.tmaxMean30
ABS.tmaxMean30
CAD.tmaxMean30
EXC.tmaxMean30
PRO.tmaxMean30
TVT.tmaxMean30
TRS.tmaxMean30
DOR.tmaxMean30
INT.tmaxMean30
CTT.tmaxMean30
JAP.tmaxMean30
STM.tmaxMean30
PUR.tmaxMean30
FIT.tmaxMean30
CPR.tmaxMean30
TER.tmaxMean30
PUN.tmaxMean30
QUA.tmaxMean30
WAK.tmaxMean30
CQP.tmaxMean30
MEL.tmaxMean30
MOR.tmaxMean30
MIN.tmaxMean30
PSF.tmaxMean30
PSC.tmaxMean30
CIN.prcpMean30
VEX.prcpMean30
ABS.prcpMean30
CAD.prcpMean30
COM.prcpMean30
EXC.prcpMean30
PRO.prcpMean30
TVT.prcpMean30
TRS.prcpMean30
DOR.prcpMean30
INT.prcpMean30
CTT.prcpMean30
JAP.prcpMean30
STM.prcpMean30
PUR.prcpMean30
FIT.prcpMean30
CPR.prcpMean30
TER.prcpMean30
PUN.prcpMean30
QUA.prcpMean30
WAK.prcpMean30
CQP.prcpMean30
MEL.prcpMean30
MOR.prcpMean30
MIN.prcpMean30
PSF.prcpMean30
PSC.prcpMean30
CIN.tminMean90
VEX.tminMean90
ABS.tminMean90
CAD.tminMean90
COM.tminMean90
EXC.tminMean90
PRO.tminMean90
TVT.tminMean90
TRS.tminMean90
DOR.tminMean90
INT.tminMean90
CTT.tminMean90
JAP.tminMean90
STM.tminMean90
PUR.tminMean90
FIT.tminMean90
CPR.tminMean90
TER.tminMean90
PUN.tminMean90
QUA.tminMean90
WAK.tminMean90
CQP.tminMean90
MEL.tminMean90
MOR.tminMean90
MIN.tminMean90
PSF.tminMean90
PSC.tminMean90
CIN.tmaxMean90
VEX.tmaxMean90
ABS.tmaxMean90
CAD.tmaxMean90
COM.tmaxMean90
EXC.tmaxMean90
PRO.tmaxMean90
TVT.tmaxMean90
TRS.tmaxMean90
DOR.tmaxMean90
INT.tmaxMean90
CTT.tmaxMean90
JAP.tmaxMean90
STM.tmaxMean90

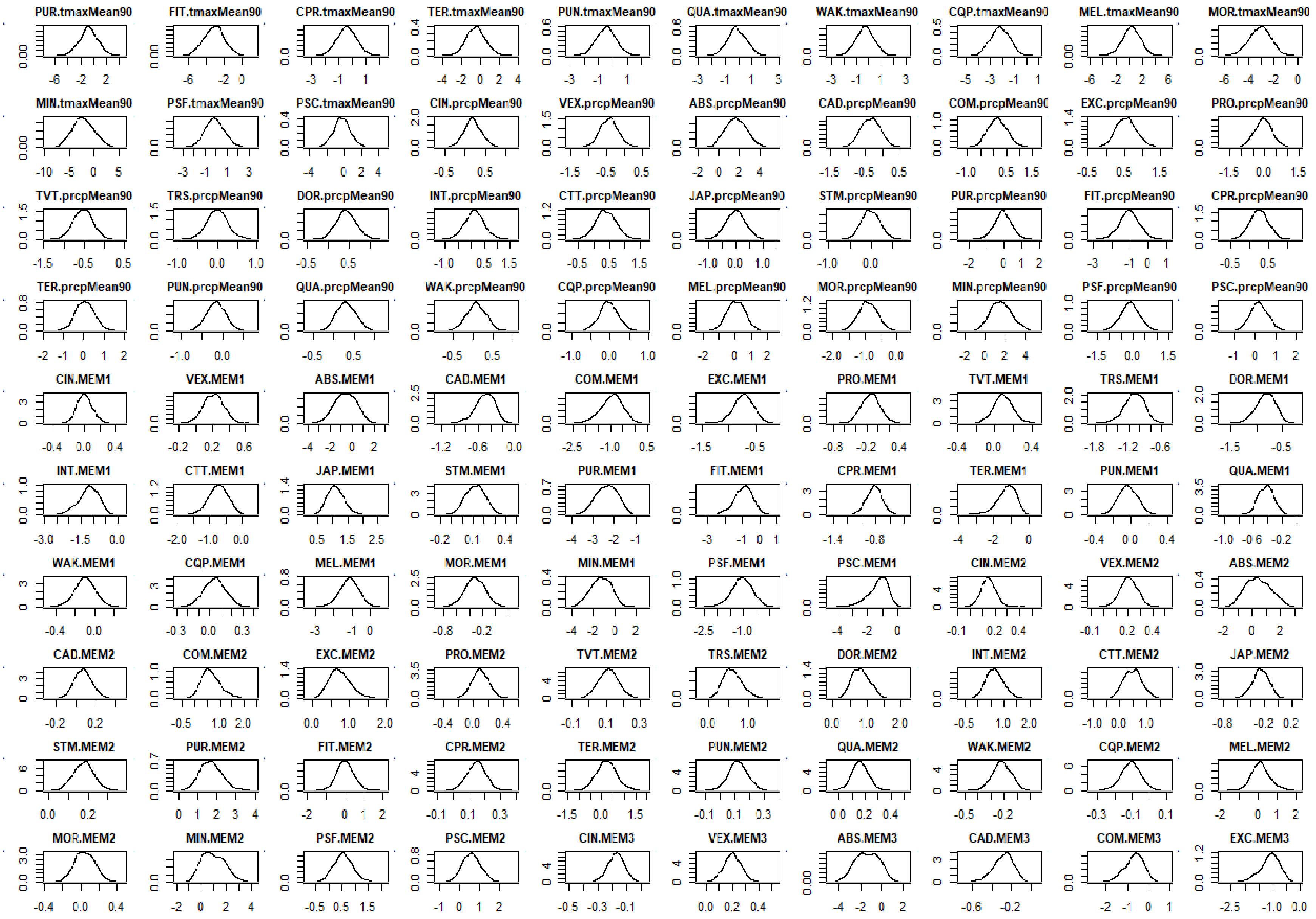
PUR.tmaxMean90
FIT.tmaxMean90
CPR.tmaxMean90
TER.tmaxMean90
PUN.tmaxMean90
QUA.tmaxMean90
WAK.tmaxMean90
CQP.tmaxMean90
MEL.tmaxMean90
MOR.tmaxMean90
MIN.tmaxMean90
PSF.tmaxMean90
PSC.tmaxMean90
CIN.prcpMean90
VEX.prcpMean90
ABS.prcpMean90
CAD.prcpMean90
COM.prcpMean90
EXC.prcpMean90
PRO.prcpMean90
TVT.prcpMean90
TRS.prcpMean90
DOR.prcpMean90
INT.prcpMean90
CTT.prcpMean90
JAP.prcpMean90
STM.prcpMean90
PUR.prcpMean90
FIT.prcpMean90
CPR.prcpMean90
TER.prcpMean90
PUN.prcpMean90
QUA.prcpMean90
WAK.prcpMean90
CQP.prcpMean90
MEL.prcpMean90
MOR.prcpMean90
MIN.prcpMean90
PSF.prcpMean90
PSC.prcpMean90
CIN.MEM1
VEX.MEM1
ABS.MEM1
CAD.MEM1
COM.MEM1
EXC.MEM1
PRO.MEM1
TVT.MEM1
TRS.MEM1
DOR.MEM1
INT.MEM1
CTT.MEM1
JAP.MEM1
STM.MEM1
PUR.MEM1
FIT.MEM1
CPR.MEM1
TER.MEM1
PUN.MEM1
QUA.MEM1
WAK.MEM1
CQP.MEM1
MEL.MEM1
MOR.MEM1
MIN.MEM1
PSF.MEM1
PSC.MEM1
CIN.MEM2
VEX.MEM2
ABS.MEM2
CAD.MEM2
COM.MEM2
EXC.MEM2
PRO.MEM2
TVT.MEM2
TRS.MEM2
DOR.MEM2
INT.MEM2
CTT.MEM2
JAP.MEM2
STM.MEM2
PUR.MEM2
FIT.MEM2
CPR.MEM2
TER.MEM2
PUN.MEM2
QUA.MEM2
WAK.MEM2
CQP.MEM2
MEL.MEM2
MOR.MEM2
MIN.MEM2
PSF.MEM2
PSC.MEM2
CIN.MEM3
VEX.MEM3
ABS.MEM3
CAD.MEM3
COM.MEM3
EXC.MEM3

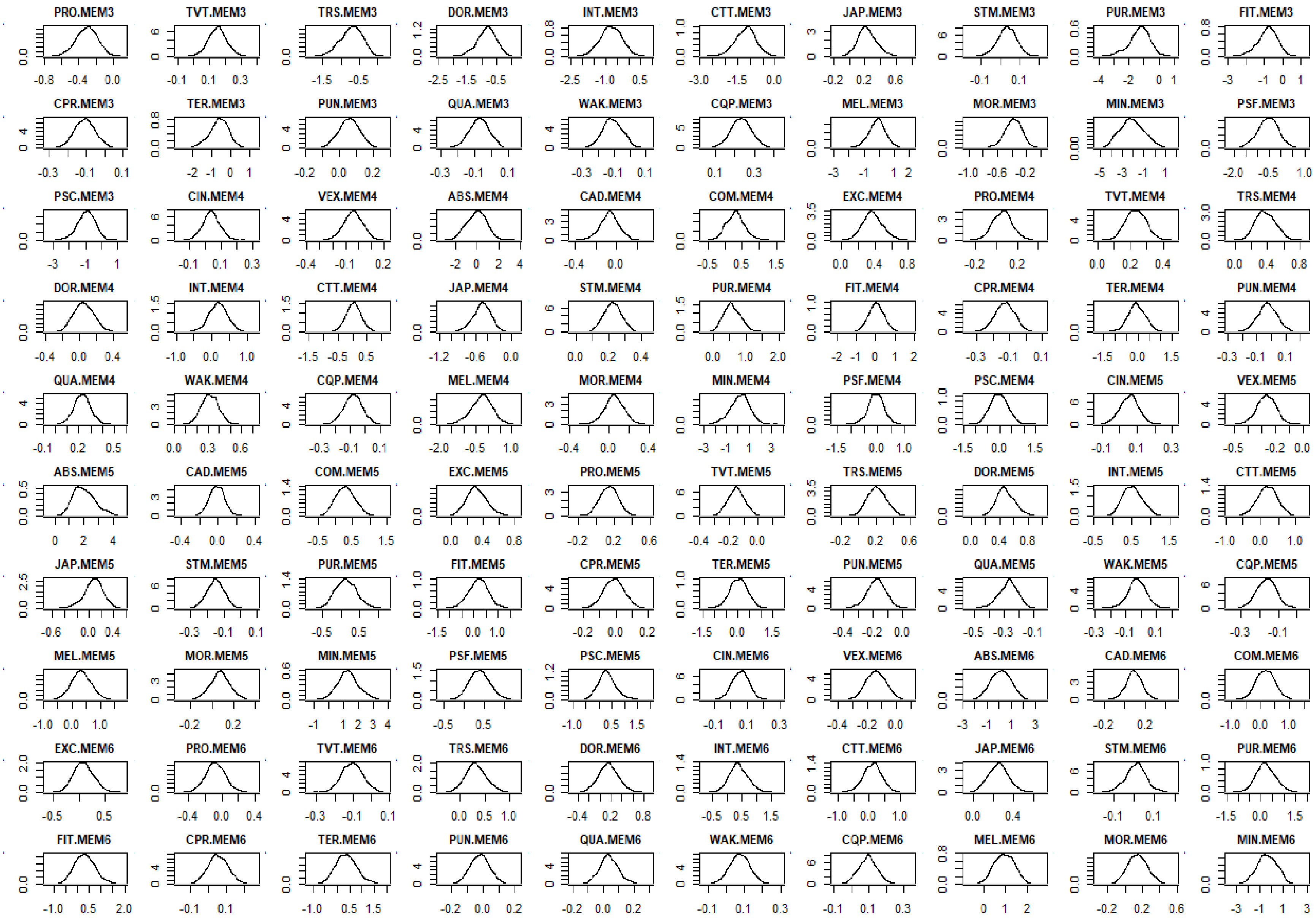

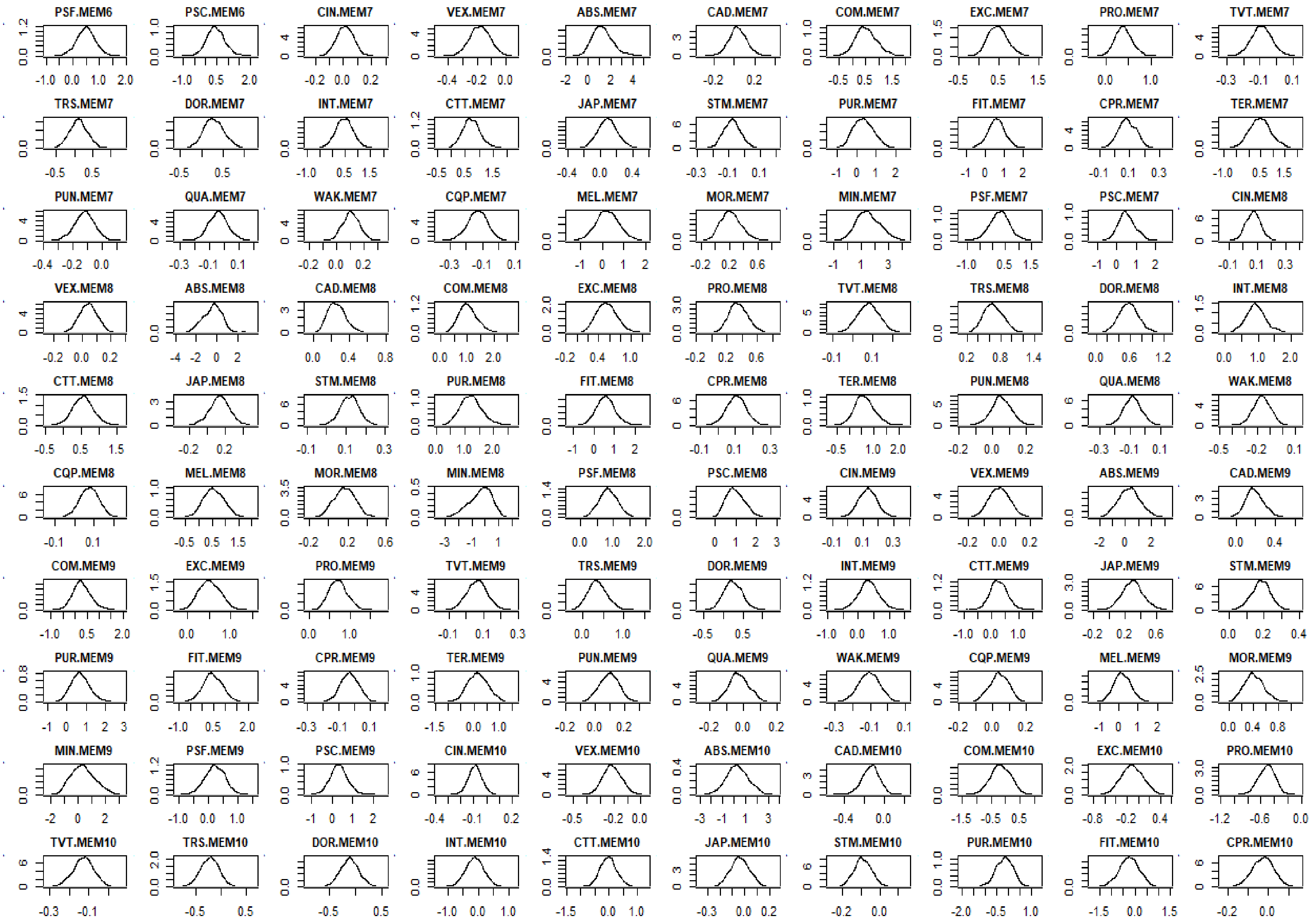
PSF.MEM6
PSC.MEM6
CIN.MEM7
VEX.MEM7
ABS.MEM7
CAD.MEM7
COM.MEM7
EXC.MEM7
PRO.MEM7
TVT.MEM7
TRS.MEM7
DOR.MEM7
INT.MEM7
CTT.MEM7
JAP.MEM7
STM.MEM7
PUR.MEM7
FIT.MEM7
CPR.MEM7
TER.MEM7
PUN.MEM7
QUA.MEM7
WAK.MEM7
CQP.MEM7
MEL.MEM7
MOR.MEM7
MIN.MEM7
PSF.MEM7
PSC.MEM7
CIN.MEM8
VEX.MEM8
ABS.MEM8
CAD.MEM8
COM.MEM8
EXC.MEM8
PRO.MEM8
TVT.MEM8
TRS.MEM8
DOR.MEM8
INT.MEM8
CTT.MEM8
JAP.MEM8
STM.MEM8
PUR.MEM8
FIT.MEM8
CPR.MEM8
TER.MEM8
PUN.MEM8
QUA.MEM8
WAK.MEM8
CQP.MEM8
MEL.MEM8
MOR.MEM8
MIN.MEM8
PSF.MEM8
PSC.MEM8
CIN.MEM9
VEX.MEM9
ABS.MEM9
CAD.MEM9
COM.MEM9
EXC.MEM9
PRO.MEM9
TVT.MEM9
TRS.MEM9
DOR.MEM9
INT.MEM9
CTT.MEM9
JAP.MEM9
STM.MEM9
PUR.MEM9
FIT.MEM9
CPR.MEM9
TER.MEM9
PUN.MEM9
QUA.MEM9
WAK.MEM9
CQP.MEM9
MEL.MEM9
MOR.MEM9
MIN.MEM9
PSF.MEM9
PSC.MEM9
CIN.MEM10
VEX.MEM10
ABS.MEM10
CAD.MEM10
COM.MEM10
EXC.MEM10
PRO.MEM10
TVT.MEM10
TRS.MEM10
DOR.MEM10
INT.MEM10
CTT.MEM10
JAP.MEM10
STM.MEM10
PUR.MEM10
FIT.MEM10
CPR.MEM10

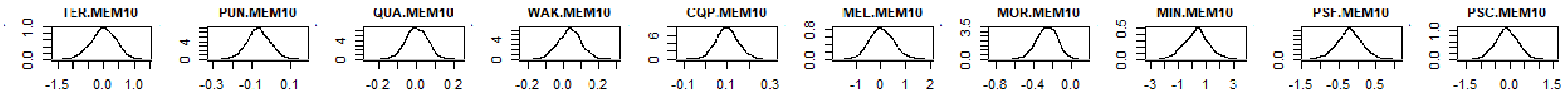
TER.MEM10
PUN.MEM10
QUA.MEM10
WAK.MEM10
CQP.MEM10
MEL.MEM10
MOR.MEM10
MIN.MEM10
PSF.MEM10
PSC.MEM10

## S19 Fig. Density plots of abundance – weather-only model

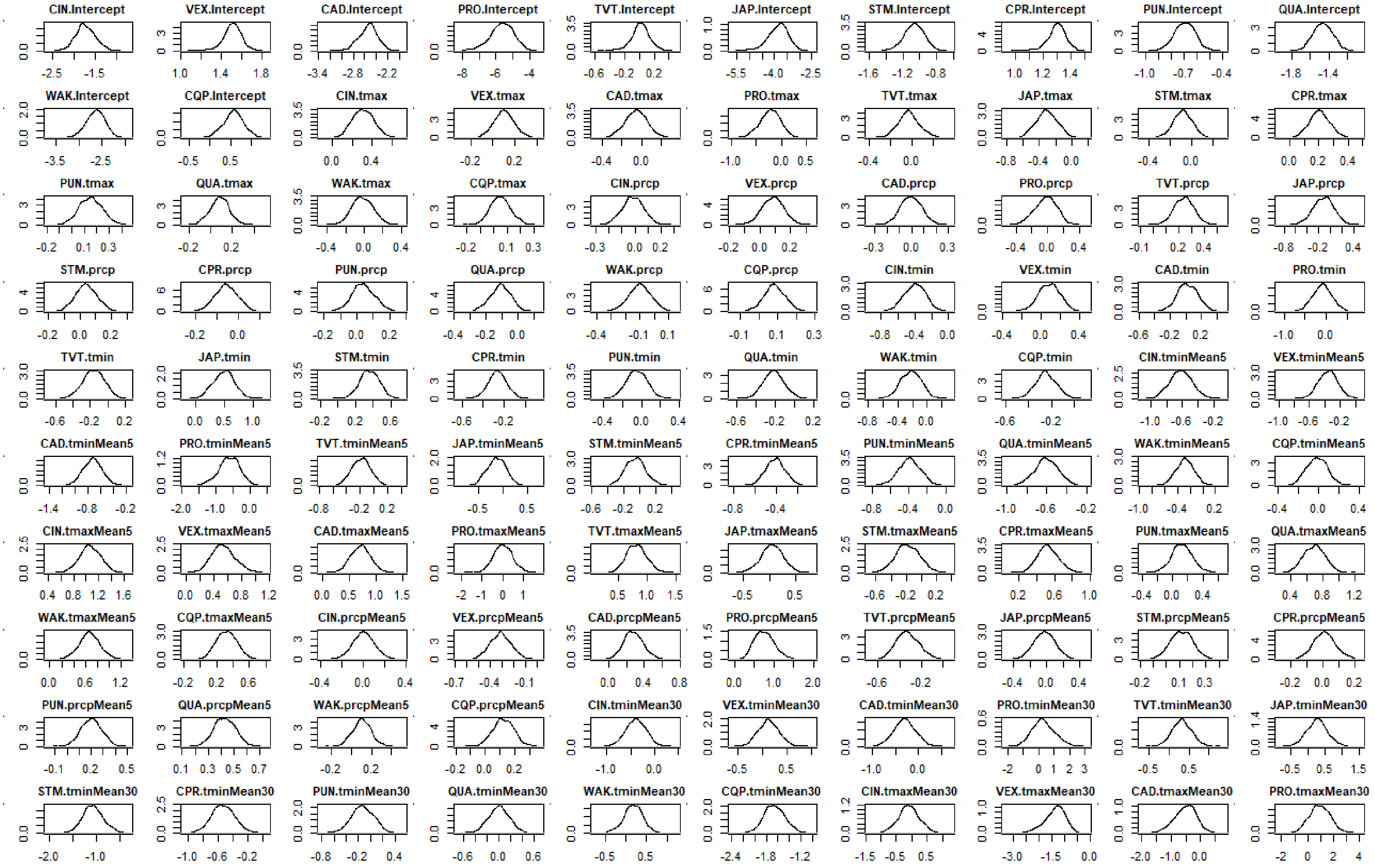

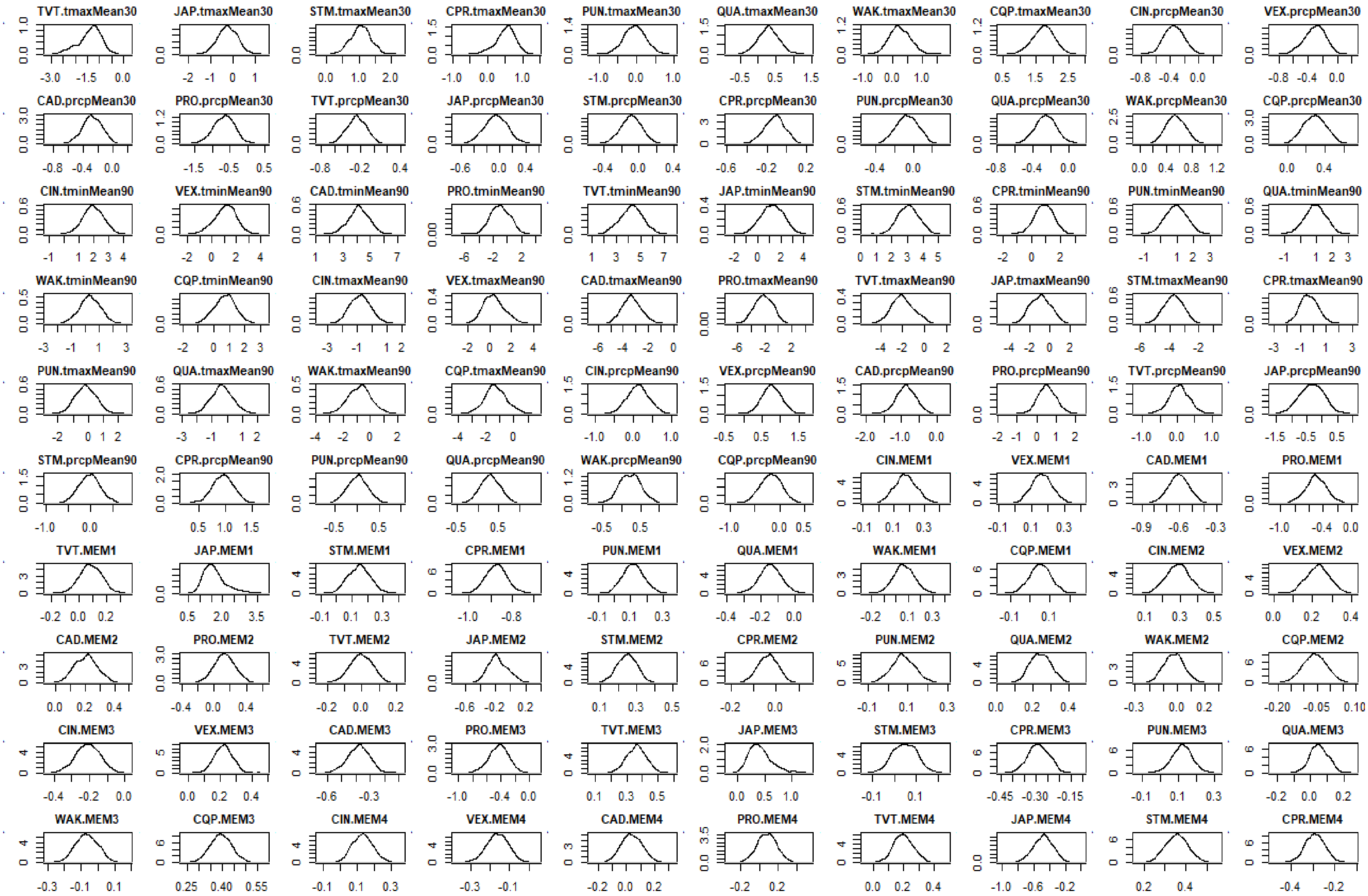
TVT.tmaxMean30
JAP.tmaxMean30
STM.tmaxMean30
CPR.tmaxMean30
PUN.tmaxMean30
QUA.tmaxMean30
WAK.tmaxMean30
CQP.tmaxMean30
CIN.prcpMean30
VEX.prcpMean30
CAD.prcpMean30
PRO.prcpMean30
TVT.prcpMean30
JAP.prcpMean30
STM.prcpMean30
CPR.prcpMean30
PUN.prcpMean30
QUA.prcpMean30
WAK.prcpMean30
CQP.prcpMean30
CIN.tminMean90
VEX.tminMean90
CAD.tminMean90
PRO.tminMean90
TVT.tminMean90
JAP.tminMean90
STM.tminMean90
CPR.tminMean90
PUN.tminMean90
QUA.tminMean90
WAK.tminMean90
CQP.tminMean90
CIN.tmaxMean90
VEX.tmaxMean90
CAD.tmaxMean90
PRO.tmaxMean90
TVT.tmaxMean90
JAP.tmaxMean90
STM.tmaxMean90
CPR.tmaxMean90
PUN.tmaxMean90
QUA.tmaxMean90
WAK.tmaxMean90
CQP.tmaxMean90
CIN.prcpMean90
VEX.prcpMean90
CAD.prcpMean90
PRO.prcpMean90
TVT.prcpMean90
JAP.prcpMean90
STM.prcpMean90
CPR.prcpMean90
PUN.prcpMean90
QUA.prcpMean90
WAK.prcpMean90
CQP.prcpMean90
CIN.MEM1
VEX.MEM1
CAD.MEM1
PRO.MEM1
TVT.MEM1
JAP.MEM1
STM.MEM1
CPR.MEM1
PUN.MEM1
QUA.MEM1
WAK.MEM1
CQP.MEM1
CIN.MEM2
VEX.MEM2
CAD.MEM2
PRO.MEM2
TVT.MEM2
JAP.MEM2
STM.MEM2
CPR.MEM2
PUN.MEM2
QUA.MEM2
WAK.MEM2
CQP.MEM2
CIN.MEM3
VEX.MEM3
CAD.MEM3
PRO.MEM3
TVT.MEM3
JAP.MEM3
STM.MEM3
CPR.MEM3
PUN.MEM3
QUA.MEM3
WAK.MEM3
CQP.MEM3
CIN.MEM4
VEX.MEM4
CAD.MEM4
PRO.MEM4
TVT.MEM4
JAP.MEM4
STM.MEM4
CPR.MEM4

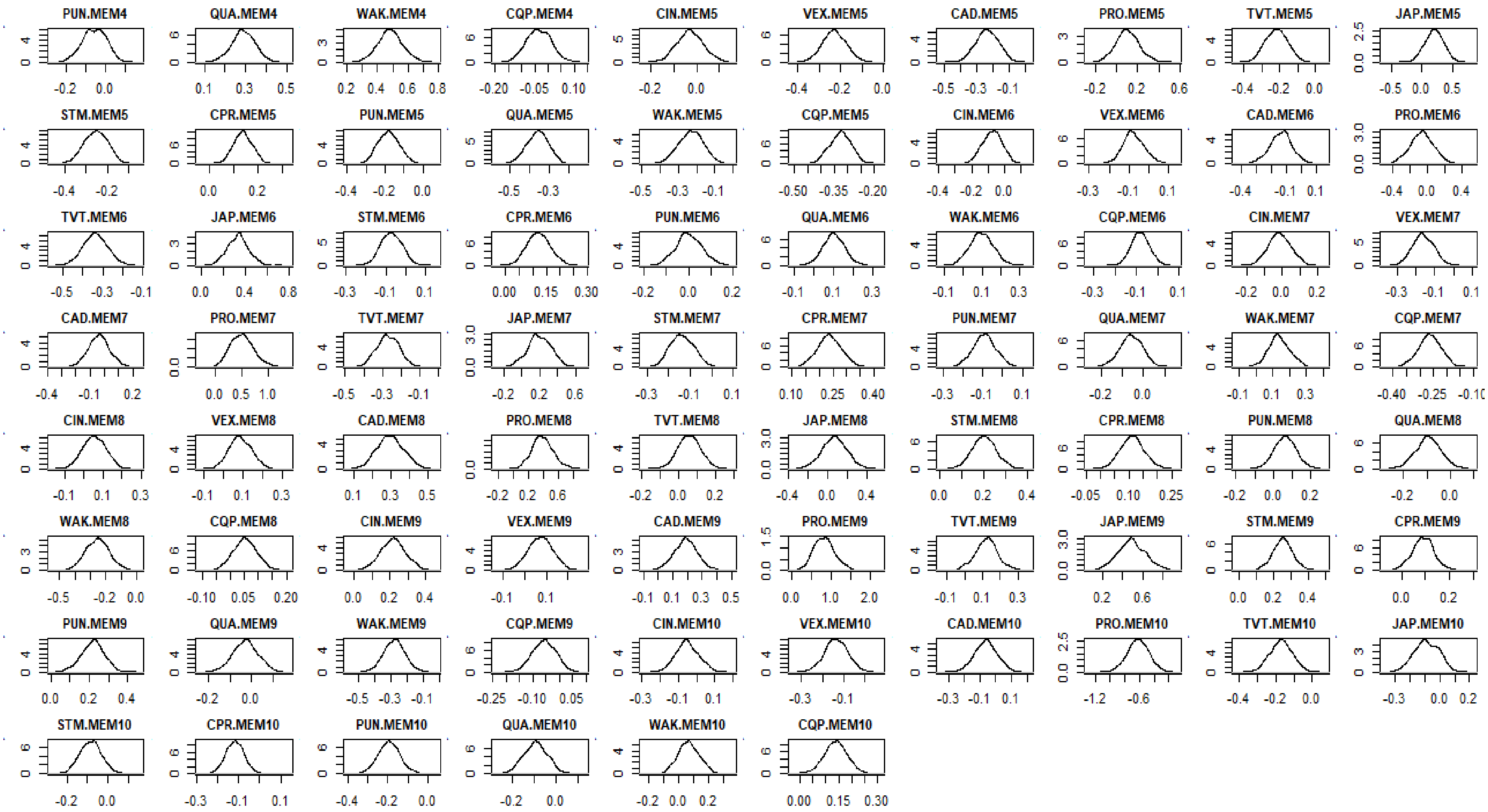
PUN.MEM4
QUA.MEM4
WAK.MEM4
CQP.MEM4
CIN.MEM5
VEX.MEM5
CAD.MEM5
PRO.MEM5
TVT.MEM5
JAP.MEM5
STM.MEM5
CPR.MEM5
PUN.MEM5
QUA.MEM5
WAK.MEM5
CQP.MEM5
CIN.MEM6
VEX.MEM6
CAD.MEM6
PRO.MEM6
TVT.MEM6
JAP.MEM6
STM.MEM6
CPR.MEM6
PUN.MEM6
QUA.MEM6
WAK.MEM6
CQP.MEM6
CIN.MEM7
VEX.MEM7
CAD.MEM7
PRO.MEM7
TVT.MEM7
JAP.MEM7
STM.MEM7
CPR.MEM7
PUN.MEM7
QUA.MEM7
WAK.MEM7
CQP.MEM7
CIN.MEM8
VEX.MEM8
CAD.MEM8
PRO.MEM8
TVT.MEM8
JAP.MEM8
STM.MEM8
CPR.MEM8
PUN.MEM8
QUA.MEM8
WAK.MEM8
CQP.MEM8
CIN.MEM9
VEX.MEM9
CAD.MEM9
PRO.MEM9
TVT.MEM9
JAP.MEM9
STM.MEM9
CPR.MEM9
PUN.MEM9
QUA.MEM9
WAK.MEM9
CQP.MEM9
CIN.MEM10
VEX.MEM10
CAD.MEM10
PRO.MEM10
TVT.MEM10
JAP.MEM10
STM.MEM10
CPR.MEM10
PUN.MEM10
QUA.MEM10
WAK.MEM10
CQP.MEM10

## S20. Density plots of abundance – weather-and-land-use model

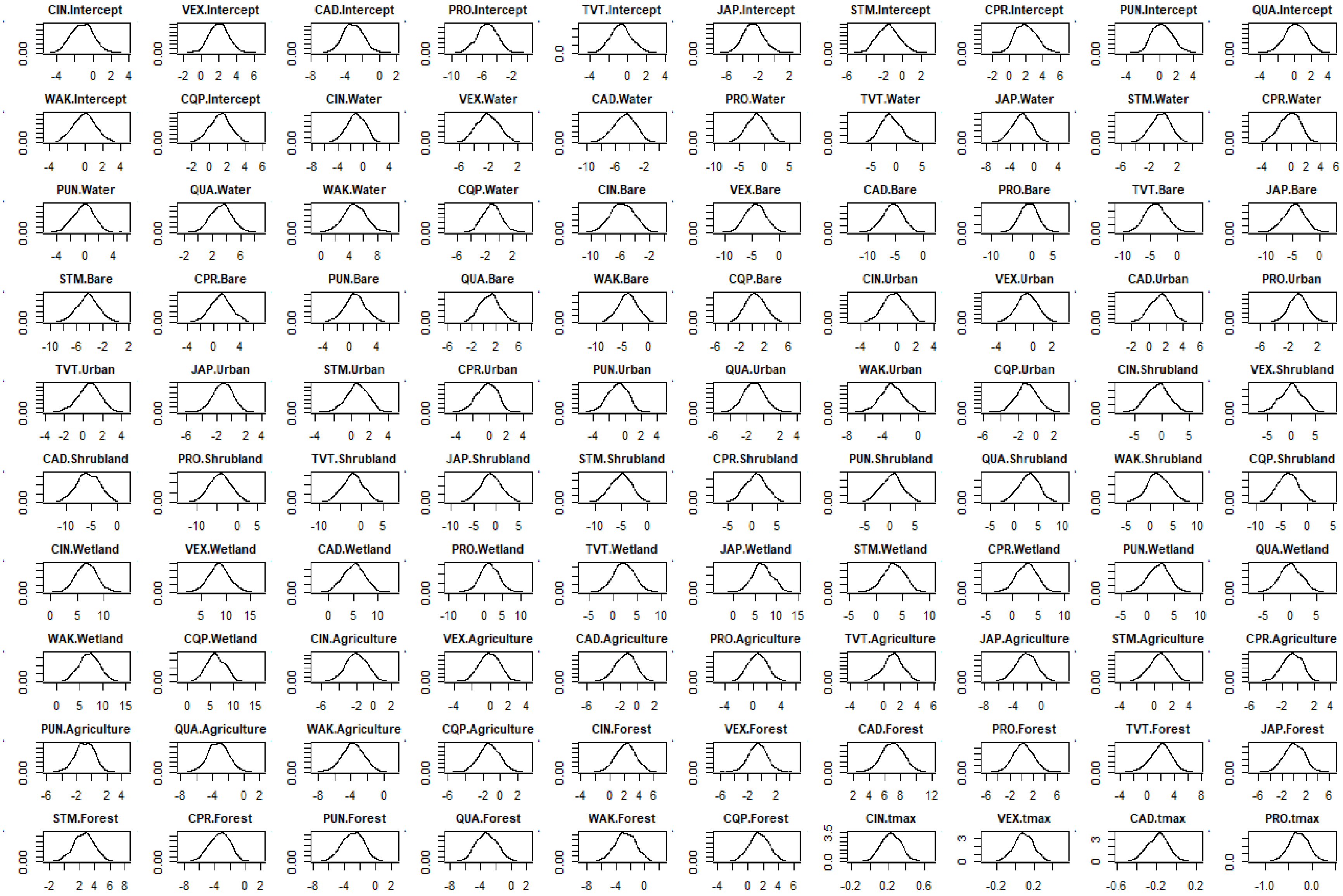

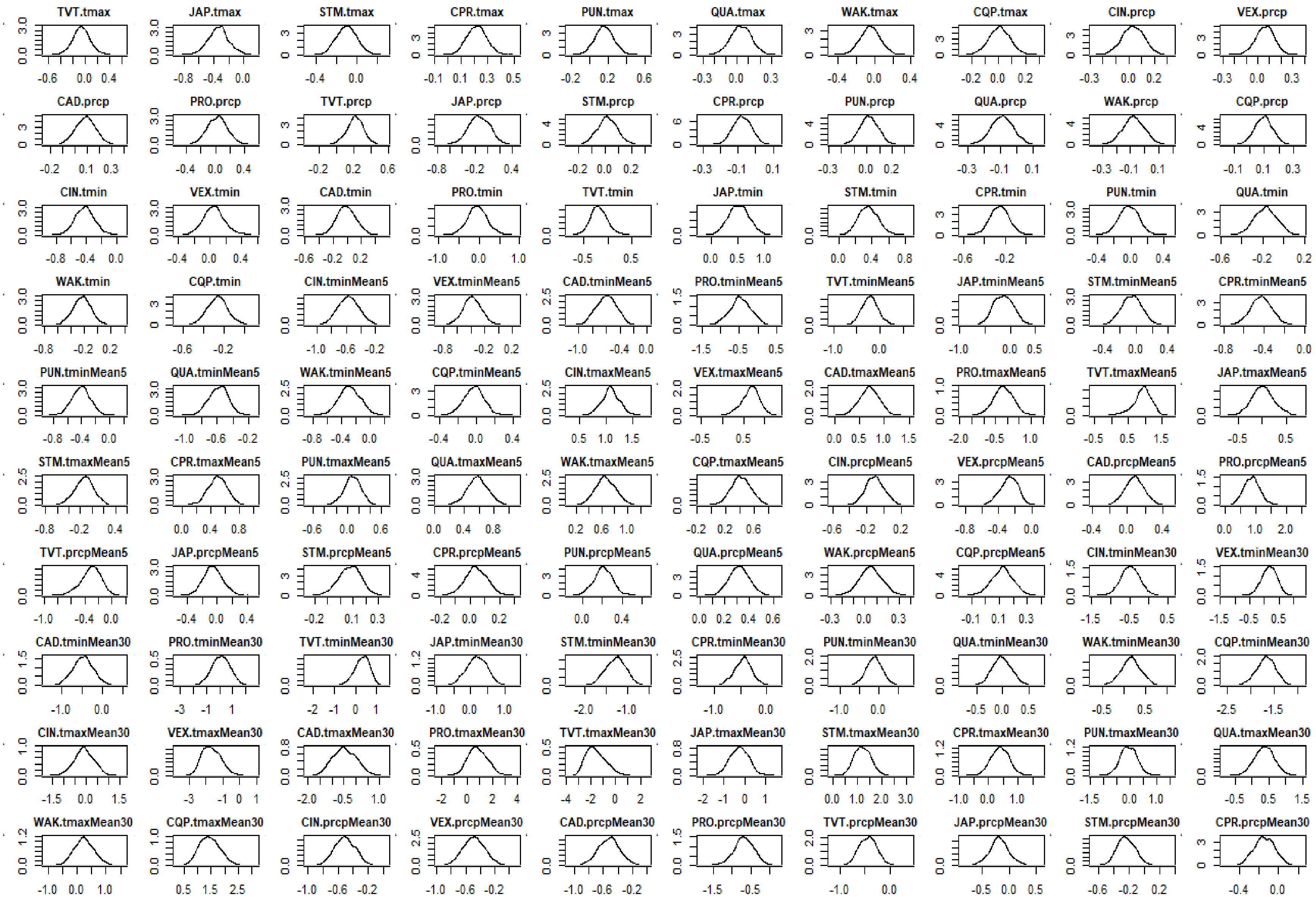
TVT.tmax
JAP.tmax
STM.tmax
CPR.tmax
PUN.tmax
QUA.tmax
WAK.tmax
CQP.tmax
CIN.prcp
VEX.prcp
CAD.prcp
PRO.prcp
TVT.prcp
JAP.prcp
STM.prcp
CPR.prcp
PUN.prcp
QUA.prcp
WAK.prcp
CQP.prcp
CIN.tmin
VEX.tmin
CAD.tmin
PRO.tmin
TVT.tmin
JAP.tmin
STM.tmin
CPR.tmin
PUN.tmin
QUA.tmin
WAK.tmin
CQP.tmin
CIN.tminMean5
VEX.tminMean5
CAD.tminMean5
PRO.tminMean5
TVT.tminMean5
JAP.tminMean5
STM.tminMean5
CPR.tminMean5
PUN.tminMean5
QUA.tminMean5
WAK.tminMean5
CQP.tminMean5
CIN.tmaxMean5
VEX.tmaxMean5
CAD.tmaxMean5
PRO.tmaxMean5
TVT.tmaxMean5
JAP.tmaxMean5
STM.tmaxMean5
CPR.tmaxMean5
PUN.tmaxMean5
QUA.tmaxMean5
WAK.tmaxMean5
CQP.tmaxMean5
CIN.prcpMean5
VEX.prcpMean5
CAD.prcpMean5
PRO.prcpMean5
TVT.prcpMean5
JAP.prcpMean5
STM.prcpMean5
CPR.prcpMean5
PUN.prcpMean5
QUA.prcpMean5
WAK.prcpMean5
CQP.prcpMean5
CIN.tminMean30
VEX.tminMean30
CAD.tminMean30
PRO.tminMean30
TVT.tminMean30
JAP.tminMean30
STM.tminMean30
CPR.tminMean30
PUN.tminMean30
QUA.tminMean30
WAK.tminMean30
CQP.tminMean30
CIN.tmaxMean30
VEX.tmaxMean30
CAD.tmaxMean30
PRO.tmaxMean30
TVT.tmaxMean30
JAP.tmaxMean30
STM.tmaxMean30
CPR.tmaxMean30
PUN.tmaxMean30
QUA.tmaxMean30
WAK.tmaxMean30
CQP.tmaxMean30
CIN.prcpMean30
VEX.prcpMean30
CAD.prcpMean30
PRO.prcpMean30
TVT.prcpMean30
JAP.prcpMean30
STM.prcpMean30
CPR.prcpMean30

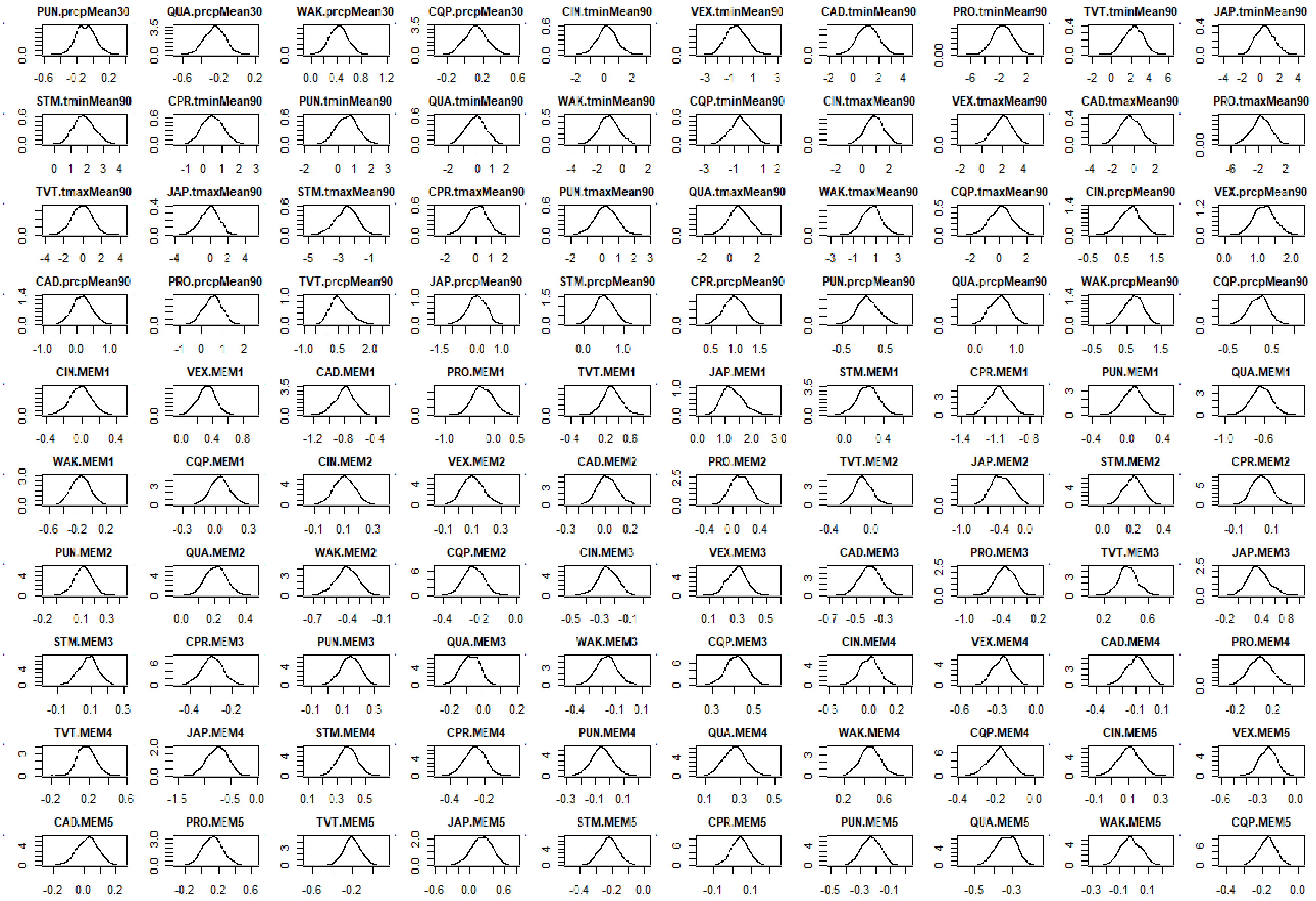
PUN.prcpMean30
QUA.prcpMean30
WAK.prcpMean30
CQP.prcpMean30
CIN.tminMean90
VEX.tminMean90
CAD.tminMean90
PRO.tminMean90
TVT.tminMean90
JAP.tminMean90
STM.tminMean90
CPR.tminMean90
PUN.tminMean90
QUA.tminMean90
WAK.tminMean90
CQP.tminMean90
CIN.tmaxMean90
VEX.tmaxMean90
CAD.tmaxMean90
PRO.tmaxMean90
TVT.tmaxMean90
JAP.tmaxMean90
STM.tmaxMean90
CPR.tmaxMean90
PUN.tmaxMean90
QUA.tmaxMean90
WAK.tmaxMean90
CQP.tmaxMean90
CIN.prcpMean90
VEX.prcpMean90
CAD.prcpMean90
PRO.prcpMean90
TVT.prcpMean90
JAP.prcpMean90
STM.prcpMean90
CPR.prcpMean90
PUN.prcpMean90
QUA.prcpMean90
WAK.prcpMean90
CQP.prcpMean90
CIN.MEM1
VEX.MEM1
CAD.MEM1
PRO.MEM1
TVT.MEM1
JAP.MEM1
STM.MEM1
CPR.MEM1
PUN.MEM1
QUA.MEM1
WAK.MEM1
CQP.MEM1
CIN.MEM2
VEX.MEM2
CAD.MEM2
PRO.MEM2
TVT.MEM2
JAP.MEM2
STM.MEM2
CPR.MEM2
PUN.MEM2
QUA.MEM2
WAK.MEM2
CQP.MEM2
CIN.MEM3
VEX.MEM3
CAD.MEM3
PRO.MEM3
TVT.MEM3
JAP.MEM3
STM.MEM3
CPR.MEM3
PUN.MEM3
QUA.MEM3
WAK.MEM3
CQP.MEM3
CIN.MEM4
VEX.MEM4
CAD.MEM4
PRO.MEM4
TVT.MEM4
JAP.MEM4
STM.MEM4
CPR.MEM4
PUN.MEM4
QUA.MEM4
WAK.MEM4
CQP.MEM4
CIN.MEM5
VEX.MEM5
CAD.MEM5
PRO.MEM5
TVT.MEM5
JAP.MEM5
STM.MEM5
CPR.MEM5
PUN.MEM5
QUA.MEM5
WAK.MEM5
CQP.MEM5

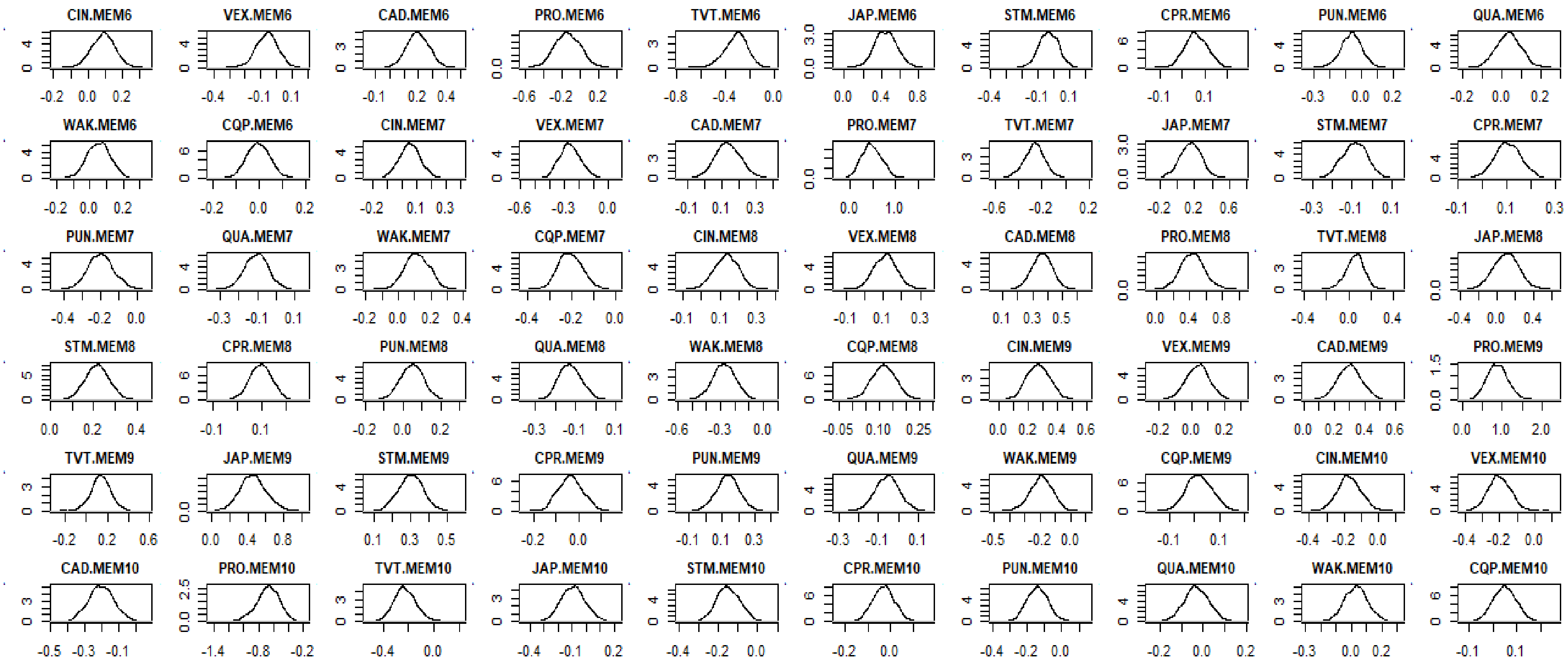
CIN.MEM6
VEX.MEM6
CAD.MEM6
PRO.MEM6
TVT.MEM6
JAP.MEM6
STM.MEM6
CPR.MEM6
PUN.MEM6
QUA.MEM6
WAK.MEM6
CQP.MEM6
CIN.MEM7
VEX.MEM7
CAD.MEM7
PRO.MEM7
TVT.MEM7
JAP.MEM7
STM.MEM7
CPR.MEM7
PUN.MEM7
QUA.MEM7
WAK.MEM7
CQP.MEM7
CIN.MEM8
VEX.MEM8
CAD.MEM8
PRO.MEM8
TVT.MEM8
JAP.MEM8
STM.MEM8
CPR.MEM8
PUN.MEM8
QUA.MEM8
WAK.MEM8
CQP.MEM8
CIN.MEM9
VEX.MEM9
CAD.MEM9
PRO.MEM9
TVT.MEM9
JAP.MEM9
STM.MEM9
CPR.MEM9
PUN.MEM9
QUA.MEM9
WAK.MEM9
CQP.MEM9
CIN.MEM10
VEX.MEM10
CAD.MEM10
PRO.MEM10
TVT.MEM10
JAP.MEM10
STM.MEM10
CPR.MEM10
PUN.MEM10
QUA.MEM10
WAK.MEM10
CQP.MEM10

# Reference


1. Ewing, D.A., et al., *Modelling the effect of temperature on the seasonal population dynamics of temperate mosquitoes.* Journal of theoretical biology, 2016. **400**: p. 65-79.

2. Roiz, D., et al., *Climatic effects on mosquito abundance in Mediterranean wetlands.* Parasites & vectors, 2014. **7**(1): p. 333.

3. Roiz, D., et al., *Landscape effects on the presence, abundance and diversity of mosquitoes in Mediterranean wetlands.* PLoS One, 2015. **10**(6): p. e0128112.

4. Britannica, T.E.o.E. *Mosquito*. June 02, 2020 [cited 2020 November 19, 2020]; Available from: https://www.britannica.com/animal/mosquito-insect.

5. Reiskind, M., et al., *Mosquitoes of field and forest: the scale of habitat segregation in a diverse mosquito assemblage.* Medical and veterinary entomology, 2017. **31**(1): p. 44-54.

6. Steiger, D.B.M., S.A. Ritchie, and S.G. Laurance, *Mosquito communities and disease risk influenced by land use change and seasonality in the Australian tropics.* Parasites & vectors, 2016. **9**(1): p. 387.

7. Bowden, S.E., K. Magori, and J.M. Drake, *Regional differences in the association between land cover and West Nile virus disease incidence in humans in the United States.* The American journal of tropical medicine and hygiene, 2011. **84**(2): p. 234-238.

8. Crowder, D.W., et al., *West Nile virus prevalence across landscapes is mediated by local effects of agriculture on vector and host communities.* PLoS One, 2013. **8**(1): p. e55006.

9. Gardner, A.M., R.L. Lampman, and E.J. Muturi, *Land use patterns and the risk of West Nile virus transmission in central Illinois.* Vector-Borne and Zoonotic Diseases, 2014. **14**(5): p. 338-345.

10. Andreadis, T.G., et al., *Epidemiology of West Nile virus in Connecticut: a five-year analysis of mosquito data 1999-2003.* Vector Borne Zoonotic Dis, 2004. **4**(4): p. 360-78.

11. Talbot, B., M. Ardis, and M.A. Kulkarni, *Influence of Demography, Land Use, and Urban Form on West Nile Virus Risk and Human West Nile Virus Incidence in Ottawa, Canada.* Vector-Borne and Zoonotic Diseases, 2019.
